# An investigation of cardiac vagal tone over time and its relation to vigilance performance: a growth curve modeling approach

**DOI:** 10.3389/fnrgo.2023.1244658

**Published:** 2023-12-11

**Authors:** Shannon P. D. McGarry, Brittany N. Neilson, Noelle L. Brown, Kaylin D. Strong, Eric T. Greenlee, Martina I. Klein, Joseph T. Coyne

**Affiliations:** ^1^Information Technology Division, U.S. Naval Research Laboratory, Washington, DC, United States; ^2^Operational Psychology Department, Naval Aerospace Medical Institute, Pensacola, FL, United States; ^3^Department of Psychological Sciences, Texas Tech University, Lubbock, TX, United States; ^4^Strategic Analysis, Inc., Arlington, VA, United States

**Keywords:** vigilance decrement, heart rate variability, individual differences, longitudinal growth curve modeling, cardiac vagal tone

## Abstract

**Introduction:**

Research over the last couple of decades has demonstrated a relationship between psychophysiological measures, specifically cardiac functions, and cognitive performance. Regulation of the cardiac system under parasympathetic control is commonly referred to as cardiac vagal tone and is associated with the regulation of cognitive and socioemotional states. The goal of the current study was to capture the dynamic relationship between cardiac vagal tone and performance in a vigilance task.

**Method/Results:**

We implemented a longitudinal growth curve modeling approach which unveiled a relationship between cardiac vagal tone and vigilance that was non-monotonic and dependent upon each person.

**Discussion:**

The findings suggest that cardiac vagal tone may be a process-based physiological measure that further explains how the vigilance decrement manifests over time and differs across individuals. This contributes to our understanding of vigilance by modeling individual differences in cardiac vagal tone changes that occur over the course of the vigilance task.

## Introduction

Heart rate variability (HRV) measures the time fluctuations between cardiac cycles. This fluctuation is influenced by both parasympathetic and sympathetic nervous system activity (Berntson et al., 1997). The parasympathetic nervous system controls autonomic, or involuntary functions when the body is at rest, like digestion, heart rate, and breathing, to name a few. This is in comparison to the sympathetic nervous system which, broadly speaking, controls the body's “fight or flight” response, as it mobilizes the body's response to environmental and psychological stressors. Both the parasympathetic and sympathetic nervous systems are a part of the autonomic nervous system. Given autonomic functioning is highly individualistic, its measures have been found to differentiate the efficacy of cognitive and emotional regulation (Porges, 2003), or in other words, “the ability to respond flexibly to changing demands” (Forte et al., 2019).

The vagus nerve is the main nerve of the parasympathetic nervous system, receiving an estimated 75% of its fibers, and is the main contributor for regulating cardiac functions (McCorry, 2007; Brodal, 2010). Regulation of the cardiac system under parasympathetic control is commonly referred to as *cardiac vagal tone* (Laborde et al., 2017). Research has connected cardiac vagal tone to the regulation of several psychological processes (e.g., Hansen et al., 2003; Porges, 2007; Duschek et al., 2009; Thayer et al., 2009). According to the neurovisceral integration model (Thayer et al., 2009), cardiac vagal tone is related to the regulation of activity in the prefrontal cortex. Specifically, the areas of the brain responsible for this regulation are also involved in cardiac functioning through the vagus nerve (see Forte et al., 2019 for review). Research supporting this theory includes the observed association of higher resting HRV, an index of high tonic cardiac vagal tone, and increased activity in the prefrontal cortex (Thayer et al., 2012). By contrast, hypoactive prefrontal functioning was associated with lower resting HRV, an index of lower tonic cardiac vagal tone (Thayer and Sternberg, 2006; Park and Thayer, 2014). Polyvagal theory similarly states that higher cardiac vagal tone is associated with improved social functioning and that this relationship has evolved to support fast, resilient responses to environmental demands that allow one to remain calm under stress (Porges, 2003). Research also showed that changes in cardiac vagal tone in response to cognitive stressors was associated with cognitive and emotional regulation abilities (Obradović and Finch, 2017).

As an attestation to the connection between cognitive and emotional control and cardiac vagal tone, stimulation of the vagus nerve is used in the treatment of several neurologic conditions. For instance, cervical transcutaneous vagal nerve stimulation (ctVNS) mitigated the effects of fatigue and reduced the negative impacts observed on arousal and multi-tasking (McIntire et al., 2021). Vagal nerve stimulation also had several benefits to cognitive performance, including memory and plasticity-enhancing effects; it is postulated this effect occurs because stimulation of the vagus nerve activates the locus coeruleus (Hulsey et al., 2017; McIntire et al., 2021), a brainstem nucleus strongly associated with the regulation of cognitive processes, including, arousal regulation, task engagement, attentional processing, and affective state (Foote et al., 1980; Aston-Jones and Bloom, 1981; Morrison and Foote, 1986; Sara and Segal, 1991; Aston-Jones et al., 1996; Nieuwenhuis et al., 2005; Robison and Brewer, 2022). The vagus nerve's association with both the brain areas that regulate prefrontal processes and those that regulate cardiac functions may serve as a source of the association between cardiac vagal tone and emotional and cognitive regulation. Notwithstanding, this relationship is likely to be complex and not fully explained by an association between a single region or neural pathway. Several brain areas are associated with the control of cognition and behavior (e.g., Critchley and Harrison, 2013) with lateralized and overlapping regions identified regarding sympathetic and parasympathetic functioning (Sturm et al., 2018). Beissner et al. (2013) applied activation likelihood estimation (ALE), to determine common activated regions across imaging studies, and found both a dissociation between the autonomic system (sympathetic and parasympathetic) and type of task (cognitive, affective, and somatosensory-motor). The findings of their meta-analysis contribute to the idea that dissociable brain regions support the regulation of sympathetic and parasympathetic functions, including cardiac vagal tone, in line with previous research (Cannon, 1929; Recordati, 2003). Though the direct mechanism by which the vagus nerve influences regulation of cognitive and emotional processes is not clear, it is apparent that cardiac vagal tone is positively associated with these processes, as it has been observed in several domains (Forte et al., 2019).

One prominent cognitive skill people are tasked with is monitoring information sources. Early interest in studying prolonged attention of people came from the observed performance degradations over time among radar operators during World War II. Research aimed to identify an optimal length of watch that resulted in radar operators maintaining their attentional performance (Mackworth, 1948). It continues to be widely studied, in large part due to the rise of automation in the information age (Wohleber et al., 2019). Many active roles have shifted the operator to be a passive observer, akin to radar operators. These aforementioned roles require vigilance, or paying attention to an information source in order to detect an infrequent event over a lengthy period of time. The decline in event detection and/or increase in response latency that is often experienced when tasked with responding to rare-event stimuli is referred to as the *vigilance decrement* (Parasuraman and Davies, 1976). In seminal work by Mackworth (1948), the decrement was most prominent after 30 min in a 2-h long task, but follow-up research found it could occur as soon as 5-min depending on task demands (e.g., Nuechterlein et al., 1983). In addition to studying the onset and magnitude of the decrement (Parasuraman et al., 1987; See et al., 1995), researchers have also investigated methods to mitigate it, like giving observers a break from monitoring (McCormack, 1958; Bergum and Lehr, 1962; Ariga and Lleras, 2011; Helton and Russell, 2012, 2015, 2017; Ross et al., 2014; Ralph et al., 2017).

An improved understanding of the underlying neurocognitive and psychophysiological associations with performance in vigilance tasks may be important to understanding the factors that influence the vigilance decrement and its mitigating factors. The exact mechanisms underlying the decrement are still debated. Neuroscience findings have implicated the right prefrontal cortex and nonadrenergic reticular formation in overall vigilance performance (whereas brain regions associated with the temporal decrement are not as well-known; Parasuraman, 2000), areas associated with executive functions and arousal regulation. These findings bolster existing theories of the vigilance decrement. Resource theory assumes the decrement is due to the high mental workload inherent in the task that depletes attentional resources over time (Hitchcock et al., 1999; Grier et al., 2003; Warm et al., 2008), whereas mindlessness theory suggests that the monotonous nature of the task over time lulls observers into an absent-minded state (Robertson et al., 1997; Manly et al., 1999). Mindlessness theory has been replaced by mind-wandering theory, which attributes the vigilance decrement to the redirection of attention to task-unrelated thoughts—not absent-mindedness (Smallwood and Schooler, 2006). Last, resource-control theory attributes the decrement to a failure in executive control processes where resources abscond away from the task and toward mind-wandering processes (Thomson et al., 2015). While these theories imply different mechanisms, they all agree that available cognitive resources devoted to the task are impacted with time on task.

For the purposes of this paper, we will simplify the existing vigilance theories as either one of task engagement that depletes resources or one of task disengagement that redirects resources elsewhere. Cardiac vagal tone is used as a measure of general cognitive and emotional regulation, so it does not further detail engagement, disengagement, and/or the reallocation of resources. Rather, our goal is to use cardiac vagal tone to better understand the relationship between cognitive and emotional regulation over time and vigilance performance. The aforementioned vigilance theories suggest that the cognitive ability of executive control and/or the emotional dimension of arousal may be a factor in the vigilance decrement. Across all theories, it is widely accepted that vigilance requires sustained attention (e.g., Mackworth, 1948; Parasuraman and Davies, 1976; Parasuraman et al., 1987; See et al., 1995) that draws upon available cognitive resources. According to the circumplex model of affect, valence (i.e., pleasantness), and arousal (i.e., alertness), are two independent neurological systems from which all emotions arise (Russell, 1980; Posner et al., 2005). Findings from vigilance paradigms have also shown that self-reported measures of arousal predict overall vigilance performance and the decrement (Shaw et al., 2010). The authors argued that increased arousal may have enhanced the attention toward the task and lead to improved performance. In comparison, Luna et al. (2022) found a greater vigilance decrement in individuals who experienced greater declines in executive control with time on task. These, and other findings (e.g., Robison and Nguyen, 2023), indicate both cognitive and emotional elements may underlie vigilance, which may relate to cardiac vagal tone (Laborde et al., 2017; Forte et al., 2019). Given that HRV can index cardiac vagal tone, which is associated with the neural substrates that regulate cognitive and emotional resources, both of which are proposed to be essential elements of vigilance, it stands to reason that cardiac vagal tone should correlate with vigilance performance. The authors note that such a correlation would not suggest evidence for one theory over another, but rather improve our understanding of how resources that support vigilance may change and/or fluctuate over time and the subsequent effect such dynamism has on vigilance task performance.

Our overarching research goals include understanding how cardiac vagal tone trends over the course of a vigilance task, how it changes when participants are given a break during the task, its relation to performance (i.e., the vigilance decrement) and/or task load. Overall, we set out to quantify the individual differences of cardiac vagal tone trends. Previous research found individual differences in HRV measures over time were due to external task demands and features (Smolders et al., 2012; Obradović and Finch, 2017; Blanck et al., 2019; Smith et al., 2019; Schwarck et al., 2021; Spangler et al., 2021; Tung et al., 2021; Benz et al., 2022), even with very low birth weight neonates (Padhye et al., 2009). Similarly, prior research has shown differences in autonomic activity in response to emotional stimuli when investigated as longitudinal trajectories—a feature not previously found when the autonomic activity was averaged across trials (e.g., Golland et al., 2014; Pasquini et al., 2022). Thus, autonomic functions including cardiac vagal tone are not likely to remain stable over long periods of time and have been used to measure individual differences in cognitive and emotional regulation. It should be noted that a myriad of additional variables underlie individual differences in cardiac vagal tone (e.g., age, sex assigned at birth, physical fitness, genetics, to name a few; Umetani et al., 1998; De Meersman and Stein, 2007) as well as vigilance performance (e.g., gender, personality, ability, participant engagement, motivation, coping skills, etc.; Eysenck, 1989; Rose et al., 2002; Matthews et al., 2010, 2017; Shaw et al., 2010; Neigel et al., 2017, 2018; Peltier and Becker, 2017; Claypoole et al., 2018; Teo et al., 2018; Rice and Greenlee, 2019; Robison and Nguyen, 2023). Given the exploratory nature of this investigation, we do not further explore these factors nor do we have a priori hypotheses on how cardiac vagal tone will trend during a vigilance task, before and after a break, for different magnitudes of the vigilance decrement and/or for different task loads, and if (and how) this all depends on the individual. Rather, we aim for this work's contribution to predominantly be the rich source of information that is gathered when we study psychophysiological measures as a *process* for each individual participant. Not only is this important for studying cardiac vagal tone, previous vigilance research has also suggested the need to study vigilance and its corresponding psychophysiological measures at the individual level, as a single, average trajectory for the whole sample of participants may not reflect each individual participant's trajectory (Smith et al., 2019), which greatly revises vigilance theory and its relevant applications. One way to conduct this type of analysis is with a method called growth curve modeling.

Growth curve modeling estimates trends over time based on how each individual trends over time (Curran et al., 2010). It also can include person-level measures, such as the magnitude of a person's vigilance decrement, to understand how these trends over time are moderated. To our knowledge, cardiac vagal tone during vigilance tasks has not been analyzed with growth curve modeling approaches, specifically. We believe growth curve modeling will accurately characterize how cardiac vagal tone trends over time, the effect of individual differences, and further inform the relation between cardiac vagal tone and the vigilance decrement. We expect the outcome of these analyses to emphasize that each person responds physiologically and, in turn, behaviorally, to the vigilance task, break, and resumption of a vigilance task differently. Importantly, we expect the growth curve models to detect and quantify these differences, which is in stark contrast to pairwise comparison methods. The latter cannot detect nor quantify trends at the individual level, given they are comparing mean values that are calculated from the entire sample of participants. In other words, we believe that cardiac vagal tone represents dynamic visceral functions related to cognition and requires an approach capable of capturing temporal changes. Growth curve modeling will not only accurately capture how cardiac vagal tone trends over time, but also assess how meaningful and distinct individual trends of cardiac vagal tone are during the aforementioned vigilance task paradigm.

Though the literature investigating vigilance and its decrement spans about 75 years, interest in the topic has not waned. There are a growing number of workplace settings where vigilance is embedded into the day-to-day activities of the job, such as positions within the Transportation Security Agency, long-haul truck drivers, and supervisory control operators in the military, to name a few. However, these exemplary environments are still suffering from the unintended consequences of vigilance tasks, which prompts new research initiatives, especially ones that include new measures and analysis techniques. The current research seeks to contribute to the vigilance knowledge base by exploring the changes in cardiac vagal tone over the entire span of a vigilance task and its corresponding relation to vigilance performance, while simultaneously studying the dependency they both have on the individual participant.

## Method

The reported data were collected as a part of one of the author's doctoral dissertation (Neilson, 2022). HRV and vigilance performance data were reanalyzed to address the current research questions.

### Participants

Thirty-two volunteers from Texas Tech University, including undergraduate and graduate students, faculty, and staff, participated in the study (28 women; age: *M* = 24 years, *SD* = 9 years) and were compensated $15 for their time. Participants were required to be at least 18 years old, have normal or correct-to-normal vision, no color vision deficits and no history of cardiac problems that may impact physiological recordings (Berntson et al., 1997).

### Materials

Tasks and images were presented on a computer monitor, and a standard keyboard was used to record responses and reaction times to the vigilance task. The vigilance task was administered through SuperLab 6 (Cedrus Corporation, 2020) software installed on a Dell Optiplex 7050 machine. The monitors were 469.9 × 269.88 mm with screen resolution of 1,920 × 1,080. Participants were seated about 48.5 cm from the monitor to ensure the stimuli were presented at approximately the same visual angle.

**Figure 1:**
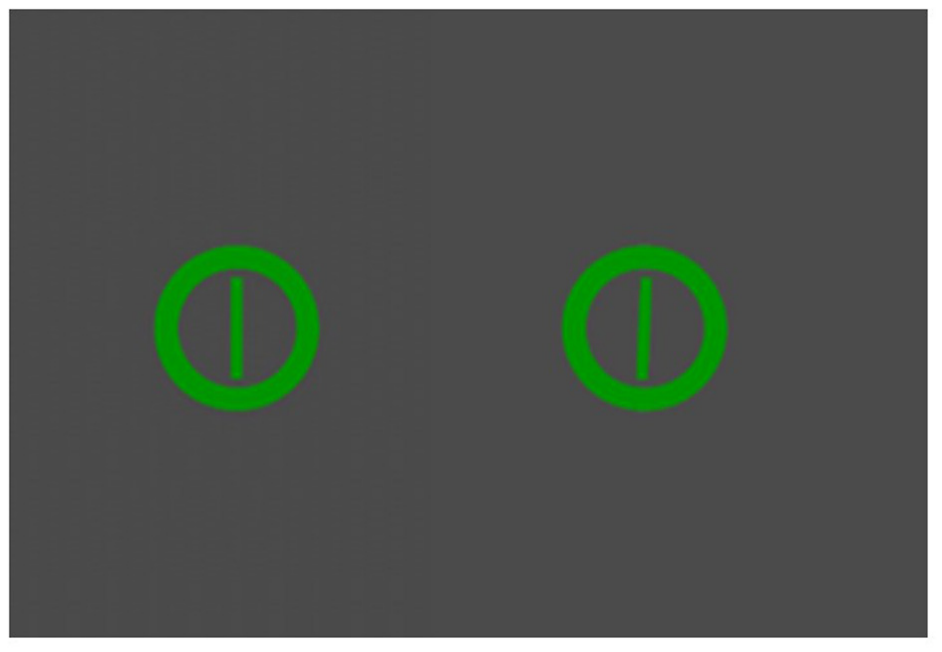
Visual representation of non-target pressure gauge **(left)** and target pressure gauge **(right)**. Adapted from Greenlee et al. (2015).

Participants performed a 2-D sensory vigilance task (Greenlee et al., 2015), in which they were required to monitor and respond to changes of a simulated pressure gauge. The needle (width = 0.4 cm, 0.47° visual angle) on the pressure gauge (height = 5.2 cm, 6.13° visual angle) was positioned at 12:00 to indicate safe pressure levels, representing a non-target that required no response. Unsafe pressure levels were indicated by a 2.5-degree clockwise movement of needle on the gauge, representing a target that warranted a response. The response was a spacebar click on the keyboard. The task involved presentation of an image of a gauge (either target or non-target) followed by a gray screen; this was deemed one event. Figure 1 demonstrates the difference between a target and non-target stimuli. Task load was manipulated via the event rate and inter-stimulus interval (see Table 1), with the target probability set to a constant 10%. Task load was manipulated by target events per minute (EPM) and the interstimulus interval (ISI; presentation of the gray screen) per time block. A target event rate above 40 is more demanding (Parasuraman and Giambra, 1991; Mouloua and Parasuraman, 1995; Rose et al., 2002) along with a variable ISI compared to a constant ISI (MacLean et al., 2009).

**Table 1:** Gauge monitoring vigilance task load condition.

**Stimulus presentations**		**Task load condition**	
		**Low**	**High**
Events per block	Total events	150	500
	Non-target events	135	450
	Target events	15	50
Trial presentations	Trial duration (ms)	4,000	1,200 (mean)
	Events per minute (EPM)	15	50 (mean)
	Gauge presentation (ms)	200	200
	ISI (ms)	3,800 (constant)	800, 900, 1,000, 1,100, 1,200 (varied)

**Figure 2:**
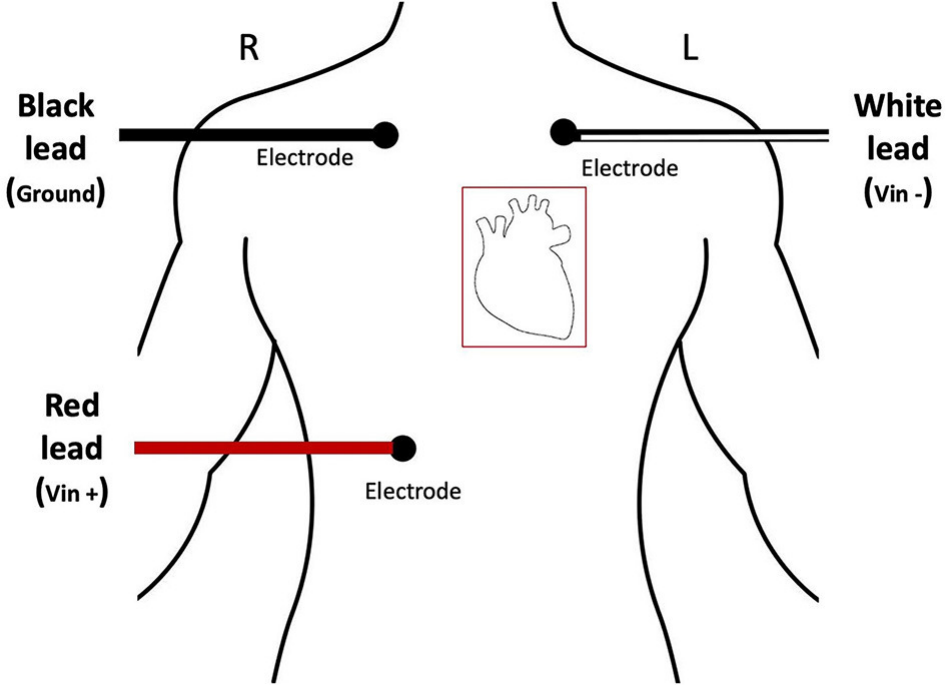
Diagram demonstrating accurate placement of ECG electrodes.

Electrocardiogram (ECG) signals were collected (200 Hz sample rate) on a separate Dell Optiplex 7050 machine using the Biopac MP150 data acquisition system. Biopac AcqKnowledge software (version 4.1.1) was used to process the ECG data and extract R-R time intervals. Kubios Heart Rate Variability Premium software (version 3.5.0) was used to compute HRV metrics from the R-R time intervals. Participants were fit with three disposable electrodes and electro-leads placed in a standard three lead configuration on their torsos as indicated in Figure 2. ECG data were collected continuously throughout the duration of the experiment with event markers automatically recorded to denote experimental time blocks.

### Procedure

Upon entering the lab, participants completed a COVID screening followed by informed consent. Participants were then instructed to appropriately place the ECG electrodes onto themselves with a demonstration by the researcher. Once ECG electrodes were properly placed, a 5-min ECG baseline was recorded while participants focused on a black screen on the computer monitor. Participants then completed questionnaires, which were outside the scope of the present work, followed by the gauge monitoring vigilance task. Participants were randomly assigned to the High or Low Task Load Condition.

**Figure 3:**
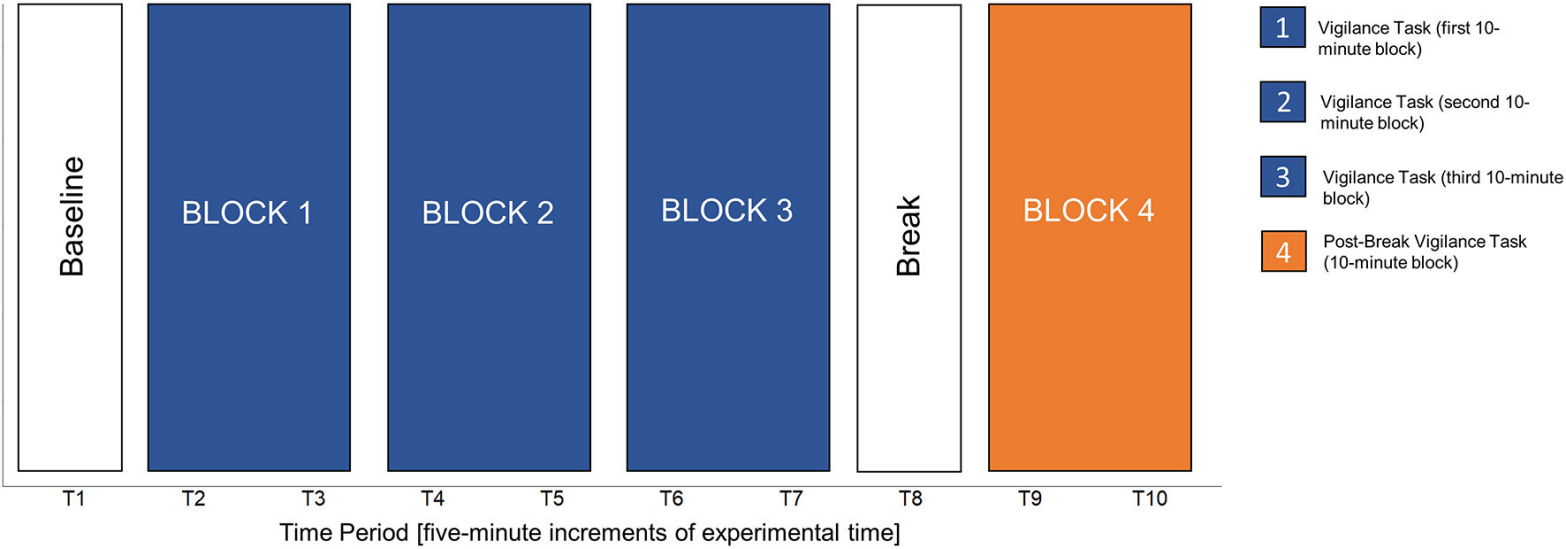
Schematic of the experiment.

Participants were provided instructions on how to perform the task and completed two practice sessions with feedback. Then, participants performed three, 10-min experimental blocks of the vigilance task. After the vigilance task, participants were presented with a 5-min and 48 s break that involved passively looking at nature or urban images, depending on the experimental condition, on the computer monitor. Prior analyses demonstrated that this break manipulation also resulted in no significant differences (Neilson, 2022), and therefore, the data from both break conditions were combined for the present work. A final, post-break 10-min vigilance task was then performed. The purpose of the post-break vigilance task was to assess whether the break benefitted vigilance task performance, by comparing Block 3 of the experimental vigilance task to the post-break vigilance task (i.e., Block 4). Each vigilance task (pre and post break) was broken down into 5-min time intervals for the HRV analyses (further rationale provided below). Figure 3 details the timing of the experiment. After both vigilance tasks ended, participants completed additional questionnaires, which were also outside the scope of the current research, removed ECG electrodes, and were debriefed.

### Data analytic approach

#### Behavioral data

Response latencies were trimmed to remove very fast trials that were akin to false starts and did not represent actual responses. Trials under 150 ms were removed given the inspection of correct responses suggested this latency was unlikely to represent actual responses, which resulted in the removal 0.620% of trials in the High Task Load Condition. The remaining responses were all slower than 350 ms. All of the response latencies in the Low Task Load Condition were slower than 350 ms and thus, not trimmed. These data were processed in R (version 4.2.2; R Core Team, 2022).

#### ECG

The Biopac Acqknowledge software was used to determine R-R peaks in the ECG data. The ECG data was first visually inspected to confirm the accuracy of automatic detection of the peaks, and manual changes were made as needed. R-R time intervals were exported using Biopac Acqknowledge software and then imported to Kubios for HRV computation. HRV data was analyzed in 5-min time intervals per HRV analysis guidelines (Task Force of the European Society of Cardiology and the North American Society of Pacing and Electrophysiology, 1996; Berntson et al., 1997). The Kubios automatic noise detection indicated that all signal noise was below 5%, indicating that each 5-min segment of data had <15 s of noise. Only three out of 320 (0.009%) 5-min segments of data had noise within this threshold, and that data was retained. The Kubios automatic beat correction algorithm (Lipponen and Tarvainen, 2019) was used to identify extra, missing, or misaligned heart beats. This algorithm removes extra heart beats and includes missing heart beats and then recomputes the R-R interval. It also interpolates new R-R intervals for misaligned beats (i.e., beats that are too short or too long). Again, the threshold of 5% was used, such that for each 5-min segment of data, <15 s included beat correction. The R-R interval data was detrending using a smoothness priors method and autoregressive (AR) modeling was used to compute HRV (Laborde et al., 2017).

We computed the root mean of squared successive differences, herein referred to as RMSSD, using Kubios. This measure computes the root mean square of successive time differences between heartbeats. This measure was chosen as it is a reliable measure of cardiac vagal tone, well grounded in HRV theory, and less influenced by respiration compared to other HRV measures (Laborde et al., 2017; Tung et al., 2021). It was then transformed with the natural log to satisfy the unequal variances assumption (e.g., Prinsloo et al., 2011; Laborde et al., 2017) and other modeling assumptions (Tung et al., 2021).

#### Growth curve modeling

Growth curve modeling estimates trends over time based on how each individual person trends over time (Curran et al., 2010). A first step is establishing a model that accurately estimates the dependent variable's trajectory over time; what is known as the *unconditional* growth curve model. Parameters are estimated to capture how the dependent variable is trending over time on average and assess the amount of variability in those estimates, as this determines if there are significant individual differences in the parameter estimates. In this work, the unconditional growth curve model estimates cardiac vagal tone trends during both vigilance tasks, how they change when participants are given a break, and any significant individual differences associated with each. Once a best fitting unconditional growth curve model is established, predictors can be added to understand how parameter estimates are moderated. This model is known as a *conditional* growth curve model and it is built by adding predictor(s) to specific or all parameter estimates in the established unconditional growth curve model. The predictors of interest for this work are change in the sensitivity metric known as *A* (Δ*A*), median response latency of hits (ΔHitRTMed), and task load. The conditional growth curve models inform how vigilance performance and task load are associated with the cardiac vagal tone trends previously established in the unconditional growth curve model and begin to offer an explanation on any individual differences that were observed. All growth curve modeling related analyses were conducted in R (version 4.0.5; R Core Team, 2021). Growth curve models were built via multilevel modeling (i.e., mixed effects modeling, hierarchical linear modeling, etc.; Curran et al., 2010; Hall, 2022).

## Results

### Behavioral performance

To verify our paradigm created a vigilance decrement as it is traditionally defined (e.g., an ultimate, significant decline in performance over time), we compared mean target detection rates and response latencies across the vigilance task. For comparison purposes, performance was broken down into four 10-min Blocks, with Blocks 1–3 occurring before the break and Block 4 occurring after the break. Significance was set at α = 0.05. Bonferroni corrections were applied to account for multiple comparisons.

Target detection was calculated as *A*, a measure of sensitivity (see, Zhang and Mueller, 2005). A 2 (Task Load Condition) × 4 (Block) Analysis of Variance (ANOVA) was conducted on *A*. There were main effects of Block, *F*_(3, 90)_ = 4.26, *p* = 0.007, $\eta_{\text{p}}^{2}$ = 0.124 and Task Load Condition, *F*_(1, 30)_ = 23.76, *p* < 0.001, $\eta_{\text{p}}^{2}$ = 0.442. The interaction was not significant, *F*_(3, 90)_ = 0.89, *p* = 0.450, $\eta_{\text{p}}^{2}$ = 0.029. Sensitivity was higher in Block 1 compared to Block 3 (*p* = 0.022) and Block 4 (*p* = 0.040) and the Low Task Load Condition outperformed the High Task Load Condition. No other comparisons were significant.

We compared median response latencies in a 2 (Task Load Condition) × 4 (Block) ANOVA with a Greenhouse Geiser correction for sphericity. There were main effects of Block, *F*_(2, 61.85)_ = 3.33, *p* = 0.043, $\eta_{\text{p}}^{2}$ = 0.097 and Task Load Condition, *F*_(1, 31)_ = 10.94, *p* = 0.002, $\eta_{\text{p}}^{2}$ = 0.261, showing faster latencies in the High Task Load Condition compared to the Low Task Load Condition. The interaction was not significant, *F*_(2, 61.85)_ = 0.50, *p* = 0.610, $\eta_{\text{p}}^{2}$ = 0.016. Follow-up pairwise comparisons between Block with Bonferroni correction revealed moderately slower latencies in Block 3 than Block 1, *t*_(32)_ = −2.75, *p* = 0.058. There were no statistically significant differences (*p* > 0.05 for all comparisons). Table 2 summarizes these findings.

**Table 2:** Summary of the vigilance performance results.

**Measure**	**Block**	**Task load condition**	
		**High task load** ***M*** **(SD)**	**Low task load** ***M*** **(SD)**
Target sensitivity (*A*)	Block 1	0.86 (0.03)	0.94 (0.05)
	Block 2	0.84 (0.04)	0.92 (0.06)
	Block 3	0.84 (0.03)	0.91 (0.07)
	Block 4	0.85 (0.04)	0.91 (0.06)
Avg. median response latency (ms)	Block 1	636 (66)	702 (93)
	Block 2	660 (59)	743 (119)
	Block 3	673 (75)	770 (128)
	Block 4	642 (86)	745 (103)

### Growth curve modeling of cardiac vagal tone

**Figure 4:**
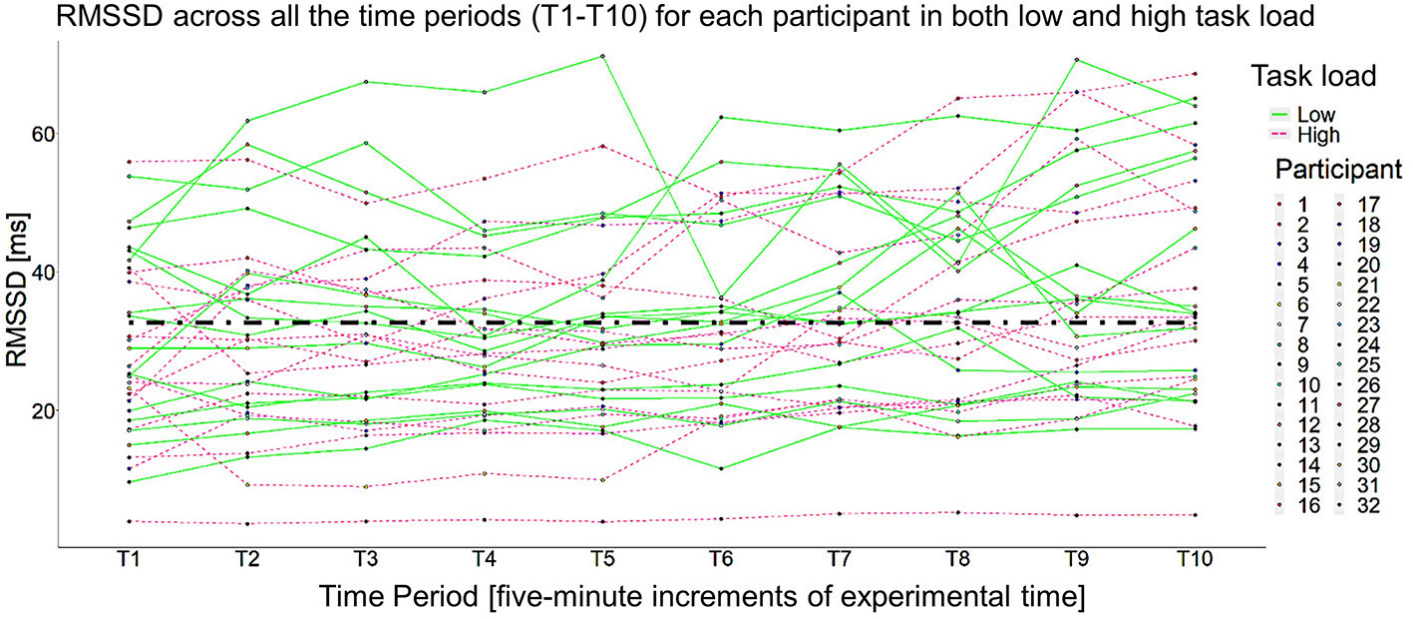
Spaghetti plot of all 32 participants across both low and high task load. Just as detailed in Figure 3, T1 was the 5-min baseline, T2–T7 are the six, 5-min time periods in the vigilance task (two time periods per block), T8 was the first 5 min of the break, and T9–T10 were the two, 5-min timepoints in the post-break vigilance task.

Unconditional and conditional growth curve models are defined by their fixed and random effects. In this work, *fixed effects* are parameter estimates for the “average participant” in the sample and there is only one estimated value for all participants in the sample. Specifically, the fixed effects estimated how cardiac vagal tone (i.e., the natural logarithm of RMSSD) trended over the course of the different experimental blocks (see Figure 3). *Random effects* estimate the parameter for each participant, which in turn quantifies the variability associated with the fixed effect parameter estimate. With model comparisons, we assess if that variance is significantly >0. If so, then that parameter is said to be a random effect, which means each individual participant has their own, unique estimate for that parameter. In total, random effects suggest there is evidence of significant individual differences for that parameter, meaning a fixed effect parameter is not a sufficient characterization of the dependent variable (Mirman, 2014). In this work, random effects were implemented to assess the presence of individual differences in cardiac vagal tone trends over the course of a vigilance task, break, and post-break vigilance task. In growth curve modeling, fixed and random effects consist of time slopes, which in this work, were composed of the consecutive 5-min time periods throughout the experiment. All models included piecewise time slopes because the experiment had a priori demarcations (i.e., baseline, the vigilance task, the break, and the post-break vigilance task). The slopes were centered on the first time period (T1), which was when baseline cardiac vagal tone was measured, making this the intercept parameter of all estimated models. All growth curve models were modeled as a general linear multilevel model and built with R packages lme4 (Bates et al., 2015) and nlme (Pinheiro et al., 2023) as needed. The degrees of freedom and the corresponding *p*-values for each parameter estimate in the general linear multilevel models relied on Satterthwaite's method (via the R package lmerTest). In the interest of brevity, the likelihood ratio test results for each model compared are reported, but only the details from the best fitting growth curve model are thoroughly discussed. All pertinent details about the growth curve modeling process can be reviewed in the link that is provided in the Supplementary material. Both Task Load Conditions were modeled in a single model given the spaghetti plot did not show a clear separation between the two, as seen in Figure 4.

One participant (Participant 32) was removed from the analysis given nearly impossible and nonvarying RMSSD values (see the line that is nearly flat across the entire session at the bottom of Figure 4). No other outliers were detected. The intraclass correlation (as calculated from an empty means, random intercept only model) was 0.830 [−2ΔLL(~1) = 423.37, *p* < 0.001], indicating that 83.0% of the RMSSD variance was due to person mean differences (i.e., random intercept variation), whereas 17.0% was due to within-person residual variation over time. This led to including a random intercept in all subsequent models. Several versions of unconditional growth curve models were estimated. Model selection was done via a maximal model, backwards-selection approach, meaning the most maximal model feasible was fit and iteratively compared to less maximal models. This approach minimized Type I error rates while maintaining maximal power (Barr et al., 2013; Matuschek et al., 2017). The basis of the most maximal model was the saturated means, unstructured variance model (Hoffman, 2015, p. 124). The most maximal model was then compared to a less maximal model (e.g., one with a reduced random effects structure), which in this work means reducing a time slope from a random effect to a fixed effect (see Barr et al., 2013 for more details). The significance of fixed effects was evaluated via their Wald test *p*-values, and the significance of random effects was evaluated via −2ΔLL tests (i.e., likelihood ratio tests using degrees of freedom equal to the difference in the number of estimated parameters), with significance set at α = 0.05. To compare non-nested models, the Bayesian information criteria (BIC) was used. All models were fitted with restricted maximum likelihood (REML) estimation unless their BIC values needed to be compared, then the model was refitted with maximum likelihood (ML) estimation. Singular models (i.e., models where the variance of at least one random effect was estimated as non-positive) were not considered as the likelihood of the model is no longer comparable (Hoffman, 2015, p. 198), Rather, the random effects structure of singular models was reduced and refit for comparison. To describe the size of the random variation around each effect, 95% confidence intervals were computed as: fixed effect ±1.96 × SQRT (random variance). Finally, the within-person residual variance was specified as constant over time with no residual covariance over time after accounting for any between-person random effects variances and covariances in all models.

#### Unconditional growth curve model

**Figure 5:**
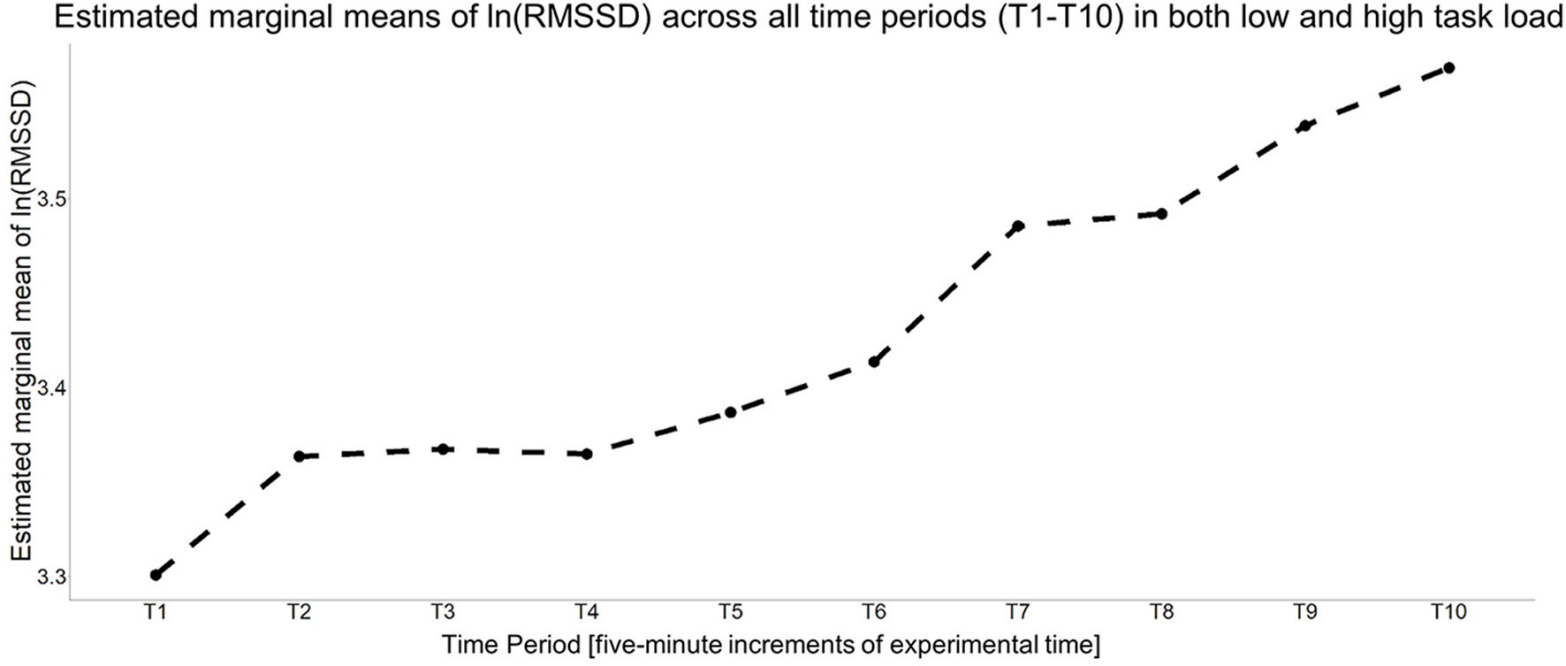
Estimated marginal means of a saturated means, unstructured variance model for cardiac vagal tone. It is estimating a non-monotonic increase in cardiac vagal tone, on average, over the course of the vigilance task, break, and post-break vigilance task.

Originally, there were five piecewise slopes in the unconditional growth curve model that map to the different parts of the experiment (see Figure 3). Specifically, they consisted of the following: T1–T2 (baseline and the first time period in the Block 1), T2–T7 (Blocks 1–3 of the vigilance task), T7–T8 (the last time period in the vigilance task and the break), T8–T9 (the break and the first time period in Block 4), and T9–T10 (Block 4). Figure 5 shows the estimated marginal means at each time period based on the saturated means, unstructured variance model.

Figure 5 depicts a general increase in RMSSD over the course of the experiment, one where the rate was increased upon starting the vigilance task (T1–T2), during the latter part of it (i.e., T4–T7), and then finally again during the post-break vigilance task (T9–T10). Therefore, the maximal growth curve model is a random time slope model, meaning all piecewise time slopes and the intercept are estimated as random effects. Also, per the estimated marginal means at each time period of the saturated means, unstructured variance model, both a random linear and quadratic slope was estimated for T2–T7. However, this model was singular, so it was not considered for comparison. The most maximal, non-singular model consisted of having a fixed quadratic time slope for T2–T7, a fixed linear time slope for T9–T10, with all else being random time slopes, and a random intercept. Therefore, this model was used as the baseline model for the backwards selection process, where the goal was to see if a more parsimonious model was a significantly worse fit. Each of the remaining random time slopes were individually removed and compared to this model via a −2ΔLL test. Making the slope for T7–T8 a fixed effect did not significantly worsen model fit [−2ΔLL(~5) = 4.482, *p* = 0.482]. Reducing any other random time slopes either led to a singular model or significantly worse model fit (all *p* < 0.007). Given it was more parsimonious and not a significantly worst fit, the new baseline model was one with a fixed quadratic time slope for T2–T7, a fixed linear time slope for T7–T8, a fixed linear time slope for T9–T10, all else as random time slopes/effects. Reducing any other time slope led to significantly worse model fit (*p* < 0.004). There was no significant difference between the predicted means over time of this model and that given by the saturated means, unstructured variance model [*F*_(3, 180)_ = 0.228, *p* = 0.877], but there was a difference in its predicted variance over time (−2ΔLL = 73.103, *p* = 0.004). In an attempt to better match the model of the variance estimated by the saturated means, unstructured variance model, several slight variations of the previous model were fit. Specifically, the piecewise slope for T2–T7 was broken up into two different piecewise time slopes (e.g., a model with all the original piecewise time slopes, but now slope T2–T7 had one slope for T2–T3 and then another slope for T3–T7; another model that was identical to the above, except now the two slopes for T2–T7 was a slope for T2–T4 and a slope from T4–T7, etc.). This investigation led to two unconditional growth curve models where the model for the means and the model for the variance did not significantly differ from what was estimated by the saturated means, unstructured variance model (all *p* > 0.05):

1. An unconditional growth curve model that had a random time slope for T1–T2, a random quadratic and linear time slope for T2–T6, a random time slope for T8–T9, a fixed time slope for T6–T7, a fixed time slope for T7–T8, and a fixed time slope for T9–T10 [*F*_(2, 179.999)_ = 0.073, *p* = 0.930; −2ΔLL = 50.854, *p* = 0.097].
2. An unconditional growth curve model that had a random time slope for T1–T2, a random quadratic time slope for T5–T7, a random time slope T8–T9, a fixed time slope T2–T5, a fixed time slope T7–T8, and a fixed time slope for T9–T10 [*F*_(2, 150.502)_ = 0.201, *p* = 0.818; −2ΔLL = 46.415, *p* = 0.193].

**Figure 6:**
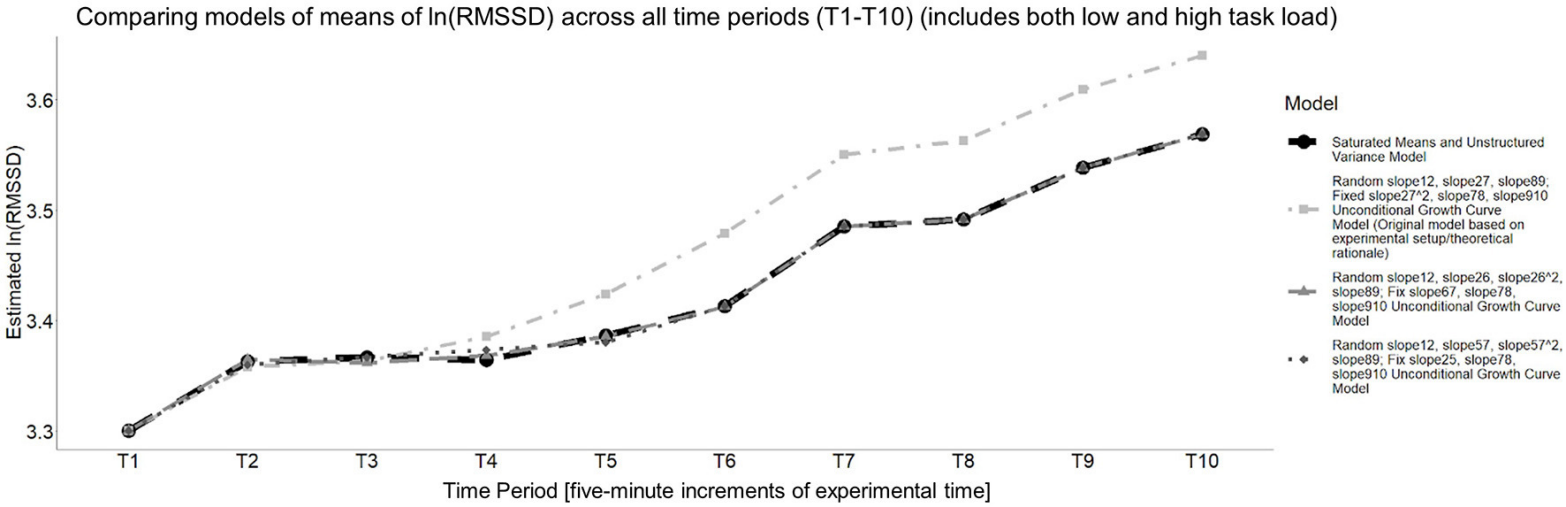
Estimated means of each unconditional growth curve model that was compared to the saturated means, unstructured variance model for cardiac vagal tone (i.e., the natural log of RMSSD). All models estimate an increase in cardiac vagal tone, on average, but the rate and timing at which it increases differs.

Figure 6 presents the model of the means for these two models and how they compare to the saturated means, unstructured variance model.

To determine the final unconditional growth curve model, BIC values were compared, which required refitting these two models with maximum likelihood estimation. The unconditional growth curve model that had a random time slope for T1–T2, a random quadratic time slope for T5–T7, a random time slope T8–T9, a fixed time slope T2–T5, a fixed time slope T7–T8, a fixed time slope for T9–T10 had the smaller BIC (BIC = −116.751) compared to the other, aforementioned model (BIC = −112.221), so it was designated as the final unconditional growth curve model, with its summary information is provided in Table 3.

**Table 3:** Parameter estimates of the best fitting growth curve model (random T1–T2 time slope, random quadratic T5–T7 time slope, random T8–T9 time slope, fixed T2–T5, T7–T8, and T9–T10 time slope).

**Parameter**	**Parameter estimate (SE)**
Baseline cardiac vagal tone (β_0*i*_)	3.301^***^ (0.082)
T1–T2 time slope (β_1*i*_)	0.060 (0.056)
T2–T5 time slope (β_2_)	0.007 (0.008)
Instantaneous linear T5–T7 time slope (β_3*i*_)	0.014 (0.072)
T5–T7 quadratic time slope (β_4*i*_)	0.019 (0.031)
T7–T8 time slope (β_5_)	0.006 (0.025)
T8–T9 time slope (β_6*i*_)	0.047 (0.036)
T9–T10 time slope (β_7_)	0.031 (0.025)
Baseline cardiac vagal tone variance ($\tau_{U_{0}}^{2}$)	0.19692
T1–T2 time slope variance	0.07898
Instantaneous linear T5–T7 time slope variance	0.10142
Quadratic T5–T7 time slope variance	0.01532
T8–T9 time slope variance	0.01966
Residual variance ($\sigma_{e}^{2}$)	0.01003

^***^ *p* < 0.001, ^**^ *p* < 0.01, ^*^ *p* < 0.05.

**Figure 7:**
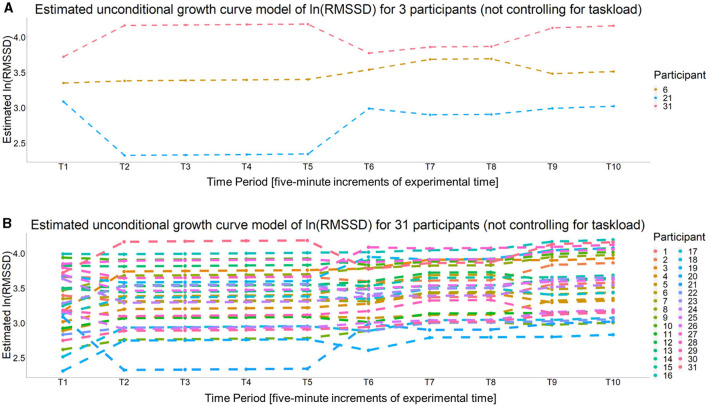
The estimated unconditional growth curves for **(A)** three participants whose estimated unconditional growth curve model are particularly different than **(B)** all participants.

Per Wald *t*-tests, none of the fixed time slope estimates were significant (all *p* > 0.05). The interpretation of this finding depends on whether the specific time slope was a significant random effect or not. For the fixed time slopes (i.e., the slopes between, T2–T5, T7–T8, and T9–T10) the non-significant fixed effect suggests a lack of evidence that cardiac vagal tone changed significantly over these time periods. However, for the four time slopes that were significant random effects (i.e., the time slope between T1–T2) the quadratic and linear time slope between T5–T7, and the time slope between T8–T9, the non-significant fixed effect estimates could be due to the fact that they were significant random effects. Recall, a significant random effect means the variability associated with the fixed effect estimate is significantly >0. Given the equation for the Wald test statistic (see Hoffman, 2015, p. 36), the lack of significance for the fixed effect may be due to the fact that the variance is (relatively) large. Also, given the fixed effect estimate is based on the “average participant,” if the parameter estimates for each individual is distributed above and below zero rather equally, then the fixed effect estimate will converge to or near zero, which is what a Wald test is assessing (i.e., the probability the estimate is significantly different than zero given its distribution). Regardless, a significant random effect suggests a single estimate for all individuals may not accurately capture the trend for each individual (i.e., there is evidence of individual differences for the given parameter estimate). This can be assessed by evaluating the 95% confidence interval (CI) for each significant random effect. First, the 95% CI for the intercept was (2.431, 4.170), which means the 95% CI for baseline RMSSD was (11.368, 64.740) ms. The 95% CI for the four random time slopes were (−0.491, 0.610) for the T1–T2 slope, (−0.611, 0.638) for the linear T5–T7 slope (−0.223, 0.262) for the quadratic T5–T7 slope, and (−0.228, 0.322) for the T8–T9 slope. These CIs show how cardiac vagal tone trends during these time periods varied greatly across participants, as all the CIs suggest some participants' cardiac vagal tone was estimated to increase while for others it was estimated to decrease during the same time period(s). Figure 7A shows the unconditional growth curve model for three participants whose estimated trajectories greatly varied and Figure 7B shows this estimate for all of the 31 participants.

#### Conditional growth curve model

In order to understand how the unconditional growth curve model related to performance and task load, these measures were evaluated as time-invariant predictors in the model. A time-invariant predictor is a measure of the individual that is not expected to change over time and/or is only reliably measured once throughout an experiment (Hoffman, 2015, p. 282). The latter served as the present rationale. Of specific interest was to assess if these measures could account for the variability estimated for the random time slopes in the unconditional growth curve model of cardiac vagal tone. This consisted of adding these measures as (mean-centered) time-invariant predictors to the intercept and specific time slopes and assessing if they accounted for significantly more variance than the unconditional growth curve model, as quantified by a change in Total *R*^2^. The significance of this change was assessed with a multivariate Wald test where degrees of freedom were corrected with Satterthwaite's method (α = 0.10; Mathieu et al., 2012; Gries, 2021; Voeten, 2021).

**Figure 8:**
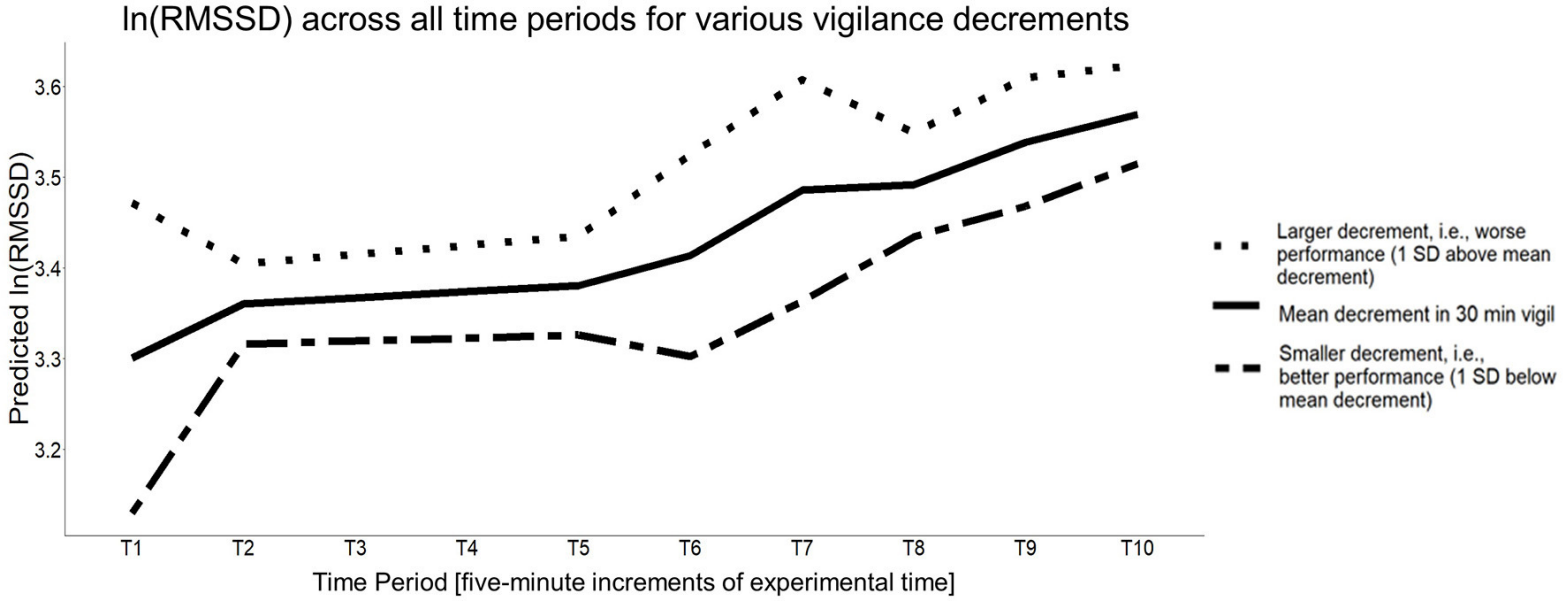
The estimated conditional growth curve for the average vigilance decrement observed, as well as 1 standard deviation above and below it, respectively.

To understand if any of the trends in cardiac vagal tone were moderated by the magnitude of the vigilance decrement (i.e., the change in performance from Block 1 to Block 3), we added Δ*A* as a time-invariant predictor to the intercept and all the piecewise time slopes in the unconditional growth curve model established above [Δ*A* from Block 1 to Block 3; *M* = 0.024, *SD* = 0.043, range: (−0.0466, 0.174)]. A multivariate Wald test found a significant omnibus effect, *F*_(8, 51.195)_ = 2.319, *p* = 0.033, specifically, the magnitude^1^ of the vigilance decrement significantly moderated the intercept (i.e., baseline cardiac vagal tone; *p* = 0.037), the time slope between T1–T2 (*p* = 0.023), and the time slope between T7–T8 (*p* = 0.012). There was no evidence that Δ*A* significantly moderated any other time slopes (*p* > 0.10). For a vigilance decrement that was one standard deviation worse than average, baseline cardiac vagal tone (i.e., its value at T1) was larger by 0.171, the time slope from T1–T2 decreased more by 1.266, and the slope from T7–T8 decreased more by 0.649. The Total *R*^2^ for this model was 0.081, which was more than double that of the unconditional growth curve model (total *R*^2^ of unconditional growth curve model = 0.039). Table 4 has this model's parameter estimates and Figure 8 illustrates how different vigilance decrements moderated cardiac vagal tone over the course of the entire experiment for those with above average decrement (worse performance), below average decrement (better than average), and mean decrement.

**Table 4:** Parameter estimates of the conditional growth curve model that has the change in *A* as a time-invariant predictor.

**Parameter (by level)**	**Parameter estimate (SE)**
Baseline cardiac vagal tone (β_0*i*_)	
Baseline cardiac vagal tone (γ_00_)	3.301^***^ (0.077)
Δ*A* (γ_01_)	4.012^*^ (1.837)
T1–T2 time slope (β_1*i*_)	
T1–T2 slope (γ_10_)	0.060^*^ (0.052)
T1–T2 slope × Δ*A* (γ_11_)	−2.971^*^ (1.240)
T2–T5 time slope (β_2_)	
T2–T5 slope (γ_20_)	0.007 (0.008)
T2–T5 slope × Δ*A* (γ_21_)	0.079 (0.189)
Instantaneous T5–T7 time slope (β_3*i*_)	
T5–T7 slope (γ_30_)	0.013 (0.070)
T5–T7 slope × Δ*A* (γ_31_)	1.871 (1.679)
Quadratic T5–T7 time slope (β_4*i*_)	
T5–T7 slope (γ_40_)	0.019 (0.030)
T5–T7 slope × Δ*A* (γ_41_)	−0.538 (0.726)
T7–T8 time slope (β_5_)	
T7–T8 slope (γ_50_)	0.006 (0.025)
T7–T8 slope × Δ*A* (γ_51_)	−1.522^*^ (0.599)
T8–T9 time slope (β_6*i*_)	
T8–T9 slope (γ_60_)	0.047 (0.036)
T8–T9 slope × Δ*A* (γ_61_)	0.317 (0.854)
T9–T10 time slope (β_7_)	
T9–T10 slope (γ_70_)	0.031 (0.025)
T9–T10 slope × Δ*A* (γ_71_)	−0.378 (0.599)
Baseline cardiac vagal tone variance ($\tau_{U_{0}}^{2}$)	0.174
T1–T2 time slope variance	0.067
Instantaneous linear T5–T7 time slope variance	0.097
Quadratic T5–T7 time slope variance	0.015
T8–T9 time slope variance	0.020
Residual variance ($\sigma_{e}^{2}$)	0.010

^***^ *p* < 0.001, ^**^ *p* < 0.01, ^*^ *p* < 0.05.

To understand if any of the trends in cardiac vagal tone were moderated by the change in the speed of correct responses throughout the vigilance task, we added the change in median response time of hits from Block 1 to Block 3 as a time-invariant predictor to the intercept and all of the piecewise time slope in the unconditional growth curve model established above [ΔHitRTMed between Block 1 and Block 3^2^; *M* = −49.600 ms, *SD* = 113.366 ms, range: (−441 ms, 193 ms)]. A multivariate Wald test found no significant omnibus effect, *F*_(8, 51.451)_ = 0.899, *p* = 0.524, suggesting this decrement (i.e., slowed responding) during the vigilance task did not moderate the growth curve model.

To understand if the trends in cardiac vagal tone immediately before, during, and after the break were moderated by how much the vigilance decrement was mitigated by the break, Δ*A* between Block 3 and Block 4 was added as a time-invariant predictor to the intercept and the T5–T7, T7–T8, T8–T9, and T9–10 time slopes in the unconditional growth curve model established above [Δ*A* between Block 3 and Block 4^3^; *M* = −0.001, *SD* = 0.035, range: (−0.071, 0.092)]. A multivariate Wald test found no significant omnibus effect, *F*_(6, 52.515)_ = 1.522, *p* = 0.189, suggesting the change in cardiac vagal tone that happens between Block 3, the break, and Block 4 was not moderated by how much the vigilance decrement was mitigated between Block 3 and Block 4.

To understand if the trends in cardiac vagal tone immediately before, during, and after the break were moderated by how much the vigilance decrement was mitigated by the break, the change in median response time of hits between Block 3 and Block 4 was added as a time-invariant predictor to the intercept and the T5–T7, T7–T8, T8–T9, and T9–10 time slopes in the unconditional growth curve model established above [ΔHitRTMed between Block 3 and Block 4^4^; *M* = 25.100 ms, *SD* = 107.130 ms, range: (−173.5,302)]. A multivariate Wald test found no significant omnibus effect, *F*_(6, 45.896)_ = 1.23, *p* = 0.309, suggesting the change in cardiac vagal tone that happens in Block 3, the break, and Block 4 was not moderated by the change in hit median response time between Block 3 and Block 4.

Finally, to understand if task load moderated any of the trends in cardiac vagal tone, Task Load Condition was added as a time-invariant predictor to the intercept and to each piecewise time slope in the unconditional growth curve model established above. A multivariate Wald test found no significant omnibus effect of Task Load Condition, *F*_(8, 51.112)_ = 1.563, *p* = 0.161, further supporting our initial assumption that cardiac vagal tone trends were not moderated by task load.

## Discussion

The goal of the present work was to explore the changes in cardiac vagal tone over the entire span of a vigilance task and its corresponding relation to performance, while simultaneously studying the dependency they both have on the individual participant. Our goals were exploratory in nature given the dearth of research that studies cardiac vagal tone during a vigilance task. In summary, our current results suggest cardiac vagal tone during vigilance is non-monotonic, individualistic, and associated with vigilance task performance. However, it seems cardiac vagal tone does not fully explain performance nor its competing theories. Although exploratory in nature, these results benefit the knowledge base as they start to better inform the applicability of existing theory and potential ways to revise it. They also start to inform how to develop individualized, psychophysiological-based countermeasures to the vigilance decrement in an operational setting. We discuss our present findings in detail with a focus on the interpretation of the growth curve modeling results and their subsequent impact on theory and application.

### Performance

To confirm our manipulation produced a vigilance decrement, we analyzed average performance across the experimental blocks. We found evidence of a vigilance decrement in both measures of sensitivity and decision speed during the vigilance task. This suggests our experimental design replicated the typical, temporal-based decline in performance observed during a vigilance task. An examination of performance for each individual from Block 1 to Block 3 showed a number of participants (one third to two thirds of the sample) improved in performance rather than worsened. Similarly, comparing performance from Block 3 to Block 4 showed about half to two-thirds of participants improved in sensitivity or latency from the break. Importantly, these findings were not captured when comparing. These types of variations in performance have been previously observed (Aue et al., 2009; Smith et al., 2019). Albeit descriptive and *ad hoc*, this is evidence that aggregated trends may not accurately represent all, or in some cases, the majority of participants. This further substantiates our claim that performance during a vigilance task should be analyzed at an individual level and this should be the focus of future work. Presently, these variations in performance strengthen our conditional growth curve modeling analysis because having diverse values for a given predictor increases our understanding on how a vigilance decrement moderates a growth curve model.

### Unconditional growth curve modeling

As a reminder, the unconditional growth curve model in this work estimated how cardiac vagal tone trends over the course of a vigilance task, a break, its resumption of and its corresponding dependency on the individual participant—all of which were our overarching research goals. Upon reviewing the magnitude, direction, timing, and associated variability of the time slope estimates in the final unconditional growth curve model, it is clear cardiac vagal tone trends differ depending on (1) the person, (2) the immediate task demands (i.e., time period within the vigilance task and the break), (3) time-on-task, and (4) the person's vigilance decrement.

#### Immediate task demands

The statistical significance of a random intercept suggests baseline cardiac vagal tone is unique to each person (i.e., an individual difference). This was not a surprising result (Magnon et al., 2022), but for the remaining random time slopes, this is novel, insightful, and informative for understanding the way in which cardiac vagal tone trends during a vigilance task, break, and post-break vigilance task. In short, the way in which cardiac vagal tone trends during certain time periods depends on the participant and does not change significantly otherwise. For example, the significant random time slope that spans from baseline to the first 5 min of the vigilance task and the significant random time slope that spans from the break to the first 5 min of the post-break vigilance task were followed by non-significant fixed time slopes. In other words, an individual's cognitive and emotional regulation within the first 5 min of the task remained for some portion of the task. However, this trend did not persist for its entire duration. Cardiac vagal tone in the latter parts of the vigilance task were dependent upon the individual and did not converge at the group level until the onset of the break. This was supported by the finding of a random quadratic time slope for the final 10 min of the vigilance task. We discuss this finding further in the next section (Time-on-task) to better understand the effect time-on-task had on cardiac vagal tone.

To understand the effect of the break, we examine the non-significant fixed time slope between the last 5 min of Block 3 and the break and the random time slope between the break to the first 5 min of Block 4. The former suggests there is no evidence that cardiac vagal tone changed during the break. Previous research found a performance benefit from a break and suggests it was due to cognitive resource recovery (McCormack, 1958; Bergum and Lehr, 1962; Ariga and Lleras, 2011; Helton and Russell, 2012, 2015, 2017; Ross et al., 2014; Ralph et al., 2017). If a break allowed cognitive resources to replenish, we would expect cardiac vagal tone to increase, indicating an increase in parasympathetic nervous system activity and thus, a recovery in cognitive and emotional resource regulation. Therefore, at first glance, these results do not align with previous research. However, the time slope between the break to the first 5 min of Block 4 was a random effect, indicating it differed across individuals (i.e., cardiac vagal tone increased, decreased, or stayed the same depending on the individual participant). This finding suggests that the effect of the break depends on the individual. In addition, the parasympathetic activity of interest may happen on a time scale that is shorter than 5 min and therefore was undetected in this analysis and/or only happens for some individuals and not all. The latter is supported by our descriptive, *ad hoc* investigation of performance, as we found $\frac{1}{2}$ to 1/3 of participants potentially do not benefit from the break as their performance from Block 3 to Block 4 did not improve. Future work should further explore this by either analyzing indices of cardiac vagal tone on shorter time scales (e.g., some suggest HRV recordings of 1 min are sufficient; Laborde et al., 2017) and/or increase the duration of the break and study longitudinal cardiac vagal tone trends during this period specifically.

#### Time-on-task

Cardiac vagal tone did not monotonically change with time-on-task, rather it changed at particular time points in the experiment. Some of these changes coincided with the different phases of the experiment (Blocks 1–3, the break, Block 4), whereas some did not. More interestingly, the way in which cardiac vagal tone changed was dependent on the participant. For example, the best fitting growth curve model had two separate piecewise time slopes during the vigilance task, which means a singular slope estimate for the entire vigilance task (i.e., Blocks 1–3) was not sufficient in characterizing how cardiac vagal tone trended over time. Specifically, cardiac vagal tone during the first 20 min of the vigilance task was estimated by one, *fixed* linear piecewise slope, suggesting each participant's cardiac vagal tone trended the same for this length of time. However, for the last 10 min of the vigilance task a separate, *random* quadratic piecewise slope was necessary. Importantly, this specific piecewise slope was unique to the individual, meaning that the change in cardiac vagal tone differed in direction and/or steepness for each participant. This finding aligns with previous work that also found non-monotonic and inconsistent changes in psychophysiology during a vigilance task (Smith et al., 2019). Although it does not further disentangle theories of task engagement or disengagement, it does suggest that the decrement depends on the individual. In some cases, it could be that some individuals might be able to maintain engagement for the entire experiment, whereas others may disengage or have increasing task-unrelated thoughts, as explained by resource-control and/or mind-wandering theories. Alternatively, some individuals may have the cognitive resources to maintain performance whereas others do not, as explained by resource theory. Our current analysis cannot currently specify this, but future work should investigate further with other experimental manipulations and time-based, person-level measures and analyses as it seems cognitive and emotional regulation are essential to completing a vigilance task.

### Conditional growth curve modeling

#### Performance and task load

To this point, we have discussed how cardiac vagal tone trends across the vigilance task, but we have yet to discuss how these trends are associated with its performance. The conditional growth curve model that included a vigilance decrement (specifically Δ*A*) between Block 1 and Block 3 was a significant moderator on cardiac vagal tone trends and its effect was noteworthy as the amount of variance accounted for doubled. It specifically indicated that the vigilance decrement was associated with three aspects of the growth curve: (1) baseline cardiac vagal tone, (2) the change from baseline to the first 5 min of the vigilance task and, unexpectedly, (3) the change from the last 5 min of the vigilance task to the break. The vigilance decrement was not a significant moderator of cardiac vagal tone during the middle 20 min of vigilance task, its change after the break, nor during the post-break vigilance task.

Overall, the conditional growth curve model suggested those with a **larger** than average vigilance decrement (i.e., worse performance decline) had a **higher** cardiac vagal tone at baseline, a **steeper decrease** in cardiac vagal tone when starting the vigilance task, (i.e., starting Block 1) as well as a **steeper decrease** in cardiac vagal tone from the last 5 min of the vigilance task (i.e., the end of Block 3) to starting the break, with the changes in cardiac vagal tone being reversed with a smaller than average vigilance decrement. Evidently, the vigilance decrement is associated not only with baseline cardiac vagal tone, but also how much it changes once starting and stopping a vigilance task. Although all novel, it is particularly interesting that higher baseline cardiac vagal tone, thought to indicate better cognitive and emotional regulation (Porges, 2003; Thayer et al., 2012), was associated with a larger vigilance decrement, however, Laborde et al. (2017) point out that the relationship between those with high and low vagal tone may be different depending on when HRV was measured (e.g., at rest or during the task). Matthews et al. (2010) found pre-task engagement predicted cerebral blood flow response during the vigilance task. They suggested that pre-task engagement represented “a state of readiness for resource mobilization.” Recent research similarly found children with higher working memory capacities showed higher initial cardiac vagal tone followed by greater withdrawal compared to those with lower working memory capacities who demonstrated lower initial cardiac vagal tone and subsequent augmentation in cardiac vagal tone. These authors made a comparable argument to that of Matthews et al. (2010) suggesting the trends in cardiac vagal tone better prepared the high working memory group for cognitive tasks and allowed improved maintenance of arousal during task engagement (Obradović and Finch, 2017). These findings align with polyvagal theory (Porges, 2003) and neurovisceral integration theory (Thayer et al., 2009). However, our findings of larger baseline cardiac vagal tone being associated with poorer vigilance performance may be due to differences in our experimental design. Unlike Matthews et al. (2010), we measured changes in cardiac vagal tone instead of changes in cerebral blood flow, which may capture related, but different cognitive processes. Additionally, Obradović and Finch (2017) assessed changes in cardiac vagal tone in children across eight, 30-s epochs, which was much shorter than our vigilance task paradigm. The differences in population (children vs. adults) and task duration (4 vs. 45.8 min) may account for differences in our findings. This further emphasizes the need for future research to better understand the relationship between cardiac vagal tone, cognitive and emotional regulation, and vigilance performance.

The vigilance decrement was not a significant moderator of cardiac vagal tone during the vigilance task, its change after the break, or during the post-break vigilance task. This is interesting because this differs from the relationship observed between cerebral blood flow and vigilance performance (Tripp and Warm, 2007; Shaw et al., 2009, 2013). Also, task load was not found to be a significant moderator of the growth curve model of cardiac vagal tone. This further supports the notion that these two psychophysiological processes may not be measuring the same construct and furthers the call for vigilance research to study measures on the individual level. Collectively, these findings support the concept that cognitive and emotional regulation during a task is a *process* and how psychophysiological measures manifest during these phases may impact behavioral outcomes. Obradović and Finch (2017) define three distinct phases of said process: an initial arousal, reactivity, and recovery phase. We specifically found evidence that a person's *initial* physiological state, physiological *reaction* to starting a vigilance task, and *recovery* from it is more indicative of how they are going to perform than the psychophysiology *during* the task or its respective task load. Our phases were defined *post hoc*, but our results suggest this cycle may be happening for some individuals during the vigilance task. Undoubtedly, further work is needed to replicate these results, but regardless, it is evident that cardiac vagal tone is a highly individualized process and is partially indicative of how a person will perform in a vigilance task. Not only will this further the psychophysiological knowledgebase, but also help to address the previously discussed shortcomings in vigilance theory.

### Impact current results have on theory and applications

Our work does not disentangle existing theories of vigilance, as our goal was to explore a potential relationship between individual differences in cardiac vagal tone trends and its association with vigilance task performance. The current findings suggest cardiac vagal tone and its changes across different time periods of a vigilance task are informative on a person's vigilance task performance. In general, this finding further supports the notion that physiological responses during a cognitive task are highly individualized (Obradović and Finch, 2017). We argue this work provides evidence to revise vigilance theory to place a stronger emphasis on the role of individual differences and calls for analysis methods to analyze temporal data properly. Simultaneously, this work demonstrates how aggregated analyses obscure the uniqueness of each individual's functioning and changes in performance over time. Individual differences in vigilance tasks have been previously observed, somewhat consistently, but its rationale has not been well-explained. This work may serve as a catalyst to better understand why individual differences exist—they are due to the highly individualistic nature of cognitive and emotional regulation.

On a more applicable level, this suggests that measuring cardiac vagal tone prior to starting any vigilance task may be an indicator of how someone is going to perform in that environment. This is in line with the individualistic nature of vigilance task performance; however, our findings further suggest this may serve as a potential way to screen and predict how people will perform in vigilance tasks. Further, understanding how cardiac vagal tone *changes* upon starting a vigilance task may also be indicative of the severity (let alone presence of) a vigilance decrement, which is arguably easier to apply in real-life settings than determining thresholds of baseline cardiac vagal tone, as the former only requires detecting the directional change of cardiac vagal tone once starting a vigilance task. For example, it may be advantageous to stop a person from completing a vigilance task if their cardiac vagal tone declines rapidly at the onset of the task, which can be done administratively (e.g., job rotations) and/or with adaptive technology (e.g., decision aids, automation, etc.).

Finally, although there were no performance differences due to the break, on average, (i.e., performance was not better between Block 3 and Block 4), the break may have functionality in predicting the magnitude of the vigilance decrement, as those with a larger than average vigilance decrement before the break were predicted to have a steeper decline in cardiac vagal tone upon starting the break. It may be that worse performance on the task was due to greater decline in cognitive and emotion regulation, which may impact the ability to complete the vigilance task. Future research needs to better understand the vigilance decrement-cardiac vagal tone relationship, specifically if cardiac vagal tone is reflecting cognitive regulation, emotional regulation, or some combination of both during vigilance performance. Additionally, a break may not be beneficial if someone is not using it to actively regulate the cognitive and/or emotional resources that are necessary to complete a vigilance task. Further supporting this notion is the random time slope between the end of the break and starting Block 4. This random time slope could be capturing the different approaches participants took during the break, resulting in their cardiac vagal tone trajectory to differ upon returning to a vigilance task. This information would not have been known without growth curve modeling because pairwise comparisons would not have captured the dynamic, individual nature of these changes. In fact, it may have reported that, on average, there was no change in cardiac vagal tone upon resuming a vigilance task from a break—which has very different implications to what was observed with growth curve modeling.

### Limitations and future work

It is interesting that the vigilance decrement did not significantly moderate the random time slope in Block 3 nor the random time slope from ending the break to starting Block 4. There are several potential reasons for this: (1) the performance measures that were used in this investigation (*A* and median response time of hits) are not the best indicators of these respective changes in cardiac vagal tone; (2) the time scale these measures were calculated on was not resolute enough, as they were calculated on a block basis not a time period basis; and/or, (3) it could be the variability in these random time slopes is better moderated by measures of engagement, fatigue, and/or other individual differences measures previously mentioned in the introduction. Nevertheless, the vigilance decrement was a significant moderator of how cardiac vagal tone changed once starting and stopping a vigilance task, suggesting the latter is the most probable explanation. This further supports the notion that psychophysiological measures need to be modeled over time given they are process-based measures. Future research should explore if more granular analyses are warranted to understand if vagal tone changes occur more frequently than every 5 min in line with research finding changes in other measures of autonomic functioning at smaller time scales (e.g., Golland et al., 2014; Pasquini et al., 2022). Even though 5 min is thought to be the minimum amount of time necessary for an accurate HRV measure, other work finds reliable estimates for shorter time periods (Laborde et al., 2017; Tung et al., 2021). Future work should continue to explore the relationship between baseline cardiac vagal tone and the vigilance decrement, especially in the context of theories relating pre-task engagement to the decrement (see Matthews et al., 2010). An additional area of future work concerns the “change point” in cardiac vagal tone during a vigilance task. Of interest is to better understand if such a point is the same or also differs per the individual. For example, latent change point modeling empirically derives when another piecewise slope should be created for a given individual. This analysis requires significantly more participants (Hoffman, 2015, p. 230), but previous vigilance research that has somewhat explored this for cerebral blood flow velocity during a vigilance task and found interesting results (Tolston et al., 2020), so it may be worthwhile. Last, vigilance theories may be further informed and disentangled by directly studying different manipulations of a vigilance task with individual differences and longitudinal modeling methods. Our current experiment is not able to directly flesh out any of the various theories, but it clearly demonstrated the effect of studying psychophysiology on an individual and longitudinal basis. Overall, future work should continue to include other person-level predictors in growth curve models to better understand if variability is an individual or sub-group difference.

## Conclusion

We set out to explore the dynamic relationship between trends in cardiac vagal tone and performance in a vigilance task. Though the nature of this work was exploratory and is not without limitations, it is clear that implementing longitudinal analyses, like growth curve modeling, to study cardiac vagal tone trends during a vigilance task is informative. Our findings further suggest that cardiac vagal tone and its relationship to performance in cognitive tasks is not unitary and is unlikely to be accurately characterized by a single averaged measure for each person. Specifically, the unconditional growth curve modeling results illustrated several ways in which individual differences affected cardiac vagal tone. Results indicated that cardiac vagal tone at rest and how it changes once starting and stopping a vigilance task may be more indicative of performance than how it trends during the task. Cardiac vagal tone should continue to be explored as a process-based psychophysiological measure in both basic and applied vigilance research efforts to better understand how the vigilance decrement manifests over time, its dependency on individual differences, and how it is mitigated against the vigilance decrement.

## Data availability statement

The data and supporting materials can be found here: https://osf.io/r6uma/.

## Ethics statement

The studies involving humans were approved by Department of Psychological Sciences at Texas Tech University. The studies were conducted in accordance with the local legislation and institutional requirements. The participants provided their written informed consent to participate in this study.

## Author contributions

SM: conceptualization, formal analysis, investigation, writing—original draft, and visualizations. BN: conceptualization, methodology, data collection, writing—original draft, project administration, and visualization. NB: funding acquisition, conceptualization, formal analysis, investigation, and writing—original draft. KS: methodology and writing—original draft, visualization. EG: conceptualization, writing—review and editing, and supervision. MK: writing—review and editing. JC: project administration, supervision, and resources. All authors contributed to the article and approved the submitted version.

## Footnotes

^1^For this measure, **positive** values indicate a **decrement** in performance given the formula for this measure was: Δ*A* = *A* for Block 1 – *A* for Block 3. A performance decrement was observed for 2/3 of participants.

^2^For this measure, **negative** values indicate a **decrement** in response time given the formula for this measure was: ΔHitRTMed = Median response time for Block 1 – Median response time for Block 3. A decrement in median response time was found for 1/2 of participants.

^3^For this measure, **positive** values indicate a **decrement** in sensitivity performance given the formula for this measure was: Δ*A* = *A* for Block 3 – *A* for Block 4. A performance decrement was observed for about $\frac{1}{2}$ of the participants.

^4^For this measure, **negative** values indicate a **decrement** in response time performance given the formula for this measure was: ΔHitRTMed = Median response time for Block 3 – Median response time for Block 4. A decrement in median response time was found for about 2/3 of participants.
