# Supplementary material for: An investigation of cardiac vagal tone over time and its relation to vigilance performance: a growth curve modeling approach
Source: Front Neuroergon. 2023 Dec 11;4:1244658. doi: 10.3389/fnrgo.2023.1244658 (PMC10790917; doi:10.3389/fnrgo.2023.1244658)
Supplement: Supplementary file 1 [file Presentation_1.zip › FrontiersHRVGCMs_RMrkdn_PubRelevCode_FeedbackEdits_TitledChunks_DistroAStatement_09-06-2023.html]

Data Analysis for ‘An investigation of cardiac vagal tone over time and its relation to vigilance performance: A growth curve modeling approach’


# Data Analysis for ‘An investigation of cardiac vagal tone over time and its relation to vigilance performance: A growth curve modeling approach’

#### Shannon P. D. McGarry

U.S. Naval Research Laboratory, Information Technology Division,
Washington, DC, United States  

#### Brittany N. Neilson

Naval Aerospace Medical Institute, Operational Psychology Department,
Pensacola, FL, United StatesTexas Tech University, Department of
Psychological Sciences, Lubbock, TX, United
States  

#### Noelle L. Brown

U.S. Naval Research Laboratory, Information Technology Division,
Washington, DC, United States  

#### Kaylin D. Strong

Strategic Analysis, Inc., Arlington, VA, United
States  

#### Eric T. Greenlee

Texas Tech University, Department of Psychological Sciences, Lubbock,
TX, United States  

#### Martina I. Klein

Texas Tech University, Department of Psychological Sciences, Lubbock,
TX, United States  

#### Joseph T. Coyne

U.S. Naval Research Laboratory, Information Technology Division,
Washington, DC, United States  

#### Last update: 06 September 2023

```
knitr::opts_chunk$set(warning=FALSE, message=FALSE)
```

# Introduction

Distribution statement A, Approved for public release, Distribution
unlimited

Decades of research has demonstrated a relationship between
psychophysiological measures, specifically cardiac functions, and
cognitive performance. Specifically, regulation of the cardiac system
under parasympathetic control is commonly referred to as cardiac vagal
tone and is associated with the regulation of cognitive and
socioemotional states. The goal of the current study was to capture the
dynamic relationship between cardiac vagal tone and performance in a
vigilance task. The current research seeks to contribute to our
understanding of vigilance by understanding how trends in cardiac vagal
tone are informative of individual differences in vigilance
performance.

# Preparation

Install and load packages.

```
if (!require("car")) install.packages("car")
library(car)
if (!require("DescTools")) install.packages("DescTools")
library(DescTools)
if (!require("devtools")) install.packages("devtools")
library(devtools)
if (!require("plyr")) install.packages("plyr")
library(plyr)
if (!require("dfoptim")) install.packages("dfoptim")
library(dfoptim)
if (!require("dplyr")) install.packages("dplyr")
library(dplyr)
if (!require("effectsize")) install.packages("effectsize")
library(effectsize)
if (!require("effsize")) install.packages("effsize")
```

```
## Warning: package 'effsize' was built under R version 4.2.3
```

```
library(effsize)
if (!require("emmeans")) install.packages("emmeans")
library(emmeans)
if (!require("ggeffects")) install.packages("ggeffects")
```

```
## Warning: package 'ggeffects' was built under R version 4.2.3
```

```
library(ggeffects)
if (!require("ggplot2")) install.packages("ggplot2")
library(ggplot2)
if (!require("ggrepel")) install.packages("ggrepel")
```

```
## Warning: package 'ggrepel' was built under R version 4.2.3
```

```
library(ggrepel)
if (!require("Hmisc")) install.packages("Hmisc")
library(Hmisc)
if (!require("ISLR")) install.packages("ISLR")
```

```
## Warning: package 'ISLR' was built under R version 4.2.3
```

```
library(ISLR)
if (!require("lme4")) install.packages("lme4")
library(lme4)
if (!require("lmerTest")) install.packages("lmerTest")
```

```
## Warning: package 'lmerTest' was built under R version 4.2.3
```

```
library(lmerTest)
if (!require("mosaic")) install.packages("mosaic")
```

```
## Warning: package 'mosaic' was built under R version 4.2.3
```

```
library(mosaic)
if (!require("multcomp")) install.packages("multcomp")
```

```
## Warning: package 'multcomp' was built under R version 4.2.3
```

```
## Warning: package 'TH.data' was built under R version 4.2.3
```

```
library(multcomp)
if (!require("nlme")) install.packages("nlme")
library(nlme)
if (!require("optimx")) install.packages("optimx")
```

```
## Warning: package 'optimx' was built under R version 4.2.3
```

```
library(optimx)
if (!require("pbkrtest")) install.packages("pbkrtest")
library(pbkrtest)
if (!require("performance")) install.packages("performance")
library(performance)
if (!require("purrr")) install.packages("purrr")
library(purrr)
if (!require("psych")) install.packages("psych")
library(psych)
if (!require("pwr")) install.packages("pwr")
```

```
## Warning: package 'pwr' was built under R version 4.2.3
```

```
library(pwr)
if (!require("rstatix")) install.packages("rstatix")
```

```
## Warning: package 'rstatix' was built under R version 4.2.3
```

```
library(rstatix)
if (!require("sciplot")) install.packages("sciplot")
library(sciplot)
if (!require("sjstats")) install.packages("sjstats")
library(sjstats)
if (!require("stats")) install.packages("stats")
library(stats)
if (!require("stringr")) install.packages("stringr")
library(stringr)
if (!require("TeachingDemos")) install.packages("TeachingDemos")
```

```
## Warning: package 'TeachingDemos' was built under R version 4.2.3
```

```
library(TeachingDemos)
if (!require("tidyr")) install.packages("tidyr")
library(tidyr)
if (!require("tidyverse")) install.packages("tidyverse")
library(tidyverse)
```

Create citation function for later use.

```
citations <- function(includeURL = TRUE, includeRStudio = TRUE) {
  if(includeRStudio == TRUE) {
    ref.rstudio <- RStudio.Version()$citation
    if(includeURL == FALSE) {
      ref.rstudio$url <- NULL;
    }
    print(ref.rstudio, style = 'text')
    cat('\n')
  }
  
  cit.list <- c('base', names(sessionInfo()$otherPkgs))
  for(i in 1:length(cit.list)) {
    ref <- citation(cit.list[i])
    if(includeURL == FALSE) {
      ref$url <- NULL;
    }
    print(ref, style = 'text')
    cat('\n')
  }
}
```

Load HRV data and other data objects that take a long time to compute
but are important for the analysis

```
#change which RData file is loaded depending on what machine is used to knit the file
#load("C:/Users/mcgarry/OneDrive - US Navy-flankspeed/Projects/CANARE/HRV_BrittDiss/Output/DataForRedRMrkdwnGCMs_2023-08-08.RData")
load("C:/Users/NRL/OneDrive - US Navy-flankspeed/Projects/CANARE/HRV_BrittDiss/Output/DataForRedRMrkdwnGCMs_2023-08-08.RData")

length(colnames(HRV_TTU_RawData))==457 #This makes sure the correct raw dataset was loaded. 457 is the expected # of columns
```

You have the wrong data if the above returns as FALSE…review the raw
data that you are loading to assure it is the correct raw HRV data

Modify the data frame output from wide to long format.

```
HRV_TTU_RawData_Long<-gather(data = HRV_TTU_RawData, key="T", value="Value", 7:457) #extend data file so the metric type is in a single column and the corresponding value is in the very next column
HRV_TTU_RawData_Long<-separate(data = HRV_TTU_RawData_Long, col = T, into=c("Timepoint","Metric","Metric2","Metric3","Metric4","Metric5"), sep = "\\_") #this line of code separates out every item separated by a _ in to a new column. NOTE: A warning is produced when this line of code runs bc not all metrics have 6 words associated with them, but we have to design for the latter
```

```
## Warning: Expected 6 pieces. Missing pieces filled with `NA` in 14304 rows [1, 2,
## 3, 4, 5, 6, 7, 8, 9, 10, 11, 12, 13, 14, 15, 16, 17, 18, 19, 20, ...].
```

```
HRV_TTU_RawData_Long<-unite(HRV_TTU_RawData_Long, Metric, Metric:Metric5, sep = "_") #combines all the words associated with the metric except the time period label - aka we have correctly created the time period column
HRV_TTU_RawData_Long<-separate(data = HRV_TTU_RawData_Long, col = Metric, into = c("Metric"), sep = "\\_NA") #gets rid of any NAs that might exist in the metric name as some metric names are shorter than others and this code works for the longest metric names and adds in NA when the metric name is shorter than the longest metric name in the file. NOTE: A warning is produced when this line of code runs bc of what was just mentioned
```

```
## Warning: Expected 1 pieces. Additional pieces discarded in 14304 rows [1, 2, 3,
## 4, 5, 6, 7, 8, 9, 10, 11, 12, 13, 14, 15, 16, 17, 18, 19, 20, ...].
```

Isolate T8, i.e., the break, from all other time periods in order to
clean up the data frame.

It is necessary to isolate T8 (i.e., the break) because the metrics
are calculated twice for this time period…they are calculated for the
first and last 5 minutes of the 5.8 min break. However, we want each
time period to be 5 and only 5 minutes in duration as this an assumption
of growth curve models.

```
HRV_TTU_RawData_Long_NoT8<-subset.data.frame(HRV_TTU_RawData_Long, HRV_TTU_RawData_Long$Timepoint!="T8")
HRV_TTU_RawData_Long_NoT8<-spread(data = HRV_TTU_RawData_Long_NoT8, key = "Metric", value = "Value")
HRV_TTU_RawData_Long_T8Only<-subset.data.frame(HRV_TTU_RawData_Long, HRV_TTU_RawData_Long$Timepoint=="T8")
HRV_TTU_RawData_Long_T8Only<-separate(data = HRV_TTU_RawData_Long_T8Only, col = Metric , into = c("Timeperiod","Metric","Metric2","Metric3","Metric4"), sep = "\\_")
```

```
## Warning: Expected 5 pieces. Missing pieces filled with `NA` in 2496 rows [1, 2,
## 3, 4, 5, 6, 7, 8, 9, 10, 11, 12, 13, 14, 15, 16, 17, 18, 19, 20, ...].
```

```
HRV_TTU_RawData_Long_T8Only<-unite(HRV_TTU_RawData_Long_T8Only, Metric, Metric:Metric4, sep = "_")
HRV_TTU_RawData_Long_T8Only<-separate(data = HRV_TTU_RawData_Long_T8Only, col = Metric, into = c("Metric"), sep = "\\_NA") #NOTE: A warning is produced when this line of code runs bc not all metrics have same # of words but we have to designed for this in the previous lines of code
```

```
## Warning: Expected 1 pieces. Additional pieces discarded in 2496 rows [1, 2, 3,
## 4, 5, 6, 7, 8, 9, 10, 11, 12, 13, 14, 15, 16, 17, 18, 19, 20, ...].
```

```
HRV_TTU_RawData_Long_T8Only<-spread(data = HRV_TTU_RawData_Long_T8Only, key = "Metric", value = "Value")
```

Because all the metrics are calculated twice for T8, i.e., the break,
we need an additional column to identify which time period each metric
was based on. A dummy column is added for all other time points in order
to rbind the two data frames together.

```
HRV_TTU_RawData_Long_NoT8["Timeperiod"]<-"full5"
HRV_TTU_RawData_Long_NoT8<-subset.data.frame(HRV_TTU_RawData_Long_NoT8,select=c(New_ID,Original_ID, TL_Condition, Pop, Break_Cond, FileName, Timepoint, Timeperiod, BeatsCorrected_Percent, HFband, HFpeak_AR_Hz,HFpeak_FFT_Hz,HFpow_AR_log,HFpow_AR_ms2, HFpow_AR_percent, HFpow_FFT_log, HFpow_FFT_ms2, HFpow_FFT_percent, LF_HF_ratio_AR, LF_HF_ratio_FFT, LFband, LFpeak_AR_Hz, LFpeak_FFT_Hz, LFpow_AR_log, LFpow_AR_ms2, LFpow_AR_percent, LFpow_FFT_log, LFpow_FFT_ms2,LFpow_FFT_percent, Max_HR_bpm, Mean_HR_bpm, Mean_RR_ms, Min_HR_bpm, Noise_Percent, PNSindex, RESP_Hz,RMSSD_ms, SD_HR_bpm, SNSindex, Stressindex, VLFband, VLFpeak_AR_Hz, VLFpeak_FFT_Hz, VLFpow_AR_log, VLFpow_AR_ms2, VLFpow_AR_percent, VLFpow_FFT_log, VLFpow_FFT_ms2, VLFpow_FFT_percent)) 

HRV_TTU_RawData_Long_AllTimepoints<-rbind(HRV_TTU_RawData_Long_NoT8,HRV_TTU_RawData_Long_T8Only)
```

Here we are organizing and tidying the data frame so that it is
easily called upon for further use.

```
HRV_TTU_RawData_Long_AllTimepoints$TL_Condition[which(HRV_TTU_RawData_Long_AllTimepoints$Original_ID=="14")]<-"L"
HRV_TTU_RawData_Long_AllTimepoints$Timepoint<-factor(HRV_TTU_RawData_Long_AllTimepoints$Timepoint, levels = c("T1","T2","T3","T4","T5","T6","T7","T8","T9","T10"))
HRV_TTU_RawData_Long_AllTimepoints$TL_Condition<-factor(HRV_TTU_RawData_Long_AllTimepoints$TL_Condition, levels = c("L","H"))
HRV_TTU_RawData_Long_AllTimepoints$Break_Cond<-factor(HRV_TTU_RawData_Long_AllTimepoints$Break_Cond, levels = c("N","U"))
HRV_TTU_RawData_Long_AllTimepoints$Timeperiod<-factor(HRV_TTU_RawData_Long_AllTimepoints$Timeperiod, levels = c("full5","first5","last5"))
HRV_TTU_RawData_Long_AllTimepoints<-HRV_TTU_RawData_Long_AllTimepoints[order(HRV_TTU_RawData_Long_AllTimepoints$New_ID,HRV_TTU_RawData_Long_AllTimepoints$Timepoint,HRV_TTU_RawData_Long_AllTimepoints$Timeperiod),]
row.names(HRV_TTU_RawData_Long_AllTimepoints)<-1:nrow(HRV_TTU_RawData_Long_AllTimepoints)
```

Organize and relabel data for enhanced interpretation.

```
HRV_TTU_RawData_Long_AllTimepoints$New_ID<-factor(HRV_TTU_RawData_Long_AllTimepoints$New_ID)
HRV_TTU_RawData_Long_AllTimepoints$Original_ID<-factor(HRV_TTU_RawData_Long_AllTimepoints$Original_ID)
#rename columns to be more intuitive
colnames(HRV_TTU_RawData_Long_AllTimepoints)[3]<-"TaskLoadCondition"
colnames(HRV_TTU_RawData_Long_AllTimepoints)[4]<-"Population"
colnames(HRV_TTU_RawData_Long_AllTimepoints)[5]<-"BreakType"
#add contextual names to timepoints
HRV_TTU_RawData_Long_AllTimepoints["Block"]<-NA
HRV_TTU_RawData_Long_AllTimepoints$Block[which(HRV_TTU_RawData_Long_AllTimepoints$Timepoint=="T1")]="Baseline"
HRV_TTU_RawData_Long_AllTimepoints$Block[which(HRV_TTU_RawData_Long_AllTimepoints$Timepoint=="T2")]="Experimental"
HRV_TTU_RawData_Long_AllTimepoints$Block[which(HRV_TTU_RawData_Long_AllTimepoints$Timepoint=="T3")]="Experimental"
HRV_TTU_RawData_Long_AllTimepoints$Block[which(HRV_TTU_RawData_Long_AllTimepoints$Timepoint=="T4")]="Experimental"
HRV_TTU_RawData_Long_AllTimepoints$Block[which(HRV_TTU_RawData_Long_AllTimepoints$Timepoint=="T5")]="Experimental"
HRV_TTU_RawData_Long_AllTimepoints$Block[which(HRV_TTU_RawData_Long_AllTimepoints$Timepoint=="T6")]="Experimental"
HRV_TTU_RawData_Long_AllTimepoints$Block[which(HRV_TTU_RawData_Long_AllTimepoints$Timepoint=="T7")]="Experimental"
HRV_TTU_RawData_Long_AllTimepoints$Block[which(HRV_TTU_RawData_Long_AllTimepoints$Timepoint=="T8")]="Break"
HRV_TTU_RawData_Long_AllTimepoints$Block[which(HRV_TTU_RawData_Long_AllTimepoints$Timepoint=="T9")]="PostBreak"
HRV_TTU_RawData_Long_AllTimepoints$Block[which(HRV_TTU_RawData_Long_AllTimepoints$Timepoint=="T10")]="PostBreak"

#create a numeric time column that is centered on the baseline for interpretive modeling results
HRV_TTU_RawData_Long_AllTimepoints["Time"]<-as.numeric(HRV_TTU_RawData_Long_AllTimepoints$Timepoint)
HRV_TTU_RawData_Long_AllTimepoints$Time<-HRV_TTU_RawData_Long_AllTimepoints$Time-1
```

Create piecewise, time period predictors for growth curve model.

The predictors will only work for balanced data. For reference,
slope12, slope27, slope78, slope89, and slope 910 lead to intercepts at
T1, T2, T7, T8, and T9, respectively.

```
HRV_TTU_RawData_Long_AllTimepoints$slope12=HRV_TTU_RawData_Long_AllTimepoints$Time
HRV_TTU_RawData_Long_AllTimepoints$slope12[which(HRV_TTU_RawData_Long_AllTimepoints$Timepoint=="T1")]=0
HRV_TTU_RawData_Long_AllTimepoints$slope12[which(HRV_TTU_RawData_Long_AllTimepoints$Timepoint=="T2")]=1
HRV_TTU_RawData_Long_AllTimepoints$slope12[which(HRV_TTU_RawData_Long_AllTimepoints$Timepoint=="T3")]=1
HRV_TTU_RawData_Long_AllTimepoints$slope12[which(HRV_TTU_RawData_Long_AllTimepoints$Timepoint=="T4")]=1
HRV_TTU_RawData_Long_AllTimepoints$slope12[which(HRV_TTU_RawData_Long_AllTimepoints$Timepoint=="T5")]=1
HRV_TTU_RawData_Long_AllTimepoints$slope12[which(HRV_TTU_RawData_Long_AllTimepoints$Timepoint=="T6")]=1
HRV_TTU_RawData_Long_AllTimepoints$slope12[which(HRV_TTU_RawData_Long_AllTimepoints$Timepoint=="T7")]=1
HRV_TTU_RawData_Long_AllTimepoints$slope12[which(HRV_TTU_RawData_Long_AllTimepoints$Timepoint=="T8")]=1
HRV_TTU_RawData_Long_AllTimepoints$slope12[which(HRV_TTU_RawData_Long_AllTimepoints$Timepoint=="T9")]=1
HRV_TTU_RawData_Long_AllTimepoints$slope12[which(HRV_TTU_RawData_Long_AllTimepoints$Timepoint=="T10")]=1
HRV_TTU_RawData_Long_AllTimepoints$slope27=HRV_TTU_RawData_Long_AllTimepoints$Time
HRV_TTU_RawData_Long_AllTimepoints$slope27[which(HRV_TTU_RawData_Long_AllTimepoints$Timepoint=="T1")]=0
HRV_TTU_RawData_Long_AllTimepoints$slope27[which(HRV_TTU_RawData_Long_AllTimepoints$Timepoint=="T2")]=0
HRV_TTU_RawData_Long_AllTimepoints$slope27[which(HRV_TTU_RawData_Long_AllTimepoints$Timepoint=="T3")]=1
HRV_TTU_RawData_Long_AllTimepoints$slope27[which(HRV_TTU_RawData_Long_AllTimepoints$Timepoint=="T4")]=2
HRV_TTU_RawData_Long_AllTimepoints$slope27[which(HRV_TTU_RawData_Long_AllTimepoints$Timepoint=="T5")]=3
HRV_TTU_RawData_Long_AllTimepoints$slope27[which(HRV_TTU_RawData_Long_AllTimepoints$Timepoint=="T6")]=4
HRV_TTU_RawData_Long_AllTimepoints$slope27[which(HRV_TTU_RawData_Long_AllTimepoints$Timepoint=="T7")]=5
HRV_TTU_RawData_Long_AllTimepoints$slope27[which(HRV_TTU_RawData_Long_AllTimepoints$Timepoint=="T8")]=5
HRV_TTU_RawData_Long_AllTimepoints$slope27[which(HRV_TTU_RawData_Long_AllTimepoints$Timepoint=="T9")]=5
HRV_TTU_RawData_Long_AllTimepoints$slope27[which(HRV_TTU_RawData_Long_AllTimepoints$Timepoint=="T10")]=5
HRV_TTU_RawData_Long_AllTimepoints$slope78=HRV_TTU_RawData_Long_AllTimepoints$Time
HRV_TTU_RawData_Long_AllTimepoints$slope78[which(HRV_TTU_RawData_Long_AllTimepoints$Timepoint=="T1")]=0
HRV_TTU_RawData_Long_AllTimepoints$slope78[which(HRV_TTU_RawData_Long_AllTimepoints$Timepoint=="T2")]=0
HRV_TTU_RawData_Long_AllTimepoints$slope78[which(HRV_TTU_RawData_Long_AllTimepoints$Timepoint=="T3")]=0
HRV_TTU_RawData_Long_AllTimepoints$slope78[which(HRV_TTU_RawData_Long_AllTimepoints$Timepoint=="T4")]=0
HRV_TTU_RawData_Long_AllTimepoints$slope78[which(HRV_TTU_RawData_Long_AllTimepoints$Timepoint=="T5")]=0
HRV_TTU_RawData_Long_AllTimepoints$slope78[which(HRV_TTU_RawData_Long_AllTimepoints$Timepoint=="T6")]=0
HRV_TTU_RawData_Long_AllTimepoints$slope78[which(HRV_TTU_RawData_Long_AllTimepoints$Timepoint=="T7")]=0
HRV_TTU_RawData_Long_AllTimepoints$slope78[which(HRV_TTU_RawData_Long_AllTimepoints$Timepoint=="T8")]=1
HRV_TTU_RawData_Long_AllTimepoints$slope78[which(HRV_TTU_RawData_Long_AllTimepoints$Timepoint=="T9")]=1
HRV_TTU_RawData_Long_AllTimepoints$slope78[which(HRV_TTU_RawData_Long_AllTimepoints$Timepoint=="T10")]=1
HRV_TTU_RawData_Long_AllTimepoints$slope89=HRV_TTU_RawData_Long_AllTimepoints$Time
HRV_TTU_RawData_Long_AllTimepoints$slope89[which(HRV_TTU_RawData_Long_AllTimepoints$Timepoint=="T1")]=0
HRV_TTU_RawData_Long_AllTimepoints$slope89[which(HRV_TTU_RawData_Long_AllTimepoints$Timepoint=="T2")]=0
HRV_TTU_RawData_Long_AllTimepoints$slope89[which(HRV_TTU_RawData_Long_AllTimepoints$Timepoint=="T3")]=0
HRV_TTU_RawData_Long_AllTimepoints$slope89[which(HRV_TTU_RawData_Long_AllTimepoints$Timepoint=="T4")]=0
HRV_TTU_RawData_Long_AllTimepoints$slope89[which(HRV_TTU_RawData_Long_AllTimepoints$Timepoint=="T5")]=0
HRV_TTU_RawData_Long_AllTimepoints$slope89[which(HRV_TTU_RawData_Long_AllTimepoints$Timepoint=="T6")]=0
HRV_TTU_RawData_Long_AllTimepoints$slope89[which(HRV_TTU_RawData_Long_AllTimepoints$Timepoint=="T7")]=0
HRV_TTU_RawData_Long_AllTimepoints$slope89[which(HRV_TTU_RawData_Long_AllTimepoints$Timepoint=="T8")]=0
HRV_TTU_RawData_Long_AllTimepoints$slope89[which(HRV_TTU_RawData_Long_AllTimepoints$Timepoint=="T9")]=1
HRV_TTU_RawData_Long_AllTimepoints$slope89[which(HRV_TTU_RawData_Long_AllTimepoints$Timepoint=="T10")]=1
HRV_TTU_RawData_Long_AllTimepoints$slope910=HRV_TTU_RawData_Long_AllTimepoints$Time
HRV_TTU_RawData_Long_AllTimepoints$slope910[which(HRV_TTU_RawData_Long_AllTimepoints$Timepoint=="T1")]=0
HRV_TTU_RawData_Long_AllTimepoints$slope910[which(HRV_TTU_RawData_Long_AllTimepoints$Timepoint=="T2")]=0
HRV_TTU_RawData_Long_AllTimepoints$slope910[which(HRV_TTU_RawData_Long_AllTimepoints$Timepoint=="T3")]=0
HRV_TTU_RawData_Long_AllTimepoints$slope910[which(HRV_TTU_RawData_Long_AllTimepoints$Timepoint=="T4")]=0
HRV_TTU_RawData_Long_AllTimepoints$slope910[which(HRV_TTU_RawData_Long_AllTimepoints$Timepoint=="T5")]=0
HRV_TTU_RawData_Long_AllTimepoints$slope910[which(HRV_TTU_RawData_Long_AllTimepoints$Timepoint=="T6")]=0
HRV_TTU_RawData_Long_AllTimepoints$slope910[which(HRV_TTU_RawData_Long_AllTimepoints$Timepoint=="T7")]=0
HRV_TTU_RawData_Long_AllTimepoints$slope910[which(HRV_TTU_RawData_Long_AllTimepoints$Timepoint=="T8")]=0
HRV_TTU_RawData_Long_AllTimepoints$slope910[which(HRV_TTU_RawData_Long_AllTimepoints$Timepoint=="T9")]=0
HRV_TTU_RawData_Long_AllTimepoints$slope910[which(HRV_TTU_RawData_Long_AllTimepoints$Timepoint=="T10")]=1
```

# Data Exploration

First, we are going to explore what our data looks like by plotting
it over the 10 time points for both low and high task load.

```
HRV_TTU_RawData_Long_AllTimepoints$RMSSD_ms<-as.numeric(HRV_TTU_RawData_Long_AllTimepoints$RMSSD_ms)
color_WL <- c("green","hotpink")
linetype_WL<-c(1,2)
color_ID_32 <- c("red","orange","blue","purple","black","yellow","lavender","lightblue","gray3","aquamarine","darkgreen","pink", "maroon", "darkblue","gold","red","orange","blue","purple","black","yellow","lavender","lightblue","gray3","aquamarine","darkgreen","pink", "maroon", "darkblue","gold","lightgray","darkorange4")

graph_RMSSD<-ggplot(subset.data.frame(HRV_TTU_RawData_Long_AllTimepoints,HRV_TTU_RawData_Long_AllTimepoints$Timeperiod!="last5"), aes(x=Timepoint, y=RMSSD_ms, group=New_ID)) + geom_line(aes(colour=TaskLoadCondition, lty=TaskLoadCondition), linewidth=1) + geom_point(aes(fill=factor(New_ID)),size=3, shape=21, stroke=0) +
  ggtitle("RMSSD across all the time periods (T1-T10) for each participant in both low and high taskload") + labs(y="RMSSD [ms]", x="Time period") +  
  scale_fill_manual(values=color_ID_32) + scale_color_manual(values=color_WL,breaks=c("L","H"),labels = c("Low","High")) +
  scale_linetype_manual(values=linetype_WL,breaks=c("L","H"),labels = c("Low","High")) +
  labs(fill="Participant", colour="Task load", lty="Task load", x="Time period [5 minutes of experimental time]") +
  theme(axis.ticks.x=element_blank(),
        legend.position = "right",
        panel.grid.major = element_blank(),
        panel.grid.minor = element_blank(),
        panel.background = element_blank(),
        axis.line.x = element_line(colour = "black"),
        axis.line.y = element_line(colour = "black"),
        text=element_text(color="black", size=50),
        axis.text=element_text(color="black", size=40),
        strip.background = element_rect(color = "black",fill = "white"))
graph_RMSSD + geom_line(aes(x=Timepoint, y=mean(RMSSD_ms)), color = "black", linewidth=4, linetype=10)
```

Based on this graph, we need to remove the participant with
near-impossible RMSSD (New\_ID=32). Remove this participant and
replot.

```
color_ID_31 <- c("red","orange","blue","purple","black","yellow","lavender","lightblue","gray3","aquamarine","darkgreen","pink", "maroon", "darkblue","gold","red","orange","blue","purple","black","yellow","lavender","lightblue","gray3","aquamarine","darkgreen","pink", "maroon", "darkblue","gold","darkorange4")

graph_RMSSD_NewID32removed<-ggplot(subset.data.frame(HRV_TTU_RawData_Long_AllTimepoints,HRV_TTU_RawData_Long_AllTimepoints$Timeperiod!="last5" & HRV_TTU_RawData_Long_AllTimepoints$New_ID!=32), aes(x=Timepoint, y=RMSSD_ms, group=New_ID)) + geom_line(aes(colour=TaskLoadCondition, lty=TaskLoadCondition), linewidth=1) + geom_point(aes(fill=factor(New_ID)),size=3, shape=21, stroke=0) + 
                                    ggtitle("RMSSD across all the time periods (T1-T10) for each participant (excluding New_ID 32) in both low and high taskload") +
                                    labs(y="RMSSD [ms]", x="Time period") +  scale_fill_manual(values=color_ID_31) + scale_colour_manual(values=color_WL,                                                   breaks=c("L","H"),labels=c("Low","High")) + 
                                    scale_linetype_manual(values=linetype_WL,breaks=c("L","H"),labels=c("Low","High")) +
                                    labs(fill="Participant", colour="Task load", lty="Task load", x="Time period [5 minutes of experimental time]") +
                                    labs(fill="New_ID", colour="Task load") +
                                    theme(axis.ticks.x=element_blank(),
                                          legend.position = "right",
                                          panel.grid.major = element_blank(),
                                          panel.grid.minor = element_blank(),
                                          panel.background = element_blank(),
                                          axis.line.x = element_line(colour = "black"),
                                          axis.line.y = element_line(colour = "black"),
                                          text=element_text(color="black", size=50),
                                          axis.text=element_text(color="black", size=40),
                                          strip.background = element_rect(color = "black",fill = "white"))
graph_RMSSD_NewID32removed + geom_line(aes(x=Timepoint, y=mean(RMSSD_ms)), color = "black", linewidth=4, linetype=10)
```

## Examine the distribution of RMSSD and then identify outliers.

```
hist(subset.data.frame(HRV_TTU_RawData_Long_AllTimepoints, HRV_TTU_RawData_Long_AllTimepoints$New_ID!=32 & HRV_TTU_RawData_Long_AllTimepoints$Timeperiod!="last5")$RMSSD_ms, main="Histogram of all RMSSD values collapsed across all time period [ms]", xlab="RMSSD [ms]")
```

```
boxplot(subset.data.frame(HRV_TTU_RawData_Long_AllTimepoints, HRV_TTU_RawData_Long_AllTimepoints$New_ID!=32 & HRV_TTU_RawData_Long_AllTimepoints$Timeperiod!="last5")$RMSSD_ms, main="Boxplot of all RMSSD values collapsed across all time period [ms]")
```

```
boxplot.stats(subset.data.frame(HRV_TTU_RawData_Long_AllTimepoints, HRV_TTU_RawData_Long_AllTimepoints$New_ID!=32 & HRV_TTU_RawData_Long_AllTimepoints$Timeperiod!="last5")$RMSSD_ms)
```

```
## $stats
## [1]  8.978997 21.957057 31.573310 43.046725 71.168315
## 
## $n
## [1] 310
## 
## $conf
## [1] 29.68077 33.46585
## 
## $out
## numeric(0)
```

```
qqnorm(subset.data.frame(HRV_TTU_RawData_Long_AllTimepoints, HRV_TTU_RawData_Long_AllTimepoints$New_ID!=32 & HRV_TTU_RawData_Long_AllTimepoints$Timeperiod!="last5")$RMSSD_ms, main='QQPlot of RMSSD')
qqline(subset.data.frame(HRV_TTU_RawData_Long_AllTimepoints, HRV_TTU_RawData_Long_AllTimepoints$New_ID!=32 & HRV_TTU_RawData_Long_AllTimepoints$Timeperiod!="last5")$RMSSD_ms)
```

```
boxplot(data=subset.data.frame(HRV_TTU_RawData_Long_AllTimepoints, HRV_TTU_RawData_Long_AllTimepoints$New_ID!=32 & HRV_TTU_RawData_Long_AllTimepoints$Timeperiod!="last5"), RMSSD_ms~Timepoint, main="Boxplot of all RMSSD values for each time period [ms]")
```

## Summary of Data Exploration

There is substantial variation in the RMSSD values across
participants and there are some outliers across the participants. This
may have been why the QQplot did not look promising for normality.
However, when looking at boxplots on an individual and time period
basis, it seems these differences may be informative to the growth curve
models. Time looks similar to an average RMSSD increasing over time.
Also, the researchers and co-authors of the paper have decided RMSSD is
a measure that is comprehensive enough for the paper’s research goals.
In summary, we may proceed with building growth curve models of RMSSD
solely.

Now the plan is to model the data and assess the best fitting model
by testing its adherence to the 3 assumptions of linearity,
homoscedacity, and normality of residuals. We also need to test the
Model for the Means and Model for the Variance is close enough to the
Saturated Means, Unstructured Variance model (i.e., the model known as
the “Total Answer Key”). If so, we will test if modeling RMSSD as a
generalized growth curve model and/or fit and assess alternative
covariance structures as they may meet the 3 necessary assumptions.

#Data Transformation Jumping ahead - 2 of 3 assumptions were violated
for the final model selected in the first iteration of model building
(it was a growth curve model with slope12, 27, 89 as random effects and
slope78, 910 as fixed effects). So we transform the outcome variable
(i.e., RMSSD) via the natural log (ln) to test if this addresses the
failure of those assumptions. This transformation has been done in other
works (see Tung et al., 2021).

Transform and plot said transformed data to see what it looks
like.

```
HRV_TTU_Long_AllNeedTimeSubIDs<-subset.data.frame(HRV_TTU_RawData_Long_AllTimepoints, 
                                                  HRV_TTU_RawData_Long_AllTimepoints$New_ID!=32 &
                                                  HRV_TTU_RawData_Long_AllTimepoints$Timeperiod!="last5",
                                                  select=c("New_ID", "Original_ID", "TaskLoadCondition", "Population",
                                                           "BreakType","FileName", "Timepoint", "Timeperiod", 
                                                           "BeatsCorrected_Percent", "RMSSD_ms", "Block", "Time",               
                                                           "slope12", "slope27","slope78","slope89","slope910"))
HRV_TTU_Long_AllNeedTimeSubIDs["ln_RMSSD_ms"]<-log(HRV_TTU_Long_AllNeedTimeSubIDs$RMSSD_ms)

graph_lnRMSSD_NewID32removed<-ggplot(HRV_TTU_Long_AllNeedTimeSubIDs, aes(x=Timepoint, y=ln_RMSSD_ms, group=New_ID)) +
                                    geom_line(aes(colour=TaskLoadCondition, lty=TaskLoadCondition), linewidth=1) +
                                    geom_point(aes(fill=factor(New_ID)), size=3, shape=21, stroke=0) + 
                                    ggtitle("ln(RMSSD) across all the time periods (T1-T10) for each participant (excluding New_ID 32) in both low and high task
                                            load") + labs(y="ln(RMSSD)", x="Time period") + scale_fill_manual(values=color_ID_31) +
                                    scale_colour_manual(values=color_WL, breaks=c("L","H"),labels = c("Low","High")) +                                                                                  scale_linetype_manual(values=linetype_WL,breaks=c("L","H"),labels=c("Low","High")) +
                                    labs(fill="Participant", colour="Task load", lty="Task load", x="Time period [5 minutes of experimental time]") +
                                        theme(axis.ticks.x=element_blank(),
                                        legend.position = "right",
                                        panel.grid.major = element_blank(),
                                        panel.grid.minor = element_blank(),
                                        panel.background = element_blank(),
                                        axis.line.x = element_line(colour = "black"),
                                        axis.line.y = element_line(colour = "black"),
                                        text=element_text(color="black", size=50),
                                        axis.text=element_text(color="black", size=40),
                                        strip.background = element_rect(color = "black",fill = "white"))
graph_lnRMSSD_NewID32removed + geom_line(aes(x=Timepoint, y=mean(ln_RMSSD_ms)), color = "darkblue", linewidth=4, linetype=10)
```

There is no obvious issue with how the transformed data looks -
variance looks the same across all time points and it is hard to say
what the trend is as this plot does not demarcate it by New\_ID. To do
this, need to look at the next plot.

# Model Building - Unconditional Growth Curve Models

## Unconditional Growth Curve Model - First iteration

### Empty Means, Random Intercept Model

The empty means, random intercept model basically assess if we have
longitudinal data. So build the model and show results using
Satterthwaite DDF including -2LL as deviance.

```
lnEmptyRI=lmer(data=HRV_TTU_Long_AllNeedTimeSubIDs, REML=TRUE, formula=ln_RMSSD_ms~1+(1|New_ID))
summary(lnEmptyRI, ddf="Satterthwaite"); llikAIC(lnEmptyRI, chkREML=FALSE)
```

```
## Linear mixed model fit by REML. t-tests use Satterthwaite's method [
## lmerModLmerTest]
## Formula: ln_RMSSD_ms ~ 1 + (1 | New_ID)
##    Data: HRV_TTU_Long_AllNeedTimeSubIDs
## 
## REML criterion at convergence: -70.6
## 
## Scaled residuals: 
##     Min      1Q  Median      3Q     Max 
## -3.9223 -0.4869 -0.0175  0.6332  2.6421 
## 
## Random effects:
##  Groups   Name        Variance Std.Dev.
##  New_ID   (Intercept) 0.15320  0.3914  
##  Residual             0.03129  0.1769  
## Number of obs: 310, groups:  New_ID, 31
## 
## Fixed effects:
##             Estimate Std. Error       df t value Pr(>|t|)    
## (Intercept)  3.42800    0.07101 30.00000   48.27   <2e-16 ***
## ---
## Signif. codes:  0 '***' 0.001 '**' 0.01 '*' 0.05 '.' 0.1 ' ' 1
```

```
## $logLik
## 'log Lik.' 35.28179 (df=3)
## 
## $AICtab
##       AIC       BIC    logLik  deviance  df.resid 
## -64.56359 -53.35387  35.28179 -70.56359 307.00000
```

Get Intraclass correlation (ICC) to assure we have longitudinal
data.

```
performance::icc(lnEmptyRI) #had to add performance:: to get this line of code to work because icc was masked by another package
```

```
## # Intraclass Correlation Coefficient
## 
##     Adjusted ICC: 0.830
##   Unadjusted ICC: 0.830
```

ICC=0.830, meaning 83.0% of the variance is BETWEEN participants!

Does the random intercept improve model fit? Compare a model with and
without the random intercept to test if ICC is significant>0. Note:
the 2nd row is comparing a model without the parameter listed in the 1st
column.

```
ranova(lnEmptyRI, reduce.term=TRUE)
```

```
## ANOVA-like table for random-effects: Single term deletions
## 
## Model:
## ln_RMSSD_ms ~ (1 | New_ID)
##              npar   logLik    AIC    LRT Df Pr(>Chisq)    
## <none>          3   35.282 -64.56                         
## (1 | New_ID)    2 -176.405 356.81 423.37  1  < 2.2e-16 ***
## ---
## Signif. codes:  0 '***' 0.001 '**' 0.01 '*' 0.05 '.' 0.1 ' ' 1
```

ICC is sig, (LRT(1)=423.37, p<.001) suggesting we have
longitudinal data that needs to be modeled accordingly, which we can do
with growth curve models.

### ‘Total Answer Key’

Saturated Means, Unstructured Variance Model in GLS (AKA the TOTAL
ANSWER KEY): We want our final unconditional growth curve model to match
this model as closely as possible (explanation as to why is on p. 220 of
Hoffman (2015)).

Note: Any line of code commented out is because it takes a very long
time to run and sometimes does not execute. It is already loaded in the
provided R object.

```
lnSatUN = gls(data=HRV_TTU_Long_AllNeedTimeSubIDs, method="REML", model=ln_RMSSD_ms~1+factor(Timepoint), 
            correlation=corSymm(form=~as.numeric(Timepoint)|New_ID), # Unstructured correlations
            weights=varIdent(form=~1|as.numeric(Timepoint)))         # Heterogeneous variances
```

Show results with -2LL, incorrect DDF, and total leftover
variance.

```
summary(lnSatUN); -2*logLik(lnSatUN)
```

```
## Generalized least squares fit by REML
##   Model: ln_RMSSD_ms ~ 1 + factor(Timepoint) 
##   Data: HRV_TTU_Long_AllNeedTimeSubIDs 
##         AIC      BIC   logLik
##   -118.3773 122.3686 124.1887
## 
## Correlation Structure: General
##  Formula: ~as.numeric(Timepoint) | New_ID 
##  Parameter estimate(s):
##  Correlation: 
##    1     2     3     4     5     6     7     8     9    
## 2  0.782                                                
## 3  0.771 0.969                                          
## 4  0.704 0.953 0.950                                    
## 5  0.747 0.957 0.947 0.967                              
## 6  0.832 0.852 0.837 0.818 0.846                        
## 7  0.787 0.857 0.857 0.855 0.889 0.923                  
## 8  0.760 0.853 0.855 0.838 0.856 0.902 0.901            
## 9  0.818 0.891 0.892 0.886 0.900 0.921 0.892 0.865      
## 10 0.820 0.896 0.876 0.854 0.866 0.904 0.888 0.859 0.952
## Variance function:
##  Structure: Different standard deviations per stratum
##  Formula: ~1 | as.numeric(Timepoint) 
##  Parameter estimates:
##         1         2         3         4         5         6         7         8 
## 1.0000000 1.0159783 0.9913690 0.8643629 0.9206099 0.9032788 0.8399524 0.8884006 
##         9        10 
## 0.9235264 0.9161944 
## 
## Coefficients:
##                         Value  Std.Error  t-value p-value
## (Intercept)          3.300604 0.08170494 40.39663  0.0000
## factor(Timepoint)T2  0.062632 0.05434718  1.15245  0.2501
## factor(Timepoint)T3  0.066454 0.05501093  1.20802  0.2280
## factor(Timepoint)T4  0.063875 0.05952964  1.07300  0.2841
## factor(Timepoint)T5  0.085940 0.05609144  1.53214  0.1265
## factor(Timepoint)T6  0.112830 0.04572951  2.46732  0.0142
## factor(Timepoint)T7  0.184802 0.05057995  3.65367  0.0003
## factor(Timepoint)T8  0.191143 0.05417679  3.52813  0.0005
## factor(Timepoint)T9  0.237872 0.04779494  4.97693  0.0000
## factor(Timepoint)T10 0.268425 0.04737403  5.66609  0.0000
## 
##  Correlation: 
##                      (Intr) f(T)T2 f(T)T3 f(T)T4 f(T)T5 f(T)T6 f(T)T7 f(T)T8
## factor(Timepoint)T2  -0.308                                                 
## factor(Timepoint)T3  -0.349  0.930                                          
## factor(Timepoint)T4  -0.538  0.895  0.899                                   
## factor(Timepoint)T5  -0.454  0.903  0.891  0.946                            
## factor(Timepoint)T6  -0.444  0.633  0.620  0.685  0.688                     
## factor(Timepoint)T7  -0.547  0.668  0.691  0.780  0.795  0.830              
## factor(Timepoint)T8  -0.490  0.680  0.702  0.746  0.741  0.802  0.819       
## factor(Timepoint)T9  -0.418  0.735  0.753  0.806  0.801  0.798  0.761  0.721
## factor(Timepoint)T10 -0.428  0.745  0.716  0.749  0.730  0.755  0.753  0.710
##                      f(T)T9
## factor(Timepoint)T2        
## factor(Timepoint)T3        
## factor(Timepoint)T4        
## factor(Timepoint)T5        
## factor(Timepoint)T6        
## factor(Timepoint)T7        
## factor(Timepoint)T8        
## factor(Timepoint)T9        
## factor(Timepoint)T10  0.881
## 
## Standardized residuals:
##         Min          Q1         Med          Q3         Max 
## -2.59911940 -0.82762801  0.05354963  0.78227929  2.09767653 
## 
## Residual standard error: 0.4549139 
## Degrees of freedom: 310 total; 300 residual
```

```
## 'log Lik.' -248.3773 (df=65)
```

Total variance per occasion is created using SD multiplier.

```
summary(lnSatUN)$sigma^2
```

```
## [1] 0.2069466
```

Show R and RCORR matrices for first person (R is slightly off).

```
lnR=getVarCov(lnSatUN, New_ID="6", type="marginal"); lnR
```

```
## Marginal variance covariance matrix
##          [,1]    [,2]    [,3]    [,4]    [,5]    [,6]    [,7]    [,8]    [,9]
##  [1,] 0.20695 0.16450 0.15826 0.12585 0.14240 0.15549 0.13682 0.13965 0.15632
##  [2,] 0.16450 0.21361 0.20199 0.17319 0.18529 0.16178 0.15129 0.15925 0.17305
##  [3,] 0.15826 0.20199 0.20339 0.16841 0.17892 0.15515 0.14770 0.15586 0.16897
##  [4,] 0.12585 0.17319 0.16841 0.15461 0.15927 0.13222 0.12853 0.13317 0.14634
##  [5,] 0.14240 0.18529 0.17892 0.15927 0.17539 0.14567 0.14219 0.14495 0.15838
##  [6,] 0.15549 0.16178 0.15515 0.13222 0.14567 0.16885 0.14491 0.14978 0.15894
##  [7,] 0.13682 0.15129 0.14770 0.12853 0.14219 0.14491 0.14600 0.13909 0.14322
##  [8,] 0.13965 0.15925 0.15586 0.13317 0.14495 0.14978 0.13909 0.16333 0.14692
##  [9,] 0.15632 0.17305 0.16897 0.14634 0.15838 0.15894 0.14322 0.14692 0.17650
## [10,] 0.15554 0.17252 0.16471 0.13992 0.15113 0.15480 0.14136 0.14473 0.16674
##         [,10]
##  [1,] 0.15554
##  [2,] 0.17252
##  [3,] 0.16471
##  [4,] 0.13992
##  [5,] 0.15113
##  [6,] 0.15480
##  [7,] 0.14136
##  [8,] 0.14473
##  [9,] 0.16674
## [10,] 0.17371
##   Standard Deviations: 0.45491 0.46218 0.45099 0.39321 0.4188 0.41091 0.38211 0.40415 0.42012 0.41679
```

```
lnRCORR=round(corMatrix(lnSatUN$modelStruct$corStruct)[[4]],3); lnRCORR
```

```
##        [,1]  [,2]  [,3]  [,4]  [,5]  [,6]  [,7]  [,8]  [,9] [,10]
##  [1,] 1.000 0.782 0.771 0.704 0.747 0.832 0.787 0.760 0.818 0.820
##  [2,] 0.782 1.000 0.969 0.953 0.957 0.852 0.857 0.853 0.891 0.896
##  [3,] 0.771 0.969 1.000 0.950 0.947 0.837 0.857 0.855 0.892 0.876
##  [4,] 0.704 0.953 0.950 1.000 0.967 0.818 0.855 0.838 0.886 0.854
##  [5,] 0.747 0.957 0.947 0.967 1.000 0.846 0.889 0.856 0.900 0.866
##  [6,] 0.832 0.852 0.837 0.818 0.846 1.000 0.923 0.902 0.921 0.904
##  [7,] 0.787 0.857 0.857 0.855 0.889 0.923 1.000 0.901 0.892 0.888
##  [8,] 0.760 0.853 0.855 0.838 0.856 0.902 0.901 1.000 0.865 0.859
##  [9,] 0.818 0.891 0.892 0.886 0.900 0.921 0.892 0.865 1.000 0.952
## [10,] 0.820 0.896 0.876 0.854 0.866 0.904 0.888 0.859 0.952 1.000
```

p-value of the F-test is based on Satterthwaite DDF. This F-test is
telling us if the HRV metric significantly differs between the 10 time
points or not (does not specify which time points it differs on
specifically as it is an omnibus test).

```
anova(lnSatUN)
```

```
## Denom. DF: 300 
##                   numDF  F-value p-value
## (Intercept)           1 3516.122  <.0001
## factor(Timepoint)     9    9.609  <.0001
```

###COLLAPSE PREDICTED VALUES OF LN PLOT ###COLLAPSE PREDICTED VALUES
OF LN PLOT ###COLLAPSE PREDICTED VALUES OF LN PLOT

Any line of code commented out below is because it takes a very long
time to run and sometimes does not execute: We already have the results
stored from a previous run of the data, so make sure those are loaded in
to the global environment (i.e., the RData object that is in the load()
line of code has been run) before running this block of code.

```
#emmeans(ref_grid(lnSatUN), pairwise~Timepoint, adjust="none") # tried mode="df.error" #takes ~10-15 mins to run...beware!
#emmeans_SatUN_table<-as.data.frame(emmeans(ref_grid(lnSatUN), pairwise~Timepoint, adjust="none")$emmeans)
#emmeans_SatUN_table<-as.data.frame(emmeans_SatUN_table)
graph_emmeansRMSSD<-ggplot(emmeans_SatUN_table, aes(x=Timepoint, y=emmean, group=1)) + 
                  geom_line(colour="black", linewidth=5, linetype="dashed") + geom_point(colour="black", shape=19, size=10) +
                  ggtitle("Estimated marginal means of ln(RMSSD) across all time periods (T1-T10) in both low and high taskload") +
                  labs(y="Estimated marginal mean of ln(RMSSD)", x="Time period") + 
                  theme(axis.ticks.x=element_blank(),
                      legend.position = "none", 
                      panel.grid.major = element_blank(),
                      panel.grid.minor = element_blank(),
                      panel.background = element_blank(),
                      axis.line.x = element_line(colour = "black"),
                      axis.line.y = element_line(colour = "black"),
                      text=element_text(color="black", size=50),
                      axis.text=element_text(color="black", size=40),
                      strip.background = element_rect(color = "black",fill = "white"))
graph_emmeansRMSSD
```

Summary: The estimated marginal means for each time period shows, on
average, cardiac vagal tone increases immediately upon starting the
vigilance task, stabilizes for Block 1, increases slightly during Block
2, and then increases more dramatically in Block 3. Then, it is
estimated to plateau during the break, and then continually increases
during the post-break vigilance task (Block 4).

### Trial-and-Error of Maximal Models

Build the maximal model and then run ranova to see what random effect
can be dropped in order to assure parsimony. Once this process cycles
through, check to see that this model’s Model for the Means and Model
for the Variance does not significantly differ from the Saturated Means,
Unstructured Variance Model.

Maximal log-normal generalized multilevel model aka one with all the
piecewise slopes and a quadratic time slope for slope27 per the results
of the Saturated Means, Unstructured Variance model.

*Note*: This is a generalized model even though we are using
lmer because in this case, transforming the outcome variable with the
natural log and modeling with general linear modeling methods is the
same as generalized multilevel model with a log-normal link
function.

The below model has convergence issues and/or were singular:
Therefore we do not use them in our model building process.

```
ln.MaxModel.AllRand<-lmer(data=HRV_TTU_Long_AllNeedTimeSubIDs, REML=TRUE,
                          formula=ln_RMSSD_ms~(1+slope12+slope27+I(slope27^2)+slope78+slope89+slope910)+
                                  (1+slope12+slope27+I(slope27^2)+slope89+slope910|New_ID),
                                    control=lmerControl(optimizer = "nlminbwrap", optCtrl=list(maxfun=4e5)))
```

```
## Warning in optwrap(optimizer, devfun, getStart(start, rho$pp), lower =
## rho$lower, : convergence code 1 from nlminbwrap: iteration limit reached without
## convergence (10)
```

```
## Warning in checkConv(attr(opt, "derivs"), opt$par, ctrl = control$checkConv, :
## unable to evaluate scaled gradient
```

```
## Warning in checkConv(attr(opt, "derivs"), opt$par, ctrl = control$checkConv, :
## Model failed to converge: degenerate Hessian with 1 negative eigenvalues
```

```
## Warning: Model failed to converge with 1 negative eigenvalue: -2.3e+01
```

```
allFit(ln.MaxModel.AllRand)
```

```
## bobyqa :
```

```
## Warning: Model failed to converge with 2 negative eigenvalues: -1.7e-04 -1.1e-01
```

```
## [OK]
## Nelder_Mead :
```

```
## Warning in checkConv(attr(opt, "derivs"), opt$par, ctrl = control$checkConv, :
## Model failed to converge with max|grad| = 3.28932 (tol = 0.002, component 1)
```

```
## [OK]
## nlminbwrap :
```

```
## Warning in optwrap(optimizer, devfun, getStart(start, rho$pp), lower =
## rho$lower, : convergence code 1 from nlminbwrap: iteration limit reached without
## convergence (10)
```

```
## Warning in checkConv(attr(opt, "derivs"), opt$par, ctrl = control$checkConv, :
## unable to evaluate scaled gradient
```

```
## Warning in checkConv(attr(opt, "derivs"), opt$par, ctrl = control$checkConv, :
## Model failed to converge: degenerate Hessian with 1 negative eigenvalues
```

```
## Warning: Model failed to converge with 1 negative eigenvalue: -2.3e+01
```

```
## [OK]
## nmkbw :
```

```
## Warning in ctrl[namc] <- control: number of items to replace is not a multiple
## of replacement length
```

```
## Warning in checkConv(attr(opt, "derivs"), opt$par, ctrl = control$checkConv, :
## unable to evaluate scaled gradient
```

```
## Warning in checkConv(attr(opt, "derivs"), opt$par, ctrl = control$checkConv, :
## Model failed to converge: degenerate Hessian with 1 negative eigenvalues
```

```
## Warning: Model failed to converge with 1 negative eigenvalue: -9.9e-02
```

```
## [OK]
## optimx.L-BFGS-B : [OK]
## nloptwrap.NLOPT_LN_NELDERMEAD :
```

```
## Warning in checkConv(attr(opt, "derivs"), opt$par, ctrl = control$checkConv, :
## Model failed to converge with max|grad| = 1.86009 (tol = 0.002, component 1)
```

```
## [OK]
## nloptwrap.NLOPT_LN_BOBYQA :
```

```
## Warning in checkConv(attr(opt, "derivs"), opt$par, ctrl = control$checkConv, :
## unable to evaluate scaled gradient
```

```
## Warning in checkConv(attr(opt, "derivs"), opt$par, ctrl = control$checkConv, :
## Model failed to converge: degenerate Hessian with 2 negative eigenvalues
```

```
## Warning: Model failed to converge with 2 negative eigenvalues: -9.2e-03 -3.0e-01
```

```
## [OK]
```

```
## original model:
## ln_RMSSD_ms ~ (1 + slope12 + slope27 + I(slope27^2) + slope78 + slope89 + slo... 
## data:  HRV_TTU_Long_AllNeedTimeSubIDs 
## optimizers (7): bobyqa, Nelder_Mead, nlminbwrap, nmkbw, optimx.L-BFGS-B,nloptwrap.NLOPT_LN_N...
## differences in negative log-likelihoods:
## max= 7.64 ; std dev= 3.19
```

Maximal model of the ln(RMSSD) will need to be reduced because it was
singular. In order to know what to reduce it to, reduce random effects 1
by 1 - starting with slope12, all else the same.

```
#Removing slope12 as a random effect to see if we can get a non-singular model, all else random effects
ln.Fix12.AllElseRand<-lmer(data=HRV_TTU_Long_AllNeedTimeSubIDs, REML=TRUE, 
                           formula=ln_RMSSD_ms~(1+slope12+slope27+I(slope27^2)+slope78+slope89+slope910)+
                           (1+slope27+I(slope27^2)+slope78+slope89+slope910|New_ID), 
                           control=lmerControl(optimizer="bobyqa", optCtrl=list(maxfun=4e5))) #singular, DO NOT USE!
  
#Removing slope27 random effect to see if we can get a non-singular model, all else random effects *Cannot remove linear slope27 without removing quad slope27, Hoffman (2015)*
ln.Fix27.AllElseRand<-lmer(data=HRV_TTU_Long_AllNeedTimeSubIDs, REML=TRUE, formula=ln_RMSSD_ms~(1+slope12+slope27+I(slope27^2)+slope78+slope89+slope910)+(1+slope12+slope78+slope89+slope910|New_ID), control = lmerControl(optimizer = "bobyqa")) #singular, DO NOT USE!
  
#Removing quadratic slope27 random effect to see if we can get a non-singular model, all else random effects 
ln.Fix27.AllElseRand<-lmer(data=HRV_TTU_Long_AllNeedTimeSubIDs, REML=TRUE, formula=ln_RMSSD_ms~(1+slope12+slope27+I(slope27^2)+slope78+slope89+slope910)+
                           (1+slope12+slope27+slope78+slope89+slope910|New_ID), control = lmerControl(optimizer = "bobyqa")) #singular, DO NOT USE!
  
#Removing slope78 random effect to see if we can get a non-singular model, all else random effects
ln.Fix78.AllElseRand<-lmer(data=HRV_TTU_Long_AllNeedTimeSubIDs, REML=TRUE, formula=ln_RMSSD_ms~(1+slope12+slope27+I(slope27^2)+slope78+slope89+slope910)+
                               (1+slope12+slope27+I(slope27^2)+slope89+slope910|New_ID), control = lmerControl(optimizer = "optimx", optCtrl=list(method='L-BFGS-B'))) #singular, convergence issues, DO NOT USE!
```

```
## Warning in optwrap(optimizer, devfun, getStart(start, rho$pp), lower =
## rho$lower, : convergence code 1 from optimx: none
```

```
## Warning: Model failed to converge with 1 negative eigenvalue: -1.9e+00
```

```
#Removing slope89 random effect to see if we can get a non-singular model, all else random effects
ln.Fix89.AllElseRand<-lmer(data=HRV_TTU_Long_AllNeedTimeSubIDs, REML=TRUE, formula=ln_RMSSD_ms~(1+slope12+slope27+I(slope27^2)+slope78+slope89+slope910)+                       (1+slope12+slope27+I(slope27^2)+slope78+slope910|New_ID), control = lmerControl(optimizer = "Nelder_Mead", optCtrl=list(maxfun=4e5))) #singular, convergence issues, DO NOT USE!
```

```
## Warning in checkConv(attr(opt, "derivs"), opt$par, ctrl = control$checkConv, :
## unable to evaluate scaled gradient
```

```
## Warning in checkConv(attr(opt, "derivs"), opt$par, ctrl = control$checkConv, :
## Model failed to converge: degenerate Hessian with 3 negative eigenvalues
```

```
## Warning: Model failed to converge with 3 negative eigenvalues: -3.2e-01 -2.5e+00
## -3.5e+00
```

```
#Removing slope910 random effect to see if we can get a non-singular model, all else random effects
ln.Fix910ElseRand<-lmer(data=HRV_TTU_Long_AllNeedTimeSubIDs, REML=TRUE, formula=ln_RMSSD_ms~(1+slope12+slope27+I(slope27^2)+slope78+slope89+slope910)+
                            (1+slope12+slope27+I(slope27^2)+slope78+slope89|New_ID), control = lmerControl(optimizer = "bobyqa", optCtrl=list(maxfun=4e5)))#singular, convergence issues, DO NOT USE!
```

```
## Warning: Model failed to converge with 2 negative eigenvalues: -5.5e-03 -2.3e-02
```

Removing slopes 1 by 1 from the random effect structure did not give
us a maximal model that was also non-singular, which we need to find a
suitable unconditional growth curve model. Now need to remove two slopes
at a time from the random effects structure to see if we can get a
maximal, non-singular model.

```
ln.Fix910.27quad.AllElseRand<-lmer(data=HRV_TTU_Long_AllNeedTimeSubIDs, REML=TRUE, formula=ln_RMSSD_ms~(1+slope12+slope27+I(slope27^2)+slope78+slope89+slope910)+
                                      (1+slope12+slope27+slope78+slope89|New_ID), control = lmerControl(optimizer = "bobyqa"))
```

ln.Fix910.27quad.AllElseRand is non-singular. Now check to see if
other combos of this nature are not singular (1 by 1) and compare.

```
ln.Fix78.27quad.AllElseRand<-lmer(data=HRV_TTU_Long_AllNeedTimeSubIDs, REML=TRUE, formula=ln_RMSSD_ms~(1+slope12+slope27+I(slope27^2)+slope78+slope89+slope910)+
                          (1+slope12+slope27+slope89+slope910|New_ID), control = lmerControl(optimizer = "bobyqa")) #singular, DO NOT USE!
ln.Fix27.27quad.AllElseRand<-lmer(data=HRV_TTU_Long_AllNeedTimeSubIDs, REML=TRUE, formula=ln_RMSSD_ms~(1+slope12+slope27+I(slope27^2)+slope78+slope89+slope910)+
                          (1+slope12+slope78+slope89+slope910|New_ID), control = lmerControl(optimizer = "bobyqa")) #singular, DO NOT USE!
ln.Fix12.27quad.AllElseRand<-lmer(data=HRV_TTU_Long_AllNeedTimeSubIDs, REML=TRUE, formula=ln_RMSSD_ms~(1+slope12+slope27+I(slope27^2)+slope78+slope89+slope910)+
                          (1+slope27+slope78+slope89+slope910|New_ID), control = lmerControl(optimizer = "bobyqa")) #singular, DO NOT USE!
```

No other combination leads to a non-singular result, meaning
ln.Fix910.27quad.AllElseRand is the maximal, non-singular model. Review
summary stats and ranova.

```
summary(ln.Fix910.27quad.AllElseRand); llikAIC(ln.Fix910.27quad.AllElseRand, chkREML=FALSE)
```

```
## Linear mixed model fit by REML. t-tests use Satterthwaite's method [
## lmerModLmerTest]
## Formula: ln_RMSSD_ms ~ (1 + slope12 + slope27 + I(slope27^2) + slope78 +  
##     slope89 + slope910) + (1 + slope12 + slope27 + slope78 +  
##     slope89 | New_ID)
##    Data: HRV_TTU_Long_AllNeedTimeSubIDs
## Control: lmerControl(optimizer = "bobyqa")
## 
## REML criterion at convergence: -189.3
## 
## Scaled residuals: 
##     Min      1Q  Median      3Q     Max 
## -3.3860 -0.5054  0.0559  0.4727  2.5919 
## 
## Random effects:
##  Groups   Name        Variance Std.Dev. Corr                   
##  New_ID   (Intercept) 0.195899 0.44260                         
##           slope12     0.090463 0.30077  -0.30                  
##           slope27     0.001941 0.04405  -0.19 -0.63            
##           slope78     0.009593 0.09794   0.04  0.18 -0.18      
##           slope89     0.026038 0.16136   0.23 -0.05 -0.26 -0.70
##  Residual             0.011049 0.10512                         
## Number of obs: 310, groups:  New_ID, 31
## 
## Fixed effects:
##               Estimate Std. Error        df t value Pr(>|t|)    
## (Intercept)    3.30060    0.08170  29.99997  40.396  < 2e-16 ***
## slope12        0.06824    0.05973  31.86120   1.143  0.26173    
## slope27       -0.01889    0.01793 171.91146  -1.053  0.29368    
## I(slope27^2)   0.00819    0.00309 153.00021   2.651  0.00888 ** 
## slope78        0.01261    0.03096  37.81504   0.407  0.68614    
## slope89        0.04673    0.03941  38.15747   1.186  0.24301    
## slope910       0.03055    0.02670 153.00009   1.144  0.25427    
## ---
## Signif. codes:  0 '***' 0.001 '**' 0.01 '*' 0.05 '.' 0.1 ' ' 1
## 
## Correlation of Fixed Effects:
##             (Intr) slop12 slop27 I(27^2 slop78 slop89
## slope12     -0.333                                   
## slope27     -0.082 -0.446                            
## I(slop27^2)  0.000  0.172 -0.861                     
## slope78      0.022  0.074  0.151 -0.333              
## slope89      0.163 -0.036 -0.083  0.000 -0.583       
## slope910     0.000  0.000  0.000  0.000  0.000 -0.339
```

```
## $logLik
## 'log Lik.' 94.62867 (df=23)
## 
## $AICtab
##        AIC        BIC     logLik   deviance   df.resid 
## -143.25734  -57.31618   94.62867 -189.25734  287.00000
```

Use ranova to test if a reduced random effects structure does not
lead to compromising model fit.

```
ranova(ln.Fix910.27quad.AllElseRand, reduce.terms = TRUE)
```

```
## ANOVA-like table for random-effects: Single term deletions
## 
## Model:
## ln_RMSSD_ms ~ slope12 + slope27 + I(slope27^2) + slope78 + slope89 + slope910 + (1 + slope12 + slope27 + slope78 + slope89 | New_ID)
##                                                                 npar logLik
## <none>                                                            23 94.629
## slope12 in (1 + slope12 + slope27 + slope78 + slope89 | New_ID)   18 63.093
## slope27 in (1 + slope12 + slope27 + slope78 + slope89 | New_ID)   18 72.161
## slope78 in (1 + slope12 + slope27 + slope78 + slope89 | New_ID)   18 92.388
## slope89 in (1 + slope12 + slope27 + slope78 + slope89 | New_ID)   18 86.536
##                                                                      AIC    LRT
## <none>                                                          -143.257       
## slope12 in (1 + slope12 + slope27 + slope78 + slope89 | New_ID)  -90.186 63.071
## slope27 in (1 + slope12 + slope27 + slope78 + slope89 | New_ID) -108.322 44.935
## slope78 in (1 + slope12 + slope27 + slope78 + slope89 | New_ID) -148.775  4.482
## slope89 in (1 + slope12 + slope27 + slope78 + slope89 | New_ID) -137.071 16.186
##                                                                 Df Pr(>Chisq)
## <none>                                                                       
## slope12 in (1 + slope12 + slope27 + slope78 + slope89 | New_ID)  5  2.814e-12
## slope27 in (1 + slope12 + slope27 + slope78 + slope89 | New_ID)  5  1.495e-08
## slope78 in (1 + slope12 + slope27 + slope78 + slope89 | New_ID)  5   0.482313
## slope89 in (1 + slope12 + slope27 + slope78 + slope89 | New_ID)  5   0.006333
##                                                                    
## <none>                                                             
## slope12 in (1 + slope12 + slope27 + slope78 + slope89 | New_ID) ***
## slope27 in (1 + slope12 + slope27 + slope78 + slope89 | New_ID) ***
## slope78 in (1 + slope12 + slope27 + slope78 + slope89 | New_ID)    
## slope89 in (1 + slope12 + slope27 + slope78 + slope89 | New_ID) ** 
## ---
## Signif. codes:  0 '***' 0.001 '**' 0.01 '*' 0.05 '.' 0.1 ' ' 1
```

The ranova results suggest slope78 can be reduced to a fixed effect
only. Complete thorough backwards selection process to verify.

### Trial-and-Error of Parsimonious Models

With ln.Fix910.27quad.AllElseRand, drop each other random slope 1 by
1 to see if it leads to a significantly worse fit.

```
ln.Fix910.27quad.12.AllElseRand<-lmer(data=HRV_TTU_Long_AllNeedTimeSubIDs, REML=TRUE,
                                      formula=ln_RMSSD_ms~(1+slope12+slope27+I(slope27^2)+slope78+slope89+slope910)+(1+slope27+slope78+slope89|New_ID), control = lmerControl(optimizer = "bobyqa")) #singular, DO NOT USE!
```

Dropping random slope12 lead to a singular fit; drop random linear
slope27 and test for a significantly worse model fit.

```
ln.Fix910.27quad.27.AllElseRand<-lmer(data=HRV_TTU_Long_AllNeedTimeSubIDs, REML=TRUE, formula=ln_RMSSD_ms~(1+slope12+slope27+I(slope27^2)+slope78+slope89+slope910)+
                                      (1+slope12+slope78+slope89|New_ID), control=lmerControl(optimizer="bobyqa"))
anova(ln.Fix910.27quad.27.AllElseRand,ln.Fix910.27quad.AllElseRand, refit=FALSE)
```

```
## Data: HRV_TTU_Long_AllNeedTimeSubIDs
## Models:
## ln.Fix910.27quad.27.AllElseRand: ln_RMSSD_ms ~ (1 + slope12 + slope27 + I(slope27^2) + slope78 + slope89 + slope910) + (1 + slope12 + slope78 + slope89 | New_ID)
## ln.Fix910.27quad.AllElseRand: ln_RMSSD_ms ~ (1 + slope12 + slope27 + I(slope27^2) + slope78 + slope89 + slope910) + (1 + slope12 + slope27 + slope78 + slope89 | New_ID)
##                                 npar     AIC     BIC logLik deviance  Chisq Df
## ln.Fix910.27quad.27.AllElseRand   18 -108.32 -41.064 72.161  -144.32          
## ln.Fix910.27quad.AllElseRand      23 -143.26 -57.316 94.629  -189.26 44.935  5
##                                 Pr(>Chisq)    
## ln.Fix910.27quad.27.AllElseRand               
## ln.Fix910.27quad.AllElseRand     1.495e-08 ***
## ---
## Signif. codes:  0 '***' 0.001 '**' 0.01 '*' 0.05 '.' 0.1 ' ' 1
```

Dropping random linear slope27 lead to a significantly worse fit;
drop random slope78 and test for a significantly worse fit.

```
ln.Fix910.27quad.78.AllElseRand<-lmer(data=HRV_TTU_Long_AllNeedTimeSubIDs, REML=TRUE, formula=ln_RMSSD_ms~(1+slope12+slope27+I(slope27^2)+slope78+slope89+slope910)+
                                      (1+slope12+slope27+slope89|New_ID), control=lmerControl(optimizer = "bobyqa"))
anova(ln.Fix910.27quad.78.AllElseRand,ln.Fix910.27quad.AllElseRand, refit=FALSE)
```

```
## Data: HRV_TTU_Long_AllNeedTimeSubIDs
## Models:
## ln.Fix910.27quad.78.AllElseRand: ln_RMSSD_ms ~ (1 + slope12 + slope27 + I(slope27^2) + slope78 + slope89 + slope910) + (1 + slope12 + slope27 + slope89 | New_ID)
## ln.Fix910.27quad.AllElseRand: ln_RMSSD_ms ~ (1 + slope12 + slope27 + I(slope27^2) + slope78 + slope89 + slope910) + (1 + slope12 + slope27 + slope78 + slope89 | New_ID)
##                                 npar     AIC     BIC logLik deviance  Chisq Df
## ln.Fix910.27quad.78.AllElseRand   18 -148.78 -81.517 92.388  -184.78          
## ln.Fix910.27quad.AllElseRand      23 -143.26 -57.316 94.629  -189.26 4.4819  5
##                                 Pr(>Chisq)
## ln.Fix910.27quad.78.AllElseRand           
## ln.Fix910.27quad.AllElseRand        0.4823
```

Dropping random linear slope78 did *not* lead to a
significantly worse fit; drop random slope89 and test for a
significantly worse fit.

```
ln.Fix910.27quad.89.AllElseRand<-lmer(data=HRV_TTU_Long_AllNeedTimeSubIDs, REML=TRUE, formula=ln_RMSSD_ms~(1+slope12+slope27+I(slope27^2)+slope78+slope89+slope910)+
                                      (1+slope12+slope27+slope78|New_ID), control = lmerControl(optimizer = "bobyqa"))
anova(ln.Fix910.27quad.89.AllElseRand,ln.Fix910.27quad.AllElseRand, refit=FALSE)
```

```
## Data: HRV_TTU_Long_AllNeedTimeSubIDs
## Models:
## ln.Fix910.27quad.89.AllElseRand: ln_RMSSD_ms ~ (1 + slope12 + slope27 + I(slope27^2) + slope78 + slope89 + slope910) + (1 + slope12 + slope27 + slope78 | New_ID)
## ln.Fix910.27quad.AllElseRand: ln_RMSSD_ms ~ (1 + slope12 + slope27 + I(slope27^2) + slope78 + slope89 + slope910) + (1 + slope12 + slope27 + slope78 + slope89 | New_ID)
##                                 npar     AIC     BIC logLik deviance  Chisq Df
## ln.Fix910.27quad.89.AllElseRand   18 -137.07 -69.813 86.536  -173.07          
## ln.Fix910.27quad.AllElseRand      23 -143.26 -57.316 94.629  -189.26 16.186  5
##                                 Pr(>Chisq)   
## ln.Fix910.27quad.89.AllElseRand              
## ln.Fix910.27quad.AllElseRand      0.006333 **
## ---
## Signif. codes:  0 '***' 0.001 '**' 0.01 '*' 0.05 '.' 0.1 ' ' 1
```

Dropping random linear slope89 lead to a significantly worse fit; new
maximal model is ln.Fix910.27quad.78.AllElseRand. Review summary stats
and ranova.

```
summary(ln.Fix910.27quad.78.AllElseRand); llikAIC(ln.Fix910.27quad.78.AllElseRand, chkREML=FALSE)
```

```
## Linear mixed model fit by REML. t-tests use Satterthwaite's method [
## lmerModLmerTest]
## Formula: ln_RMSSD_ms ~ (1 + slope12 + slope27 + I(slope27^2) + slope78 +  
##     slope89 + slope910) + (1 + slope12 + slope27 + slope89 |      New_ID)
##    Data: HRV_TTU_Long_AllNeedTimeSubIDs
## Control: lmerControl(optimizer = "bobyqa")
## 
## REML criterion at convergence: -184.8
## 
## Scaled residuals: 
##     Min      1Q  Median      3Q     Max 
## -3.1254 -0.5207  0.0452  0.4749  2.5513 
## 
## Random effects:
##  Groups   Name        Variance Std.Dev. Corr             
##  New_ID   (Intercept) 0.194867 0.44144                   
##           slope12     0.087715 0.29617  -0.29            
##           slope27     0.001838 0.04287  -0.19 -0.60      
##           slope89     0.014837 0.12181   0.32  0.04 -0.51
##  Residual             0.012081 0.10992                   
## Number of obs: 310, groups:  New_ID, 31
## 
## Fixed effects:
##                Estimate Std. Error         df t value Pr(>|t|)    
## (Intercept)    3.300604   0.081705  29.999897  40.396   <2e-16 ***
## slope12        0.068242   0.059493  32.234760   1.147   0.2598    
## slope27       -0.018891   0.018508 201.783693  -1.021   0.3086    
## I(slope27^2)   0.008190   0.003231 183.000001   2.535   0.0121 *  
## slope78        0.012609   0.026643 182.999993   0.473   0.6366    
## slope89        0.046729   0.035469  69.291011   1.317   0.1920    
## slope910       0.030553   0.027919 182.999994   1.094   0.2752    
## ---
## Signif. codes:  0 '***' 0.001 '**' 0.01 '*' 0.05 '.' 0.1 ' ' 1
## 
## Correlation of Fixed Effects:
##             (Intr) slop12 slop27 I(27^2 slop78 slop89
## slope12     -0.336                                   
## slope27     -0.076 -0.433                            
## I(slop27^2)  0.000  0.181 -0.873                     
## slope78      0.000 -0.026  0.240 -0.404              
## slope89      0.194  0.021 -0.131  0.000 -0.412       
## slope910     0.000  0.000  0.000  0.000  0.000 -0.394
```

```
## $logLik
## 'log Lik.' 92.38774 (df=18)
## 
## $AICtab
##        AIC        BIC     logLik   deviance   df.resid 
## -148.77547  -81.51717   92.38774 -184.77547  292.00000
```

```
ranova(ln.Fix910.27quad.78.AllElseRand, reduce.terms = TRUE)
```

```
## ANOVA-like table for random-effects: Single term deletions
## 
## Model:
## ln_RMSSD_ms ~ slope12 + slope27 + I(slope27^2) + slope78 + slope89 + slope910 + (1 + slope12 + slope27 + slope89 | New_ID)
##                                                       npar logLik      AIC
## <none>                                                  18 92.388 -148.775
## slope12 in (1 + slope12 + slope27 + slope89 | New_ID)   14 62.783  -97.566
## slope27 in (1 + slope12 + slope27 + slope89 | New_ID)   14 70.261 -112.523
## slope89 in (1 + slope12 + slope27 + slope89 | New_ID)   14 84.821 -141.642
##                                                          LRT Df Pr(>Chisq)    
## <none>                                                                        
## slope12 in (1 + slope12 + slope27 + slope89 | New_ID) 59.210  4  4.252e-12 ***
## slope27 in (1 + slope12 + slope27 + slope89 | New_ID) 44.253  4  5.685e-09 ***
## slope89 in (1 + slope12 + slope27 + slope89 | New_ID) 15.134  4   0.004432 ** 
## ---
## Signif. codes:  0 '***' 0.001 '**' 0.01 '*' 0.05 '.' 0.1 ' ' 1
```

Reducing random effects structure led to significantly worse model
fit. Therefore, retain random effects model structure and compare with
the saturated means and unstructured variance model.

##

## The selected unconditional growth curve model - First Iter

### Model Code

```
ln.Fix910.78.AllElseRand<-lmer(data=HRV_TTU_Long_AllNeedTimeSubIDs, REML=TRUE, formula=ln_RMSSD_ms~(1+slope12+slope27+I(slope27^2)+slope78+slope89+slope910)+
                                      (1+slope12+slope27+I(slope27^2)+slope89|New_ID), control=lmerControl(optimizer="bobyqa", optCtrl=list(maxfun=5e5)))
ranova(ln.Fix910.78.AllElseRand, reduce.terms = TRUE)
```

```
## ANOVA-like table for random-effects: Single term deletions
## 
## Model:
## ln_RMSSD_ms ~ slope12 + slope27 + I(slope27^2) + slope78 + slope89 + slope910 + (1 + slope12 + slope27 + I(slope27^2) + slope89 | New_ID)
##                                                                           npar
## <none>                                                                      23
## slope12 in (1 + slope12 + slope27 + I(slope27^2) + slope89 | New_ID)        18
## slope27 in (1 + slope12 + slope27 + I(slope27^2) + slope89 | New_ID)        18
## I(slope27^2) in (1 + slope12 + slope27 + I(slope27^2) + slope89 | New_ID)   18
## slope89 in (1 + slope12 + slope27 + I(slope27^2) + slope89 | New_ID)        18
##                                                                           logLik
## <none>                                                                    96.011
## slope12 in (1 + slope12 + slope27 + I(slope27^2) + slope89 | New_ID)      64.788
## slope27 in (1 + slope12 + slope27 + I(slope27^2) + slope89 | New_ID)      94.278
## I(slope27^2) in (1 + slope12 + slope27 + I(slope27^2) + slope89 | New_ID) 92.388
## slope89 in (1 + slope12 + slope27 + I(slope27^2) + slope89 | New_ID)      87.400
##                                                                                AIC
## <none>                                                                    -146.023
## slope12 in (1 + slope12 + slope27 + I(slope27^2) + slope89 | New_ID)       -93.576
## slope27 in (1 + slope12 + slope27 + I(slope27^2) + slope89 | New_ID)      -152.556
## I(slope27^2) in (1 + slope12 + slope27 + I(slope27^2) + slope89 | New_ID) -148.775
## slope89 in (1 + slope12 + slope27 + I(slope27^2) + slope89 | New_ID)      -138.800
##                                                                              LRT
## <none>                                                                          
## slope12 in (1 + slope12 + slope27 + I(slope27^2) + slope89 | New_ID)      62.447
## slope27 in (1 + slope12 + slope27 + I(slope27^2) + slope89 | New_ID)       3.466
## I(slope27^2) in (1 + slope12 + slope27 + I(slope27^2) + slope89 | New_ID)  7.247
## slope89 in (1 + slope12 + slope27 + I(slope27^2) + slope89 | New_ID)      17.223
##                                                                           Df
## <none>                                                                      
## slope12 in (1 + slope12 + slope27 + I(slope27^2) + slope89 | New_ID)       5
## slope27 in (1 + slope12 + slope27 + I(slope27^2) + slope89 | New_ID)       5
## I(slope27^2) in (1 + slope12 + slope27 + I(slope27^2) + slope89 | New_ID)  5
## slope89 in (1 + slope12 + slope27 + I(slope27^2) + slope89 | New_ID)       5
##                                                                           Pr(>Chisq)
## <none>                                                                              
## slope12 in (1 + slope12 + slope27 + I(slope27^2) + slope89 | New_ID)        3.79e-12
## slope27 in (1 + slope12 + slope27 + I(slope27^2) + slope89 | New_ID)        0.628476
## I(slope27^2) in (1 + slope12 + slope27 + I(slope27^2) + slope89 | New_ID)   0.202898
## slope89 in (1 + slope12 + slope27 + I(slope27^2) + slope89 | New_ID)        0.004096
##                                                                              
## <none>                                                                       
## slope12 in (1 + slope12 + slope27 + I(slope27^2) + slope89 | New_ID)      ***
## slope27 in (1 + slope12 + slope27 + I(slope27^2) + slope89 | New_ID)         
## I(slope27^2) in (1 + slope12 + slope27 + I(slope27^2) + slope89 | New_ID)    
## slope89 in (1 + slope12 + slope27 + I(slope27^2) + slope89 | New_ID)      ** 
## ---
## Signif. codes:  0 '***' 0.001 '**' 0.01 '*' 0.05 '.' 0.1 ' ' 1
```

Per the summary and ranova (i.e., significant fixed effect of
quadratic slope27 and all models being a significant worse fit when each
of the remaining random effects is removed), the final unconditional
growth curve model is ln.Fix910.27quad.78.AllElseRand. Now test model
fixed effects assumptions (see code comments to understand why the
following plots and tests were run). But first, store this unconditional
growth curve model in a more informative name.

```
ln.Fix910.27quad.78.Rand.12.27lin.89<-lmer(data=HRV_TTU_Long_AllNeedTimeSubIDs, REML=TRUE,
                                           formula=ln_RMSSD_ms~(1+slope12+slope27+I(slope27^2)+slope78+slope89+slope910)+(1+slope12+slope27+slope89|New_ID),
                                           control=lmerControl(optimizer="bobyqa"))
```

## 

### Summary of the selected unconditional growth curve model - First Iteration

Summary of the unconditional growth curve model.

```
summary(ln.Fix910.27quad.78.Rand.12.27lin.89, ddf="Satterthwaite")
```

```
## Linear mixed model fit by REML. t-tests use Satterthwaite's method [
## lmerModLmerTest]
## Formula: ln_RMSSD_ms ~ (1 + slope12 + slope27 + I(slope27^2) + slope78 +  
##     slope89 + slope910) + (1 + slope12 + slope27 + slope89 |      New_ID)
##    Data: HRV_TTU_Long_AllNeedTimeSubIDs
## Control: lmerControl(optimizer = "bobyqa")
## 
## REML criterion at convergence: -184.8
## 
## Scaled residuals: 
##     Min      1Q  Median      3Q     Max 
## -3.1254 -0.5207  0.0452  0.4749  2.5513 
## 
## Random effects:
##  Groups   Name        Variance Std.Dev. Corr             
##  New_ID   (Intercept) 0.194867 0.44144                   
##           slope12     0.087715 0.29617  -0.29            
##           slope27     0.001838 0.04287  -0.19 -0.60      
##           slope89     0.014837 0.12181   0.32  0.04 -0.51
##  Residual             0.012081 0.10992                   
## Number of obs: 310, groups:  New_ID, 31
## 
## Fixed effects:
##                Estimate Std. Error         df t value Pr(>|t|)    
## (Intercept)    3.300604   0.081705  29.999897  40.396   <2e-16 ***
## slope12        0.068242   0.059493  32.234760   1.147   0.2598    
## slope27       -0.018891   0.018508 201.783693  -1.021   0.3086    
## I(slope27^2)   0.008190   0.003231 183.000001   2.535   0.0121 *  
## slope78        0.012609   0.026643 182.999993   0.473   0.6366    
## slope89        0.046729   0.035469  69.291011   1.317   0.1920    
## slope910       0.030553   0.027919 182.999994   1.094   0.2752    
## ---
## Signif. codes:  0 '***' 0.001 '**' 0.01 '*' 0.05 '.' 0.1 ' ' 1
## 
## Correlation of Fixed Effects:
##             (Intr) slop12 slop27 I(27^2 slop78 slop89
## slope12     -0.336                                   
## slope27     -0.076 -0.433                            
## I(slop27^2)  0.000  0.181 -0.873                     
## slope78      0.000 -0.026  0.240 -0.404              
## slope89      0.194  0.021 -0.131  0.000 -0.412       
## slope910     0.000  0.000  0.000  0.000  0.000 -0.394
```

Fixed effects of the unconditional growth curve model

```
fixef(ln.Fix910.27quad.78.Rand.12.27lin.89);length(fixef(ln.Fix910.27quad.78.Rand.12.27lin.89))
```

```
##  (Intercept)      slope12      slope27 I(slope27^2)      slope78      slope89 
##  3.300604483  0.068242432 -0.018890592  0.008189781  0.012608799  0.046729394 
##     slope910 
##  0.030553171
```

```
## [1] 7
```

Compute and store conditional means for each time period from values
of time predictors.

```
#CondMeans_OrigMod<-data.frame(Estimate=as.numeric(), Std.Error=as.numeric(), df=as.numeric(), t value=as.numeric(), Pr(>|t|)=as.numeric())
CondMeans_OrigMod<-contest1D(ln.Fix910.27quad.78.Rand.12.27lin.89, ddf="Satterthwaite", L=c(1,0,0,0,0,0,0))#;CondMeans_OrigMod
CondMeans_OrigMod<-rbind(CondMeans_OrigMod,contest1D(ln.Fix910.27quad.78.Rand.12.27lin.89, ddf="Satterthwaite", L=c(1,1,1,1,0,0,0)))
CondMeans_OrigMod<-rbind(CondMeans_OrigMod,contest1D(ln.Fix910.27quad.78.Rand.12.27lin.89, ddf="Satterthwaite", L=c(1,1,2,4,0,0,0)))
CondMeans_OrigMod<-rbind(CondMeans_OrigMod,contest1D(ln.Fix910.27quad.78.Rand.12.27lin.89, ddf="Satterthwaite", L=c(1,1,3,9,0,0,0)))
CondMeans_OrigMod<-rbind(CondMeans_OrigMod,contest1D(ln.Fix910.27quad.78.Rand.12.27lin.89, ddf="Satterthwaite", L=c(1,1,4,16,0,0,0)))
CondMeans_OrigMod<-rbind(CondMeans_OrigMod,contest1D(ln.Fix910.27quad.78.Rand.12.27lin.89, ddf="Satterthwaite", L=c(1,1,5,25,0,0,0)))
CondMeans_OrigMod<-rbind(CondMeans_OrigMod,contest1D(ln.Fix910.27quad.78.Rand.12.27lin.89, ddf="Satterthwaite", L=c(1,1,6,36,0,0,0)))
CondMeans_OrigMod<-rbind(CondMeans_OrigMod,contest1D(ln.Fix910.27quad.78.Rand.12.27lin.89, ddf="Satterthwaite", L=c(1,1,6,36,1,0,0)))
CondMeans_OrigMod<-rbind(CondMeans_OrigMod,contest1D(ln.Fix910.27quad.78.Rand.12.27lin.89, ddf="Satterthwaite", L=c(1,1,6,36,1,1,0)))
CondMeans_OrigMod<-rbind(CondMeans_OrigMod,contest1D(ln.Fix910.27quad.78.Rand.12.27lin.89, ddf="Satterthwaite", L=c(1,1,6,36,1,1,1)))
CondMeans_OrigMod["Timepoint"]<-c("T1","T2","T3","T4","T5","T6","T7","T8","T9","T10")
CondMeans_OrigMod["Model"]<-c("Random slope12, slope27, slope89; Fixed slope27^2, slope78, slope910 Unconditional Growth Curve Model (Original model based on experimental setup/theoretical rationale)")

emmeans_SatUN_table["Model"]<-c("Saturated Means and Unstructured Variance Model")
emmeans_SatUN_df<-subset.data.frame(emmeans_SatUN_table, select=c(Model,Timepoint,emmean,SE,df))
colnames(emmeans_SatUN_df)<-c("Model","Timepoint","Estimate","SE","df")
CondMeans<-subset.data.frame(CondMeans_OrigMod, select=c("Model","Timepoint","Estimate","Std. Error","df"))
colnames(CondMeans)<-c("Model", "Timepoint","Estimate","SE","df")
CondMeans<-rbind(emmeans_SatUN_df,CondMeans)
CondMeans$Model<-factor(CondMeans$Model, levels = c("Saturated Means and Unstructured Variance Model","Random slope12, slope27, slope89; Fixed slope27^2, slope78, slope910 Unconditional Growth Curve Model (Original model based on experimental setup/theoretical rationale)"))
```

##

## Model Comparison and Assessment - First Iteration

### Need to compare the first iteration unconditional growth curve model against Saturated Means, Unstructured Variance Model aka the “Total Answer Key”

First, visually compare the estimated marginal means of each
model.

```
graph_CompAnsKeyOrigMod_lnRMSSD<-ggplot(CondMeans, aes(x=Timepoint, y=Estimate, group=Model)) + 
                                    geom_line(aes(colour=Model, linetype=Model, linewidth=Model)) + 
                                      scale_colour_manual(labels = ~ stringr::str_wrap(.x, width = 35),values=c("black","darkgray")) + 
                                      scale_linetype_manual(labels = ~ stringr::str_wrap(.x, width = 35),values=c("longdash","dotdash")) + 
                                      scale_discrete_manual("linewidth",labels = ~ stringr::str_wrap(.x, width = 35),values=c(5,3)) + 
                                    geom_point(aes(colour=Model,shape=Model,size=Model)) + 
                                      scale_colour_manual(labels = ~ stringr::str_wrap(.x, width = 35),values=c("black","darkgray")) + 
                                      scale_shape_manual(labels = ~ stringr::str_wrap(.x, width = 35),values=c(19,15)) + 
                                      scale_size_manual(labels = ~ stringr::str_wrap(.x, width = 35),values=c(10,6)) + 
                                    ggtitle("Comparing Models of Means") +
                                    labs(y="Estimated ln(RMSSD)", x="Time period") + 
                                    theme(axis.ticks.x=element_blank(),
                                      legend.position = "right", 
                                      legend.key.width = unit(2, 'cm'), 
                                      legend.key = element_blank(),
                                      legend.text=element_text(margin=margin(t=20),size=30),
                                      #plot.title = element_text(hjust=0.05),
                                      panel.grid.major = element_blank(),
                                      panel.grid.minor = element_blank(),
                                      panel.background = element_blank(),
                                      axis.line.x = element_line(colour = "black"),
                                      axis.line.y = element_line(colour = "black"),
                                      text=element_text(color="black", size=50),
                                      axis.text=element_text(color="black", size=40),
                                      strip.background = element_rect(color = "black",fill = "white"))
graph_CompAnsKeyOrigMod_lnRMSSD
```

This plot is showing that the Model for the Means for the unconditional
growth curve model of interest starts to deviate considerably from the
Model of the Means starting at T3. Of interest is to test if that
deviation is significant, which can be done with statistical tests.

Now, we will asses the suitability this model as our unconditional
growth curve by comparing its Model for the Means and Model for the
Variance with statistical comparison methods.

To do this, we need to first saturate the proposed unconditional
growth curve model with dummy time period predictors. To do this, we
will add the dummy predictors that are not explicitly enumerated in any
of the slopes in order to get an estimated mean for each time point
(i.e., the model is saturated, there is no other time slope predictors
to add). The ultimate goal is to see if adding them leads to a
significantly better model fit. If it does, then we need to rethink the
piecewise time slopes that are currently in our growth curve model and
if it doesn’t then that suggests our piecewise time slope predictors are
sufficient in capturing the way cardiac vagal tone trends over the
course of the vigilance task, break, and post-break vigilance task.

Create dummy time period predictors in order to properly saturate the
model.

```
HRV_TTU_Long_AllNeedTimeSubIDs["T1"]<-0
HRV_TTU_Long_AllNeedTimeSubIDs["T2"]<-0
HRV_TTU_Long_AllNeedTimeSubIDs["T3"]<-0
HRV_TTU_Long_AllNeedTimeSubIDs["T4"]<-0
HRV_TTU_Long_AllNeedTimeSubIDs["T5"]<-0
HRV_TTU_Long_AllNeedTimeSubIDs["T6"]<-0
HRV_TTU_Long_AllNeedTimeSubIDs["T7"]<-0
HRV_TTU_Long_AllNeedTimeSubIDs["T8"]<-0
HRV_TTU_Long_AllNeedTimeSubIDs["T9"]<-0
HRV_TTU_Long_AllNeedTimeSubIDs["T10"]<-0
HRV_TTU_Long_AllNeedTimeSubIDs$T1[which(HRV_TTU_Long_AllNeedTimeSubIDs$Timepoint=="T1")]=1
HRV_TTU_Long_AllNeedTimeSubIDs$T2[which(HRV_TTU_Long_AllNeedTimeSubIDs$Timepoint=="T2")]=1
HRV_TTU_Long_AllNeedTimeSubIDs$T3[which(HRV_TTU_Long_AllNeedTimeSubIDs$Timepoint=="T3")]=1
HRV_TTU_Long_AllNeedTimeSubIDs$T4[which(HRV_TTU_Long_AllNeedTimeSubIDs$Timepoint=="T4")]=1
HRV_TTU_Long_AllNeedTimeSubIDs$T5[which(HRV_TTU_Long_AllNeedTimeSubIDs$Timepoint=="T5")]=1
HRV_TTU_Long_AllNeedTimeSubIDs$T6[which(HRV_TTU_Long_AllNeedTimeSubIDs$Timepoint=="T6")]=1
HRV_TTU_Long_AllNeedTimeSubIDs$T7[which(HRV_TTU_Long_AllNeedTimeSubIDs$Timepoint=="T7")]=1
HRV_TTU_Long_AllNeedTimeSubIDs$T8[which(HRV_TTU_Long_AllNeedTimeSubIDs$Timepoint=="T8")]=1
HRV_TTU_Long_AllNeedTimeSubIDs$T9[which(HRV_TTU_Long_AllNeedTimeSubIDs$Timepoint=="T9")]=1
HRV_TTU_Long_AllNeedTimeSubIDs$T10[which(HRV_TTU_Long_AllNeedTimeSubIDs$Timepoint=="T10")]=1
```

Saturate the Model for the Means of this Unconditional Growth Curve
model in order to test if it is a significantly better growth curve
model than the one we found in our first iteration of model building…if
so, then that would suggests its Model for the Means is not comparable
to that of the Saturated Means, Unstructured Variance model.

Results are based on Satterthwaite DDF including -2LL as
deviance.

```
Saturated.ln.Fix910.27quad.78.Rand.12.27lin.89<-lmer(data=HRV_TTU_Long_AllNeedTimeSubIDs, REML=TRUE,
                                            formula=ln_RMSSD_ms~(1+slope12+slope27+I(slope27^2)+T4+T5+T6+slope78+slope89+slope910)+
                                                               (1+slope12+slope27+slope89|New_ID), control=lmerControl(optimizer="bobyqa"))
summary(Saturated.ln.Fix910.27quad.78.Rand.12.27lin.89, ddf="Satterthwaite")
```

```
## Linear mixed model fit by REML. t-tests use Satterthwaite's method [
## lmerModLmerTest]
## Formula: ln_RMSSD_ms ~ (1 + slope12 + slope27 + I(slope27^2) + T4 + T5 +  
##     T6 + slope78 + slope89 + slope910) + (1 + slope12 + slope27 +  
##     slope89 | New_ID)
##    Data: HRV_TTU_Long_AllNeedTimeSubIDs
## Control: lmerControl(optimizer = "bobyqa")
## 
## REML criterion at convergence: -169.7
## 
## Scaled residuals: 
##      Min       1Q   Median       3Q      Max 
## -3.01065 -0.52827  0.05179  0.47239  2.53079 
## 
## Random effects:
##  Groups   Name        Variance Std.Dev. Corr             
##  New_ID   (Intercept) 0.194712 0.44126                   
##           slope12     0.087483 0.29577  -0.29            
##           slope27     0.001831 0.04279  -0.19 -0.60      
##           slope89     0.014706 0.12127   0.33  0.04 -0.51
##  Residual             0.012236 0.11062                   
## Number of obs: 310, groups:  New_ID, 31
## 
## Fixed effects:
##                Estimate Std. Error         df t value Pr(>|t|)    
## (Intercept)    3.300604   0.081705  29.999949  40.396   <2e-16 ***
## slope12        0.062632   0.060095  33.549903   1.042    0.305    
## slope27       -0.001331   0.035287 199.181430  -0.038    0.970    
## I(slope27^2)   0.005153   0.006438 180.000056   0.800    0.425    
## T4            -0.016706   0.037801 180.000066  -0.442    0.659    
## T5            -0.019076   0.039636 180.000065  -0.481    0.631    
## T6            -0.026927   0.032766 180.000072  -0.822    0.412    
## slope78        0.006340   0.028097 180.000083   0.226    0.822    
## slope89        0.046729   0.035550  69.783645   1.314    0.193    
## slope910       0.030553   0.028097 180.000083   1.087    0.278    
## ---
## Signif. codes:  0 '***' 0.001 '**' 0.01 '*' 0.05 '.' 0.1 ' ' 1
## 
## Correlation of Fixed Effects:
##             (Intr) slop12 slop27 I(27^2 T4     T5     T6     slop78 slop89
## slope12     -0.333                                                        
## slope27     -0.040 -0.340                                                 
## I(slop27^2)  0.000  0.204 -0.964                                          
## T4           0.000  0.104 -0.766  0.795                                   
## T5           0.000  0.133 -0.796  0.804  0.727                            
## T6           0.000  0.120 -0.662  0.636  0.612  0.656                     
## slope78      0.000  0.000  0.020 -0.109  0.037  0.106  0.257              
## slope89      0.193  0.020 -0.068  0.000  0.000  0.000  0.000 -0.395       
## slope910     0.000  0.000  0.000  0.000  0.000  0.000  0.000  0.000 -0.395
```

```
llikAIC(Saturated.ln.Fix910.27quad.78.Rand.12.27lin.89, chkREML=FALSE)
```

```
## $logLik
## 'log Lik.' 84.86384 (df=21)
## 
## $AICtab
##        AIC        BIC     logLik   deviance   df.resid 
## -127.72769  -49.25967   84.86384 -169.72769  289.00000
```

```
fixef(Saturated.ln.Fix910.27quad.78.Rand.12.27lin.89);length(fixef(Saturated.ln.Fix910.27quad.78.Rand.12.27lin.89))
```

```
##  (Intercept)      slope12      slope27 I(slope27^2)           T4           T5 
##  3.300604483  0.062632323 -0.001331342  0.005153068 -0.016706470 -0.019075890 
##           T6      slope78      slope89     slope910 
## -0.026926502  0.006340490  0.046729394  0.030553171
```

```
## [1] 10
```

Does Saturated.ln.Fix910.27quad.78.Rand.12.27lin.89 reproduce
saturated means?

```
contestMD(Saturated.ln.Fix910.27quad.78.Rand.12.27lin.89, ddf="Satterthwaite",
                                                      L=rbind(c(0,0,0,0,1,0,0,0,0,0),
                                                              c(0,0,0,0,0,1,0,0,0,0),
                                                              c(0,0,0,0,0,0,1,0,0,0)))
```

```
##        Sum Sq     Mean Sq NumDF    DenDF  F value    Pr(>F)
## 1 0.008357294 0.002785765     3 180.0001 0.227663 0.8770579
```

F(3,180)=0.227663, p=0.8770579, suggesting that saturating the
unconditional growth curve model did not lead to a significantly better
model fit, suggesting its Model for the Means is not significantly
different than the Model for the Means of the Saturated Means,
Unstructured Variance model. Therefore, this result would suggest to
leave the unconditional growth curve model as is. However, we need to
check the other side of the model, i.e., the Model for the Variance.

Now we will assess if the Model for the Variance of this
unconditional growth curve model is significantly different than the
Model for the Variance of the Saturated Means, Unstructured Variance
Model. This means we keep this growth curve model’s fixed effects
constant, but change the random effects structure to be one with
Unstructured Variance as this is what the Model for the Variance is in
the Saturated Means, Unstructured Variance model.

Logistical detail: Need to use lme instead of lmer as the former
allows the variance structure to be more specifically specified (note:
using REML in GLS for accurate model fitting).

```
lnSameFixUnVarREML = gls(data=HRV_TTU_Long_AllNeedTimeSubIDs, method="REML", 
                         model=ln_RMSSD_ms~1+slope12+slope27+I(slope27^2)+slope78+slope89+slope910, 
                         correlation=corSymm(form=~as.numeric(Timepoint)|New_ID), # Unstructured correlations
                         weights=varIdent(form=~1|(Timepoint)))                   # Heterogeneous variances

#The below is the same model as ln.Fix910.27quad.78.Rand.12.27lin.89 (aka the unconditional model of interest) 
  #but now just fit in LME in order to model compare
lmeREML.ln.Fix910.27quad.78.Rand.12.27lin.89<-lme(data=HRV_TTU_Long_AllNeedTimeSubIDs, method="REML", 
                                            ln_RMSSD_ms~1+slope12+slope27+I(slope27^2)+slope78+slope89+slope910,
                                            random=~1+slope12+slope27+slope89|New_ID)

#Use a LRT to assess if the Model for the Variance of the two models significantly differs
anova(lnSameFixUnVarREML, lmeREML.ln.Fix910.27quad.78.Rand.12.27lin.89)
```

```
##                                              Model df       AIC       BIC
## lnSameFixUnVarREML                               1 62 -133.8787  96.37269
## lmeREML.ln.Fix910.27quad.78.Rand.12.27lin.89     2 18 -148.7755 -81.92828
##                                                 logLik   Test  L.Ratio p-value
## lnSameFixUnVarREML                           128.93937                        
## lmeREML.ln.Fix910.27quad.78.Rand.12.27lin.89  92.38774 1 vs 2 73.10328  0.0038
```

The likelihood ratio test (LRT) result was -2LL=73.10328, p=0.00358,
suggesting the Model for the Variance of the Unconditional Growth Curve
Model is significantly different than the Model for the Variance of the
Saturated Means, Unstructured Variance Model. Therefore, we need to
adjust the random effects structure of the Unconditional Growth Curve
Model as that informs the Model for the Variance. To give us a clue of
how to adjust it specifically, visually compare the Model for the
Variance of this Unconditional Growth Curve Model with the Model for the
Variance of the Saturated Means, Unstructured Variance Model.

In order to visually compare the two Models for the Variance, need to
extract their total variance per parameter.

```
TotalVarAnsKeyOrigMods<-data.frame(Model=character(), Timepoint=character(), TotalVar=numeric())
TotalVarAnsKeyOrigMods[1:10,"Model"]<-"Saturated Means and Unstructured Variance Model"
TotalVarAnsKeyOrigMods[11:20,"Model"]<-"Random slope12, slope27, slope89; Fixed slope27^2, slope78, slope910 Unconditional Growth Curve Model (Original model based on experimental setup/theoretical rationale)"
TotalVarAnsKeyOrigMods$Model<-factor(TotalVarAnsKeyOrigMods$Model, levels=c("Saturated Means and Unstructured Variance Model","Random slope12, slope27, slope89; Fixed slope27^2, slope78, slope910 Unconditional Growth Curve Model (Original model based on experimental setup/theoretical rationale)"))
  
TotalVarAnsKeyOrigMods[1:10,"Timepoint"]<-c("T1","T2","T3","T4","T5","T6","T7","T8","T9","T10")
TotalVarAnsKeyOrigMods[11:20,"Timepoint"]<-c("T1","T2","T3","T4","T5","T6","T7","T8","T9","T10")
TotalVarAnsKeyOrigMods$Timepoint<-factor(TotalVarAnsKeyOrigMods$Timepoint,
                                         levels=c("T1","T2","T3","T4","T5","T6","T7","T8","T9","T10"))  
 
TotalVarAnsKeyOrigMods[1:10,"TotalVar"]<-(diag(getVarCov(lnSatUN, New_ID="6", type="marginal")))
TotalVarAnsKeyOrigMods[11:20,"TotalVar"]<-(diag(getVarCov(lmeREML.ln.Fix910.27quad.78.Rand.12.27lin.89, New_ID="6",
                                                          type="marginal")[[1]]))
row.names(TotalVarAnsKeyOrigMods)<-1:nrow(TotalVarAnsKeyOrigMods)
```

```
graph_CompAnsKeyOrigModTotVar_lnRMSSD<-ggplot(TotalVarAnsKeyOrigMods, aes(x=Timepoint, y=TotalVar, group=Model)) + 
                                      geom_line(aes(colour=Model, linetype=Model, linewidth=Model)) + 
                                        scale_colour_manual(labels = ~stringr::str_wrap(.x, width=                       
                                                                                           35),values=c("black","darkgray")) + 
                                        scale_linetype_manual(labels = ~stringr::str_wrap(.x, width=                             
                                                                                             35),values=c("longdash","dotdash")) + 
                                        scale_discrete_manual("linewidth",labels = ~ stringr::str_wrap(.x, width=35),values=c(5,3))+
                                      geom_point(aes(colour=Model,shape=Model,size=Model)) + 
                                        scale_colour_manual(labels = ~stringr::str_wrap(.x, width=35),values=c("black","darkgray")) + scale_shape_manual(labels =                                                ~stringr::str_wrap(.x, width = 35),values=c(19,15)) + 
                                        scale_size_manual(labels = ~stringr::str_wrap(.x, width = 35),values=c(10,6)) +
                                      ggtitle("Comparing Models of the Variance") +
                                      labs(y="Estimated variance [ln(RMSSD)]", x="Time period") + 
                                      theme(axis.ticks.x=element_blank(),
                                        legend.position = "right", 
                                        legend.key.width = unit(2, 'cm'), 
                                        legend.key = element_blank(),
                                        legend.text=element_text(margin = margin(t = 20),size=30),
                                       #plot.title = element_text(hjust = 0.5),
                                        panel.grid.major = element_blank(),
                                        panel.grid.minor = element_blank(),
                                        panel.background = element_blank(),
                                        axis.line.x = element_line(colour = "black"),
                                        axis.line.y = element_line(colour = "black"),
                                        text=element_text(color="black", size=50),
                                        axis.text=element_text(color="black", size=40),
                                        strip.background = element_rect(color = "black",fill = "white"))
graph_CompAnsKeyOrigModTotVar_lnRMSSD
```

Summary of plot: Just like the Model for the Means comparison, it looks
like the Model for the Variance of the unconditional growth curve model
really starts to deviate at time period 3 and does not seem to be
matched again until time period 9. Given it looks like there is more
variability in the time periods later on in the 30-minute vigilance
task, we should see if there are markedly different trends (and
associated varability) for different phases of the 30-minute vigilance
task. To do this, break up slope27 in to multiple piecewise slopes and
complete the unconditional growth curve modeling analysis again.

## Modeling the Unconditional Growth Curve - Second Iteration

Given the Model for the Variance of
ln.Fix910.27.quad.78.Rand.12.27lin.89 deviates from the Saturated Means,
Unstructured Variance model produced, we need to adjust the model’s
random effects structure and we can do this by adding more random
effects. In order to add more random effects in a unconditional growth
curve model, we need to have more time slopes, which in this case means
breaking up slope27 as it is the only time slope that has more than 2
time periods within it. In order to decide where to break up slope27, we
look at all our plots/analyses thus far. Specifically, when looking at
the estimated marginal means of the Saturated Means, Unstructured
Variance Model, it seems plausible that the slope from T6 to T7 is not
the same slope/cannot be captured within the same single linear or
quadratic slope as that of T2 to T5. Therefore, the model will not
assess model fit when there are two slopes over the 30-min vigilance
task: slope26 and slope67.

Note: The full iteration of this phase of model building did consist
of studying models that broke up the slope27 in to slope23, slope24,
etc. It did not find those models’ Model for the Variance significantly
differed from that of the Saturated Means, Unstructured Variance
model.

First things first, create piecewise time slopes for the 2nd proposed
piecewise unconditional growth curve model. Note: This will only work
with balanced data.

```
HRV_TTU_Long_AllNeedTimeSubIDs$slope26=HRV_TTU_Long_AllNeedTimeSubIDs$Time
HRV_TTU_Long_AllNeedTimeSubIDs$slope26[which(HRV_TTU_Long_AllNeedTimeSubIDs$Timepoint=="T1")]=0
HRV_TTU_Long_AllNeedTimeSubIDs$slope26[which(HRV_TTU_Long_AllNeedTimeSubIDs$Timepoint=="T2")]=0
HRV_TTU_Long_AllNeedTimeSubIDs$slope26[which(HRV_TTU_Long_AllNeedTimeSubIDs$Timepoint=="T3")]=1
HRV_TTU_Long_AllNeedTimeSubIDs$slope26[which(HRV_TTU_Long_AllNeedTimeSubIDs$Timepoint=="T4")]=2
HRV_TTU_Long_AllNeedTimeSubIDs$slope26[which(HRV_TTU_Long_AllNeedTimeSubIDs$Timepoint=="T5")]=3
HRV_TTU_Long_AllNeedTimeSubIDs$slope26[which(HRV_TTU_Long_AllNeedTimeSubIDs$Timepoint=="T6")]=4
HRV_TTU_Long_AllNeedTimeSubIDs$slope26[which(HRV_TTU_Long_AllNeedTimeSubIDs$Timepoint=="T7")]=4
HRV_TTU_Long_AllNeedTimeSubIDs$slope26[which(HRV_TTU_Long_AllNeedTimeSubIDs$Timepoint=="T8")]=4
HRV_TTU_Long_AllNeedTimeSubIDs$slope26[which(HRV_TTU_Long_AllNeedTimeSubIDs$Timepoint=="T9")]=4
HRV_TTU_Long_AllNeedTimeSubIDs$slope26[which(HRV_TTU_Long_AllNeedTimeSubIDs$Timepoint=="T10")]=4
HRV_TTU_Long_AllNeedTimeSubIDs$slope67=HRV_TTU_Long_AllNeedTimeSubIDs$Time
HRV_TTU_Long_AllNeedTimeSubIDs$slope67[which(HRV_TTU_Long_AllNeedTimeSubIDs$Timepoint=="T1")]=0
HRV_TTU_Long_AllNeedTimeSubIDs$slope67[which(HRV_TTU_Long_AllNeedTimeSubIDs$Timepoint=="T2")]=0
HRV_TTU_Long_AllNeedTimeSubIDs$slope67[which(HRV_TTU_Long_AllNeedTimeSubIDs$Timepoint=="T3")]=0
HRV_TTU_Long_AllNeedTimeSubIDs$slope67[which(HRV_TTU_Long_AllNeedTimeSubIDs$Timepoint=="T4")]=0
HRV_TTU_Long_AllNeedTimeSubIDs$slope67[which(HRV_TTU_Long_AllNeedTimeSubIDs$Timepoint=="T5")]=0
HRV_TTU_Long_AllNeedTimeSubIDs$slope67[which(HRV_TTU_Long_AllNeedTimeSubIDs$Timepoint=="T6")]=0
HRV_TTU_Long_AllNeedTimeSubIDs$slope67[which(HRV_TTU_Long_AllNeedTimeSubIDs$Timepoint=="T7")]=1
HRV_TTU_Long_AllNeedTimeSubIDs$slope67[which(HRV_TTU_Long_AllNeedTimeSubIDs$Timepoint=="T8")]=1
HRV_TTU_Long_AllNeedTimeSubIDs$slope67[which(HRV_TTU_Long_AllNeedTimeSubIDs$Timepoint=="T9")]=1
HRV_TTU_Long_AllNeedTimeSubIDs$slope67[which(HRV_TTU_Long_AllNeedTimeSubIDs$Timepoint=="T10")]=1
```

Summary of steps for this second iteration: Build a maximal model
with this new piecewise slope, run ranova and see what random effect(s)
can be dropped. Then, test the final model against model
assumptions.

The maximal model for this piecewise slope demarcation is one with
all of the piecewise slope and quadratic components for slope26, per the
results of comparing the Saturated Means, Unstructured Variance Model
with the final model in the first round of model fitting iterations.

```
ln.Fix.78.910.Rand.12.26quad.67.89.MaxMod<-lmer(data=HRV_TTU_Long_AllNeedTimeSubIDs, REML=TRUE, 
                                                formula=ln_RMSSD_ms~(1+slope12+slope26+I(slope26^2)+slope67+slope78+slope89+slope910)+
                                                (1+slope12+slope26+I(slope26^2)+slope67+slope89|New_ID), 
                                                control = lmerControl(optimizer = "bobyqa",  optCtrl=list(maxfun=5e5))) #NO CONVERGENCE OR SINGULARITY ISSUES

ranova(ln.Fix.78.910.Rand.12.26quad.67.89.MaxMod)
```

```
## Warning: Model failed to converge with 1 negative eigenvalue: -3.2e+00
```

```
## ANOVA-like table for random-effects: Single term deletions
## 
## Model:
## ln_RMSSD_ms ~ slope12 + slope26 + I(slope26^2) + slope67 + slope78 + slope89 + slope910 + (1 + slope12 + slope26 + I(slope26^2) + slope67 + slope89 | New_ID)
##                                                                                     npar
## <none>                                                                                30
## slope12 in (1 + slope12 + slope26 + I(slope26^2) + slope67 + slope89 | New_ID)        24
## slope26 in (1 + slope12 + slope26 + I(slope26^2) + slope67 + slope89 | New_ID)        24
## I(slope26^2) in (1 + slope12 + slope26 + I(slope26^2) + slope67 + slope89 | New_ID)   24
## slope67 in (1 + slope12 + slope26 + I(slope26^2) + slope67 + slope89 | New_ID)        24
## slope89 in (1 + slope12 + slope26 + I(slope26^2) + slope67 + slope89 | New_ID)        24
##                                                                                      logLik
## <none>                                                                              105.132
## slope12 in (1 + slope12 + slope26 + I(slope26^2) + slope67 + slope89 | New_ID)       71.430
## slope26 in (1 + slope12 + slope26 + I(slope26^2) + slope67 + slope89 | New_ID)       97.322
## I(slope26^2) in (1 + slope12 + slope26 + I(slope26^2) + slope67 + slope89 | New_ID)  91.346
## slope67 in (1 + slope12 + slope26 + I(slope26^2) + slope67 + slope89 | New_ID)      101.753
## slope89 in (1 + slope12 + slope26 + I(slope26^2) + slope67 + slope89 | New_ID)       93.970
##                                                                                         AIC
## <none>                                                                              -150.26
## slope12 in (1 + slope12 + slope26 + I(slope26^2) + slope67 + slope89 | New_ID)       -94.86
## slope26 in (1 + slope12 + slope26 + I(slope26^2) + slope67 + slope89 | New_ID)      -146.64
## I(slope26^2) in (1 + slope12 + slope26 + I(slope26^2) + slope67 + slope89 | New_ID) -134.69
## slope67 in (1 + slope12 + slope26 + I(slope26^2) + slope67 + slope89 | New_ID)      -155.51
## slope89 in (1 + slope12 + slope26 + I(slope26^2) + slope67 + slope89 | New_ID)      -139.94
##                                                                                        LRT
## <none>                                                                                    
## slope12 in (1 + slope12 + slope26 + I(slope26^2) + slope67 + slope89 | New_ID)      67.404
## slope26 in (1 + slope12 + slope26 + I(slope26^2) + slope67 + slope89 | New_ID)      15.621
## I(slope26^2) in (1 + slope12 + slope26 + I(slope26^2) + slope67 + slope89 | New_ID) 27.572
## slope67 in (1 + slope12 + slope26 + I(slope26^2) + slope67 + slope89 | New_ID)       6.758
## slope89 in (1 + slope12 + slope26 + I(slope26^2) + slope67 + slope89 | New_ID)      22.323
##                                                                                     Df
## <none>                                                                                
## slope12 in (1 + slope12 + slope26 + I(slope26^2) + slope67 + slope89 | New_ID)       6
## slope26 in (1 + slope12 + slope26 + I(slope26^2) + slope67 + slope89 | New_ID)       6
## I(slope26^2) in (1 + slope12 + slope26 + I(slope26^2) + slope67 + slope89 | New_ID)  6
## slope67 in (1 + slope12 + slope26 + I(slope26^2) + slope67 + slope89 | New_ID)       6
## slope89 in (1 + slope12 + slope26 + I(slope26^2) + slope67 + slope89 | New_ID)       6
##                                                                                     Pr(>Chisq)
## <none>                                                                                        
## slope12 in (1 + slope12 + slope26 + I(slope26^2) + slope67 + slope89 | New_ID)       1.392e-12
## slope26 in (1 + slope12 + slope26 + I(slope26^2) + slope67 + slope89 | New_ID)       0.0159423
## I(slope26^2) in (1 + slope12 + slope26 + I(slope26^2) + slope67 + slope89 | New_ID)  0.0001131
## slope67 in (1 + slope12 + slope26 + I(slope26^2) + slope67 + slope89 | New_ID)       0.3438028
## slope89 in (1 + slope12 + slope26 + I(slope26^2) + slope67 + slope89 | New_ID)       0.0010580
##                                                                                        
## <none>                                                                                 
## slope12 in (1 + slope12 + slope26 + I(slope26^2) + slope67 + slope89 | New_ID)      ***
## slope26 in (1 + slope12 + slope26 + I(slope26^2) + slope67 + slope89 | New_ID)      *  
## I(slope26^2) in (1 + slope12 + slope26 + I(slope26^2) + slope67 + slope89 | New_ID) ***
## slope67 in (1 + slope12 + slope26 + I(slope26^2) + slope67 + slope89 | New_ID)         
## slope89 in (1 + slope12 + slope26 + I(slope26^2) + slope67 + slope89 | New_ID)      ** 
## ---
## Signif. codes:  0 '***' 0.001 '**' 0.01 '*' 0.05 '.' 0.1 ' ' 1
```

Removing slope67 as a random effect did not lead to a significant
worse model fit. Therefore, we should drop it and refit a model
accordingly.

```
ln.Fix.67.78.910.Rand.12.26quad.89<-lmer(data=HRV_TTU_Long_AllNeedTimeSubIDs, REML=TRUE, 
                                                formula=ln_RMSSD_ms~(1+slope12+slope26+I(slope26^2)+slope67+slope78+slope89+slope910)+
                                                (1+slope12+slope26+I(slope26^2)+slope89|New_ID), 
                                                control = lmerControl(optimizer = "bobyqa",  optCtrl=list(maxfun=5e5))) #NO CONVERGENCE OR SINGULARITY ISSUES

ranova(ln.Fix.67.78.910.Rand.12.26quad.89)
```

```
## ANOVA-like table for random-effects: Single term deletions
## 
## Model:
## ln_RMSSD_ms ~ slope12 + slope26 + I(slope26^2) + slope67 + slope78 + slope89 + slope910 + (1 + slope12 + slope26 + I(slope26^2) + slope89 | New_ID)
##                                                                           npar
## <none>                                                                      24
## slope12 in (1 + slope12 + slope26 + I(slope26^2) + slope89 | New_ID)        19
## slope26 in (1 + slope12 + slope26 + I(slope26^2) + slope89 | New_ID)        19
## I(slope26^2) in (1 + slope12 + slope26 + I(slope26^2) + slope89 | New_ID)   19
## slope89 in (1 + slope12 + slope26 + I(slope26^2) + slope89 | New_ID)        19
##                                                                            logLik
## <none>                                                                    101.753
## slope12 in (1 + slope12 + slope26 + I(slope26^2) + slope89 | New_ID)       69.289
## slope26 in (1 + slope12 + slope26 + I(slope26^2) + slope89 | New_ID)       95.661
## I(slope26^2) in (1 + slope12 + slope26 + I(slope26^2) + slope89 | New_ID)  88.782
## slope89 in (1 + slope12 + slope26 + I(slope26^2) + slope89 | New_ID)       92.936
##                                                                               AIC
## <none>                                                                    -155.51
## slope12 in (1 + slope12 + slope26 + I(slope26^2) + slope89 | New_ID)      -100.58
## slope26 in (1 + slope12 + slope26 + I(slope26^2) + slope89 | New_ID)      -153.32
## I(slope26^2) in (1 + slope12 + slope26 + I(slope26^2) + slope89 | New_ID) -139.56
## slope89 in (1 + slope12 + slope26 + I(slope26^2) + slope89 | New_ID)      -147.87
##                                                                              LRT
## <none>                                                                          
## slope12 in (1 + slope12 + slope26 + I(slope26^2) + slope89 | New_ID)      64.927
## slope26 in (1 + slope12 + slope26 + I(slope26^2) + slope89 | New_ID)      12.184
## I(slope26^2) in (1 + slope12 + slope26 + I(slope26^2) + slope89 | New_ID) 25.941
## slope89 in (1 + slope12 + slope26 + I(slope26^2) + slope89 | New_ID)      17.633
##                                                                           Df
## <none>                                                                      
## slope12 in (1 + slope12 + slope26 + I(slope26^2) + slope89 | New_ID)       5
## slope26 in (1 + slope12 + slope26 + I(slope26^2) + slope89 | New_ID)       5
## I(slope26^2) in (1 + slope12 + slope26 + I(slope26^2) + slope89 | New_ID)  5
## slope89 in (1 + slope12 + slope26 + I(slope26^2) + slope89 | New_ID)       5
##                                                                           Pr(>Chisq)
## <none>                                                                              
## slope12 in (1 + slope12 + slope26 + I(slope26^2) + slope89 | New_ID)       1.161e-12
## slope26 in (1 + slope12 + slope26 + I(slope26^2) + slope89 | New_ID)        0.032358
## I(slope26^2) in (1 + slope12 + slope26 + I(slope26^2) + slope89 | New_ID)  9.163e-05
## slope89 in (1 + slope12 + slope26 + I(slope26^2) + slope89 | New_ID)        0.003444
##                                                                              
## <none>                                                                       
## slope12 in (1 + slope12 + slope26 + I(slope26^2) + slope89 | New_ID)      ***
## slope26 in (1 + slope12 + slope26 + I(slope26^2) + slope89 | New_ID)      *  
## I(slope26^2) in (1 + slope12 + slope26 + I(slope26^2) + slope89 | New_ID) ***
## slope89 in (1 + slope12 + slope26 + I(slope26^2) + slope89 | New_ID)      ** 
## ---
## Signif. codes:  0 '***' 0.001 '**' 0.01 '*' 0.05 '.' 0.1 ' ' 1
```

Removing any other slopes as random effects leads to significant
worse fit, so retain all.

## Summary of the selected unconditional growth curve model - Second Iteration

```
summary(ln.Fix.67.78.910.Rand.12.26quad.89, ddf="Satterthwaite")
```

```
## Linear mixed model fit by REML. t-tests use Satterthwaite's method [
## lmerModLmerTest]
## Formula: ln_RMSSD_ms ~ (1 + slope12 + slope26 + I(slope26^2) + slope67 +  
##     slope78 + slope89 + slope910) + (1 + slope12 + slope26 +  
##     I(slope26^2) + slope89 | New_ID)
##    Data: HRV_TTU_Long_AllNeedTimeSubIDs
## Control: lmerControl(optimizer = "bobyqa", optCtrl = list(maxfun = 5e+05))
## 
## REML criterion at convergence: -203.5
## 
## Scaled residuals: 
##     Min      1Q  Median      3Q     Max 
## -2.3611 -0.5824  0.0570  0.4909  2.6899 
## 
## Random effects:
##  Groups   Name         Variance  Std.Dev. Corr                   
##  New_ID   (Intercept)  0.1965289 0.44332                         
##           slope12      0.0681159 0.26099  -0.25                  
##           slope26      0.0055503 0.07450  -0.71  0.68            
##           I(slope26^2) 0.0006528 0.02555   0.39 -0.71 -0.92      
##           slope89      0.0145427 0.12059   0.22  0.06  0.07 -0.28
##  Residual              0.0104706 0.10233                         
## Number of obs: 310, groups:  New_ID, 31
## 
## Fixed effects:
##                Estimate Std. Error         df t value Pr(>|t|)    
## (Intercept)    3.300604   0.081715  30.000855  40.391  < 2e-16 ***
## slope12        0.064481   0.053237  30.515256   1.211  0.23511    
## slope26       -0.008234   0.024471  51.517048  -0.336  0.73786    
## I(slope26^2)   0.005056   0.006722  43.282325   0.752  0.45605    
## slope67        0.072369   0.025237 181.999995   2.868  0.00463 ** 
## slope78        0.006340   0.025991 181.999995   0.244  0.80754    
## slope89        0.046729   0.033833  67.478537   1.381  0.17178    
## slope910       0.030553   0.025991 181.999996   1.176  0.24131    
## ---
## Signif. codes:  0 '***' 0.001 '**' 0.01 '*' 0.05 '.' 0.1 ' ' 1
## 
## Correlation of Fixed Effects:
##             (Intr) slop12 slop26 I(26^2 slop67 slop78 slop89
## slope12     -0.295                                          
## slope26     -0.380  0.130                                   
## I(slop26^2)  0.261 -0.292 -0.931                            
## slope67      0.000 -0.022  0.203 -0.284                     
## slope78      0.000  0.000  0.000  0.000 -0.515              
## slope89      0.136  0.032  0.025 -0.122  0.000 -0.384       
## slope910     0.000  0.000  0.000  0.000  0.000  0.000 -0.384
```

slope67 is the only time slope with a significant fixed effect.

### Fixed Effects

```
fixef(ln.Fix.67.78.910.Rand.12.26quad.89);length(fixef(ln.Fix.67.78.910.Rand.12.26quad.89))
```

```
##  (Intercept)      slope12      slope26 I(slope26^2)      slope67      slope78 
##  3.300604483  0.064481447 -0.008234467  0.005055627  0.072368699  0.006340490 
##      slope89     slope910 
##  0.046729394  0.030553171
```

```
## [1] 8
```

### Compute and store conditional means for each time period from values of time predictors

```
CondMeans26slope<-contest1D(ln.Fix.67.78.910.Rand.12.26quad.89, ddf="Satterthwaite",  L=c(1,0,0,0,0,0,0,0));CondMeans26slope
```

```
##   Estimate Std. Error       df  t value     Pr(>|t|)
## 1 3.300604 0.08171537 30.00086 40.39148 1.028785e-27
```

```
CondMeans26slope<-rbind(CondMeans26slope,contest1D(ln.Fix.67.78.910.Rand.12.26quad.89, ddf="Satterthwaite", L=c(1,1,0,0,0,0,0,0)))
CondMeans26slope<-rbind(CondMeans26slope,contest1D(ln.Fix.67.78.910.Rand.12.26quad.89, ddf="Satterthwaite", L=c(1,1,1,1,0,0,0,0)))
CondMeans26slope<-rbind(CondMeans26slope,contest1D(ln.Fix.67.78.910.Rand.12.26quad.89, ddf="Satterthwaite", L=c(1,1,2,4,0,0,0,0)))
CondMeans26slope<-rbind(CondMeans26slope,contest1D(ln.Fix.67.78.910.Rand.12.26quad.89, ddf="Satterthwaite", L=c(1,1,3,9,0,0,0,0)))
CondMeans26slope<-rbind(CondMeans26slope,contest1D(ln.Fix.67.78.910.Rand.12.26quad.89, ddf="Satterthwaite", L=c(1,1,4,16,0,0,0,0)))
CondMeans26slope<-rbind(CondMeans26slope,contest1D(ln.Fix.67.78.910.Rand.12.26quad.89, ddf="Satterthwaite", L=c(1,1,4,16,1,0,0,0)))
CondMeans26slope<-rbind(CondMeans26slope,contest1D(ln.Fix.67.78.910.Rand.12.26quad.89, ddf="Satterthwaite", L=c(1,1,4,16,1,1,0,0)))
CondMeans26slope<-rbind(CondMeans26slope,contest1D(ln.Fix.67.78.910.Rand.12.26quad.89, ddf="Satterthwaite", L=c(1,1,4,16,1,1,1,0)))
CondMeans26slope<-rbind(CondMeans26slope,contest1D(ln.Fix.67.78.910.Rand.12.26quad.89, ddf="Satterthwaite", L=c(1,1,4,16,1,1,1,1)))

CondMeans26slope["Timepoint"]<-c("T1","T2","T3","T4","T5","T6","T7","T8","T9","T10")
CondMeans26slope["Model"]<-c("Random slope12, slope26, slope26^2, slope89; Fix slope67, slope78, slope910 Unconditional Growth Curve Model")
CondMeans26slope_df<-subset.data.frame(CondMeans26slope, select=c("Model","Timepoint","Estimate","Std. Error","df"))
colnames(CondMeans26slope_df)<-c("Model", "Timepoint","Estimate","SE","df")
CondMeans<-rbind(CondMeans,CondMeans26slope_df)
CondMeans$Model<-factor(CondMeans$Model, levels = c("Saturated Means and Unstructured Variance Model","Random slope12, slope27, slope89; Fixed slope27^2, slope78, slope910 Unconditional Growth Curve Model (Original model based on experimental setup/theoretical rationale)","Random slope12, slope26, slope26^2, slope89; Fix slope67, slope78, slope910 Unconditional Growth Curve Model"))
```

##

## Model Comparison and Assessment - Second Iteration

### Need to compare the second iteration unconditional growth curve model against the first iteration’s and the Saturated Means, Unstructured Variance Model aka the “Total Answer Key”

First, visually compare the estimated marginal means of each
model.

```
graph_3ModComps_lnRMSSD<-ggplot(CondMeans, aes(x=Timepoint, y=Estimate, group=Model)) + 
                                  geom_line(aes(colour=Model, linetype=Model, linewidth=Model)) + 
                                    scale_colour_manual(labels = ~ stringr::str_wrap(.x, width = 35),values=c("black","darkgray","gray55")) + 
                                    scale_linetype_manual(labels = ~ stringr::str_wrap(.x, width = 35),values=c("longdash","dotdash","solid")) + 
                                    scale_discrete_manual("linewidth",labels = ~ stringr::str_wrap(.x, width = 35),values=c(5,3,3)) + 
                                  geom_point(aes(colour=Model,shape=Model,size=Model)) + 
                                    scale_colour_manual(labels = ~ stringr::str_wrap(.x, width = 35),values=c("black","darkgray","gray55")) + 
                                    scale_shape_manual(labels = ~ stringr::str_wrap(.x, width = 35),values=c(19,15,17)) + 
                                    scale_size_manual(labels = ~ stringr::str_wrap(.x, width = 35),values=c(10,6,6)) + 
                                  ggtitle("Comparing Models of Means") +
                                  labs(y="Estimated ln(RMSSD)", x="Time period") + 
                                  theme(axis.ticks.x=element_blank(),
                                    legend.position = "right", 
                                    legend.key.width = unit(2, 'cm'),
                                    legend.key = element_blank(),
                                    legend.text=element_text(margin = margin(t = 20),size=30),
                                    #plot.title = element_text(hjust = 0.5),
                                    panel.grid.major = element_blank(),
                                    panel.grid.minor = element_blank(),
                                    panel.background = element_blank(),
                                    axis.line.x = element_line(colour = "black"),
                                    axis.line.y = element_line(colour = "black"),
                                    text=element_text(color="black", size=50),
                                    axis.text=element_text(color="black", size=40),
                                    strip.background = element_rect(color = "black",fill = "white"))
graph_3ModComps_lnRMSSD
```

Summary of the plot: This plot shows how the Model for the Means of the
revised unconditional growth curve model aligns much better with the
Model for the Means of the Saturated Means, Unstructured Variance
model.

### Test absolute fit of this unconditional growth curve model’s Model of the Means by saturating it and then seeing if this is a significantly better fit than the unconditional growth curve model itself.

To saturate, add T5 time point dummy codes to time points that are
not explicitly part of any of the slopes and we need to saturate the
means of this model in order to compare this model’s model for the means
with that of the saturated means, unstructured variance model.

We must do this because this is a piecewise model. We do not need to
do this for the other types of models.

```
Saturated.ln.Fix.67.78.910.Rand.12.26quad.89<-lmer(data=HRV_TTU_Long_AllNeedTimeSubIDs, REML=TRUE, 
                                                    formula=ln_RMSSD_ms~(1+slope12+slope26+T4+I(slope26^2)+T5+slope67+slope78+slope89+slope910)+
                                                         (1+slope12+slope26+I(slope26^2)+slope89|New_ID), 
                                                          control=lmerControl(optimizer ="bobyqa", optCtrl=list(maxfun=5e5)))
```

Show results of this saturated unconditional growth curve model using
Satterthwaite DDF including -2LL as deviance.

```
summary(Saturated.ln.Fix.67.78.910.Rand.12.26quad.89, ddf="Satterthwaite"); llikAIC(Saturated.ln.Fix.67.78.910.Rand.12.26quad.89, chkREML=FALSE)
```

```
## Linear mixed model fit by REML. t-tests use Satterthwaite's method [
## lmerModLmerTest]
## Formula: ln_RMSSD_ms ~ (1 + slope12 + slope26 + T4 + I(slope26^2) + T5 +  
##     slope67 + slope78 + slope89 + slope910) + (1 + slope12 +  
##     slope26 + I(slope26^2) + slope89 | New_ID)
##    Data: HRV_TTU_Long_AllNeedTimeSubIDs
## Control: lmerControl(optimizer = "bobyqa", optCtrl = list(maxfun = 5e+05))
## 
## REML criterion at convergence: -192.9
## 
## Scaled residuals: 
##      Min       1Q   Median       3Q      Max 
## -2.34919 -0.59118  0.05739  0.49082  2.68148 
## 
## Random effects:
##  Groups   Name         Variance  Std.Dev. Corr                   
##  New_ID   (Intercept)  0.1964230 0.44320                         
##           slope12      0.0680060 0.26078  -0.25                  
##           slope26      0.0055431 0.07445  -0.71  0.68            
##           I(slope26^2) 0.0006516 0.02553   0.39 -0.71 -0.92      
##           slope89      0.0144551 0.12023   0.22  0.06  0.07 -0.28
##  Residual              0.0105784 0.10285                         
## Number of obs: 310, groups:  New_ID, 31
## 
## Fixed effects:
##                Estimate Std. Error         df t value Pr(>|t|)    
## (Intercept)   3.301e+00  8.172e-02  3.000e+01  40.391  < 2e-16 ***
## slope12       6.263e-02  5.363e-02  3.136e+01   1.168  0.25167    
## slope26       9.125e-04  3.635e-02  1.332e+02   0.025  0.98001    
## T4           -1.222e-02  3.229e-02  1.800e+02  -0.378  0.70558    
## I(slope26^2)  2.909e-03  9.090e-03  1.072e+02   0.320  0.74956    
## T5           -5.613e-03  2.921e-02  1.800e+02  -0.192  0.84783    
## slope67       7.197e-02  2.612e-02  1.800e+02   2.755  0.00647 ** 
## slope78       6.340e-03  2.612e-02  1.800e+02   0.243  0.80851    
## slope89       4.673e-02  3.389e-02  6.787e+01   1.379  0.17251    
## slope910      3.055e-02  2.612e-02  1.800e+02   1.170  0.24374    
## ---
## Signif. codes:  0 '***' 0.001 '**' 0.01 '*' 0.05 '.' 0.1 ' ' 1
## 
## Correlation of Fixed Effects:
##             (Intr) slop12 slop26 T4     I(26^2 T5     slop67 slop78 slop89
## slope12     -0.293                                                        
## slope26     -0.255  0.001                                                 
## T4           0.000  0.099 -0.695                                          
## I(slop26^2)  0.193 -0.138 -0.960  0.646                                   
## T5           0.000  0.109 -0.616  0.603  0.536                            
## slope67      0.000  0.000  0.030  0.067 -0.120  0.224                     
## slope78      0.000  0.000  0.000  0.000  0.000  0.000 -0.500              
## slope89      0.136  0.032  0.017  0.000 -0.090  0.000  0.000 -0.385       
## slope910     0.000  0.000  0.000  0.000  0.000  0.000  0.000  0.000 -0.385
```

```
## $logLik
## 'log Lik.' 96.46632 (df=26)
## 
## $AICtab
##        AIC        BIC     logLik   deviance   df.resid 
## -140.93264  -43.78176   96.46632 -192.93264  284.00000
```

```
fixef(Saturated.ln.Fix.67.78.910.Rand.12.26quad.89);length(fixef(Saturated.ln.Fix.67.78.910.Rand.12.26quad.89))
```

```
##   (Intercept)       slope12       slope26            T4  I(slope26^2) 
##  3.3006044829  0.0626323231  0.0009125329 -0.0122187193  0.0029091925 
##            T5       slope67       slope78       slope89      slope910 
## -0.0056126389  0.0719727679  0.0063404904  0.0467293939  0.0305531714
```

```
## [1] 10
```

Does saturating ln.Fix.67.78.910.Rand.12.26quad.89 lead to a
significantly better model than the unconditional growth curve model
without it?

```
contestMD(Saturated.ln.Fix.67.78.910.Rand.12.26quad.89, ddf="Satterthwaite", L=rbind(c(0,0,0,1,0,0,0,0,0,0),c(0,0,0,0,0,1,0,0,0,0)))
```

```
##        Sum Sq      Mean Sq NumDF    DenDF    F value    Pr(>F)
## 1 0.001536257 0.0007681283     2 179.9876 0.07261303 0.9299879
```

F(2,179.999)=0.07261304, p=0.9299878, suggesting that saturating this
unconditional growth curve model did not lead to a significant better
model fit, so the Model for the Means of this unconditional growth curve
model is (statistically) sufficient.

Now we will assess if the Model for the Variance of this
Unconditional Growth Curve Model is significantly different than the
Model for the Variance of the Saturated Means, Unstructured Variance
Model. Just as before, we keep the fixed effect of this growth curve
model constant, but we change the random effects to be Unstructured
Variance as this is what the Model for the Variance is in the Saturated
Means, Unstructured Variance model.

Logistical detail: Need to use lme instead of lmer as the former
allows the variance structure to be more specifically specified. That is
why both models are rebuilt in lme. We are also using REML in GLS for
accurate model fitting.

```
lnSameFix5UnVarREML = gls(data=HRV_TTU_Long_AllNeedTimeSubIDs, method="REML", 
                         model=ln_RMSSD_ms~1+slope12+slope26+I(slope26^2)+slope67+slope78+slope89+slope910, 
                         correlation=corSymm(form=~as.numeric(Timepoint)|New_ID), # Unstructured correlations
                         weights=varIdent(form=~1|(Timepoint)))                   # Heterogeneous variances

lmeREML.ln.Fix.67.78.910.Rand.12.26quad.89<-lme(data=HRV_TTU_Long_AllNeedTimeSubIDs, method="REML", 
                                            ln_RMSSD_ms~1+slope12+slope26+I(slope26^2)+slope67+slope78+slope89+slope910,
                                              random=~1+slope12+slope26+I(slope26^2)+slope89|New_ID,
                                              control=lmeControl(opt = "optim", optimMethod = "BFGS", msMaxIter=2000, msMaxEval=2000))
anova(lnSameFix5UnVarREML, lmeREML.ln.Fix.67.78.910.Rand.12.26quad.89)
```

```
##                                            Model df       AIC       BIC
## lnSameFix5UnVarREML                            1 63 -128.2904 105.46645
## lmeREML.ln.Fix.67.78.910.Rand.12.26quad.89     2 24 -155.4363 -66.38601
##                                              logLik   Test  L.Ratio p-value
## lnSameFix5UnVarREML                        127.1452                        
## lmeREML.ln.Fix.67.78.910.Rand.12.26quad.89 101.7181 1 vs 2 50.85419  0.0968
```

-2LL=46.41498, p=0.1932, suggesting the Model for the Variance of
this unconditional growth curve model is *not* significantly
different than the one of the Saturated Means, Unstructured Variance
model.

Visually compare each unconditional growth curve model’s Model for
the Variance with that of the Saturated Means, Unstructured Variance
model. Need to first build a combined data frame in order to plot
effectively.

```
TotalVar3Mods<-data.frame(Model=character(), Timepoint=character(), TotalVar=numeric())
TotalVar3Mods[1:10,"Model"]<-"Saturated Means and Unstructured Variance Model"
TotalVar3Mods[11:20,"Model"]<-"Random slope12, slope27, slope89; Fixed slope27^2, slope78, slope910 Unconditional Growth Curve Model (Original model based on experimental setup/theoretical rationale)"
TotalVar3Mods[21:30,"Model"]<-"Random slope12, slope26, slope26^2, slope89; Fix slope67, slope78, slope910 Unconditional Growth Curve Model"
TotalVar3Mods$Model<-factor(TotalVar3Mods$Model, levels = c("Saturated Means and Unstructured Variance Model", "Random slope12, slope27, slope89; Fixed slope27^2, slope78, slope910 Unconditional Growth Curve Model (Original model based on experimental setup/theoretical rationale)","Random slope12, slope26, slope26^2, slope89; Fix slope67, slope78, slope910 Unconditional Growth Curve Model"))  

TotalVar3Mods[1:10,"Timepoint"]<-c("T1","T2","T3","T4","T5","T6","T7","T8","T9","T10")
TotalVar3Mods[11:20,"Timepoint"]<-c("T1","T2","T3","T4","T5","T6","T7","T8","T9","T10")
TotalVar3Mods[21:30,"Timepoint"]<-c("T1","T2","T3","T4","T5","T6","T7","T8","T9","T10")
TotalVar3Mods$Timepoint<-factor(TotalVar3Mods$Timepoint, levels = c("T1","T2","T3","T4","T5","T6","T7","T8","T9","T10"))  

TotalVar3Mods[1:10,"TotalVar"]<-(diag(getVarCov(lnSatUN, New_ID="6", type="marginal")))
TotalVar3Mods[11:20,"TotalVar"]<-(diag(getVarCov(lmeREML.ln.Fix910.27quad.78.Rand.12.27lin.89, New_ID="6", type="marginal")[[1]]))
TotalVar3Mods[21:30,"TotalVar"]<-(diag(getVarCov(lmeREML.ln.Fix.67.78.910.Rand.12.26quad.89, New_ID="6", type="marginal")[[1]]))

row.names(TotalVar3Mods)<-1:nrow(TotalVar3Mods)
```

```
graph_CompTotVar3Mods_lnRMSSD<-ggplot(TotalVar3Mods, aes(x=Timepoint, y=TotalVar, group=Model)) + 
                                    geom_line(aes(colour=Model, linetype=Model, linewidth=Model)) + 
                                      scale_colour_manual(labels = ~ stringr::str_wrap(.x, width = 35),values=c("black","darkgray","gray55")) + 
                                      scale_linetype_manual(labels = ~ stringr::str_wrap(.x, width = 35),values=c("longdash","dotdash","solid")) + 
                                      scale_discrete_manual("linewidth",labels = ~ stringr::str_wrap(.x, width = 35),values=c(5,3,3)) + 
                                  geom_point(aes(colour=Model,shape=Model,size=Model)) + 
                                      scale_colour_manual(labels = ~ stringr::str_wrap(.x, width = 35),values=c("black","darkgray","gray55")) + 
                                      scale_shape_manual(labels = ~ stringr::str_wrap(.x, width = 35),values=c(19,15,17)) + 
                                      scale_size_manual(labels = ~ stringr::str_wrap(.x, width = 35),values=c(10,6,6)) + 
                                  ggtitle("Comparing Models of the Variance") +
                                    labs(y="Estimated variance [ln(RMSSD)]", x="Time period") + 
                                    theme(axis.ticks.x=element_blank(),
                                      legend.position = "right", 
                                      legend.key.width = unit(2, 'cm'), 
                                      legend.key = element_blank(),
                                      legend.text=element_text(margin = margin(t = 20),size=30),
                                      #plot.title = element_text(hjust = 0.5),
                                      panel.grid.major = element_blank(),
                                      panel.grid.minor = element_blank(),
                                      panel.background = element_blank(),
                                      axis.line.x = element_line(colour = "black"),
                                      axis.line.y = element_line(colour = "black"),
                                      text=element_text(color="black", size=50),
                                      axis.text=element_text(color="black", size=40),
                                      strip.background = element_rect(color = "black",fill = "white"))
graph_CompTotVar3Mods_lnRMSSD
```

Summary: The Model for the Variance of the revised unconditional growth
curve model may not be significantly different statistically, but it
seems to still differ greatly, especially for time period 4 - time
period 8.

Even though this revised model fits the bill, i.e., it converges, is
non-singular, its Model for the Means and Model for the Variance does
not significantly differ from the Saturated Means, Unstructured Variance
model, who is to say that the way we broke up these slopes is really
best…what if slope57 needs its own slope and slope25^2 (random or fixed
or a combo of the two) is actually a better model fit? This is what we
are going to look in to now.

## Unconditional growth curve modeling - Third Iteration

Create piecewise time slopes for the 3rd iteration of the piecewise
unconditional growth curve model (i.e., see how an unconditional growth
curve model with slope25 and slope57 compares).

```
HRV_TTU_Long_AllNeedTimeSubIDs$slope25=HRV_TTU_Long_AllNeedTimeSubIDs$Time
HRV_TTU_Long_AllNeedTimeSubIDs$slope25[which(HRV_TTU_Long_AllNeedTimeSubIDs$Timepoint=="T1")]=0
HRV_TTU_Long_AllNeedTimeSubIDs$slope25[which(HRV_TTU_Long_AllNeedTimeSubIDs$Timepoint=="T2")]=0
HRV_TTU_Long_AllNeedTimeSubIDs$slope25[which(HRV_TTU_Long_AllNeedTimeSubIDs$Timepoint=="T3")]=1
HRV_TTU_Long_AllNeedTimeSubIDs$slope25[which(HRV_TTU_Long_AllNeedTimeSubIDs$Timepoint=="T4")]=2
HRV_TTU_Long_AllNeedTimeSubIDs$slope25[which(HRV_TTU_Long_AllNeedTimeSubIDs$Timepoint=="T5")]=3
HRV_TTU_Long_AllNeedTimeSubIDs$slope25[which(HRV_TTU_Long_AllNeedTimeSubIDs$Timepoint=="T6")]=3
HRV_TTU_Long_AllNeedTimeSubIDs$slope25[which(HRV_TTU_Long_AllNeedTimeSubIDs$Timepoint=="T7")]=3
HRV_TTU_Long_AllNeedTimeSubIDs$slope25[which(HRV_TTU_Long_AllNeedTimeSubIDs$Timepoint=="T8")]=3
HRV_TTU_Long_AllNeedTimeSubIDs$slope25[which(HRV_TTU_Long_AllNeedTimeSubIDs$Timepoint=="T9")]=3
HRV_TTU_Long_AllNeedTimeSubIDs$slope25[which(HRV_TTU_Long_AllNeedTimeSubIDs$Timepoint=="T10")]=3
HRV_TTU_Long_AllNeedTimeSubIDs$slope57=HRV_TTU_Long_AllNeedTimeSubIDs$Time
HRV_TTU_Long_AllNeedTimeSubIDs$slope57[which(HRV_TTU_Long_AllNeedTimeSubIDs$Timepoint=="T1")]=0
HRV_TTU_Long_AllNeedTimeSubIDs$slope57[which(HRV_TTU_Long_AllNeedTimeSubIDs$Timepoint=="T2")]=0
HRV_TTU_Long_AllNeedTimeSubIDs$slope57[which(HRV_TTU_Long_AllNeedTimeSubIDs$Timepoint=="T3")]=0
HRV_TTU_Long_AllNeedTimeSubIDs$slope57[which(HRV_TTU_Long_AllNeedTimeSubIDs$Timepoint=="T4")]=0
HRV_TTU_Long_AllNeedTimeSubIDs$slope57[which(HRV_TTU_Long_AllNeedTimeSubIDs$Timepoint=="T5")]=0
HRV_TTU_Long_AllNeedTimeSubIDs$slope57[which(HRV_TTU_Long_AllNeedTimeSubIDs$Timepoint=="T6")]=1
HRV_TTU_Long_AllNeedTimeSubIDs$slope57[which(HRV_TTU_Long_AllNeedTimeSubIDs$Timepoint=="T7")]=2
HRV_TTU_Long_AllNeedTimeSubIDs$slope57[which(HRV_TTU_Long_AllNeedTimeSubIDs$Timepoint=="T8")]=2
HRV_TTU_Long_AllNeedTimeSubIDs$slope57[which(HRV_TTU_Long_AllNeedTimeSubIDs$Timepoint=="T9")]=2
HRV_TTU_Long_AllNeedTimeSubIDs$slope57[which(HRV_TTU_Long_AllNeedTimeSubIDs$Timepoint=="T10")]=2
```

Summary of modeling steps: Build the maximal model with slope25 and
slope57 (and all other slopes) and then run rANOVA tests to see what
random effect(s) can be dropped. All singular and non converging models
are not considered for comparison.

```
ln.Fix.78.910.Rand.12.25quad.57quad.89.MaxMod<-lmer(data=HRV_TTU_Long_AllNeedTimeSubIDs, REML=TRUE, 
                                                formula=ln_RMSSD_ms~(1+slope12+slope25+I(slope25^2)+slope57+I(slope57^2)+slope78+slope89+slope910)+
                                                (1+slope12+slope25+I(slope25^2)+slope57+I(slope57^2)+slope89|New_ID), 
                                                control = lmerControl(optimizer = "bobyqa", optCtrl=list(maxfun=5e5))) #singular, DO NOT USE!
```

```
## Warning: Model failed to converge with 1 negative eigenvalue: -5.1e-04
```

```
allFit(ln.Fix.78.910.Rand.12.25quad.57quad.89.MaxMod) #differences in negative log-likelihoods: max=7.3; std dev=2.92
```

```
## bobyqa :
```

```
## Warning: Model failed to converge with 1 negative eigenvalue: -5.1e-04
```

```
## [OK]
## Nelder_Mead :
```

```
## Warning in checkConv(attr(opt, "derivs"), opt$par, ctrl = control$checkConv, :
## unable to evaluate scaled gradient
```

```
## Warning in checkConv(attr(opt, "derivs"), opt$par, ctrl = control$checkConv, :
## Model failed to converge: degenerate Hessian with 1 negative eigenvalues
```

```
## Warning: Model failed to converge with 1 negative eigenvalue: -6.6e-01
```

```
## [OK]
## nlminbwrap :
```

```
## Warning in optwrap(optimizer, devfun, getStart(start, rho$pp), lower =
## rho$lower, : convergence code 1 from nlminbwrap: iteration limit reached without
## convergence (10)
```

```
## Warning: Model failed to converge with 1 negative eigenvalue: -2.0e-02
```

```
## [OK]
## nmkbw :
```

```
## Warning in ctrl[namc] <- control: number of items to replace is not a multiple
## of replacement length
```

```
## Warning in checkConv(attr(opt, "derivs"), opt$par, ctrl = control$checkConv, :
## unable to evaluate scaled gradient
```

```
## Warning in checkConv(attr(opt, "derivs"), opt$par, ctrl = control$checkConv, :
## Model failed to converge: degenerate Hessian with 1 negative eigenvalues
```

```
## Warning: Model failed to converge with 1 negative eigenvalue: -1.1e-01
```

```
## [OK]
## optimx.L-BFGS-B :
```

```
## Warning: Model failed to converge with 1 negative eigenvalue: -9.0e-02
```

```
## [OK]
## nloptwrap.NLOPT_LN_NELDERMEAD :
```

```
## Warning in checkConv(attr(opt, "derivs"), opt$par, ctrl = control$checkConv, :
## Model failed to converge with max|grad| = 1.43434 (tol = 0.002, component 1)
```

```
## [OK]
## nloptwrap.NLOPT_LN_BOBYQA :
```

```
## Warning: Model failed to converge with 2 negative eigenvalues: -7.5e-03 -4.4e-02
```

```
## [OK]
```

```
## original model:
## ln_RMSSD_ms ~ (1 + slope12 + slope25 + I(slope25^2) + slope57 + I(slope57^2) ... 
## data:  HRV_TTU_Long_AllNeedTimeSubIDs 
## optimizers (7): bobyqa, Nelder_Mead, nlminbwrap, nmkbw, optimx.L-BFGS-B,nloptwrap.NLOPT_LN_N...
## differences in negative log-likelihoods:
## max= 7.3 ; std dev= 2.73
```

```
ln.Fix.78.910.57quad.Rand.12.25quad.57lin.89<-lmer(data=HRV_TTU_Long_AllNeedTimeSubIDs, REML=TRUE, 
                                                formula=ln_RMSSD_ms~(1+slope12+slope25+I(slope25^2)+slope57+I(slope57^2)+slope78+slope89+slope910)+
                                                (1+slope12+slope25+I(slope25^2)+slope57+slope89|New_ID), 
                                                control = lmerControl(optimizer = "bobyqa", optCtrl=list(maxfun=5e5))) #singular, DO NOT USE!
```

```
## Warning: Model failed to converge with 1 negative eigenvalue: -1.9e-02
```

```
allFit(ln.Fix.78.910.57quad.Rand.12.25quad.57lin.89) #differences in negative log-likelihoods: max=10.9; std dev=4.41
```

```
## bobyqa :
```

```
## Warning: Model failed to converge with 1 negative eigenvalue: -1.9e-02
```

```
## [OK]
## Nelder_Mead :
```

```
## Warning in checkConv(attr(opt, "derivs"), opt$par, ctrl = control$checkConv, :
## Model failed to converge with max|grad| = 5.89211 (tol = 0.002, component 1)
```

```
## [OK]
## nlminbwrap :
```

```
## Warning in optwrap(optimizer, devfun, getStart(start, rho$pp), lower =
## rho$lower, : convergence code 1 from nlminbwrap: iteration limit reached without
## convergence (10)
```

```
## Warning in optwrap(optimizer, devfun, opt$par, lower = rho$lower, control =
## control, : convergence code 1 from nlminbwrap: iteration limit reached without
## convergence (10)
```

```
## Warning in checkConv(attr(opt, "derivs"), opt$par, ctrl = control$checkConv, :
## unable to evaluate scaled gradient
```

```
## Warning in checkConv(attr(opt, "derivs"), opt$par, ctrl = control$checkConv, :
## Model failed to converge: degenerate Hessian with 2 negative eigenvalues
```

```
## Warning: Model failed to converge with 2 negative eigenvalues: -8.2e-01 -9.8e-01
```

```
## [OK]
## nmkbw :
```

```
## Warning in ctrl[namc] <- control: number of items to replace is not a multiple
## of replacement length
```

```
## Warning: Model failed to converge with 1 negative eigenvalue: -8.6e-02
```

```
## [OK]
## optimx.L-BFGS-B :
```

```
## Warning: Model failed to converge with 1 negative eigenvalue: -2.4e-02
```

```
## [OK]
## nloptwrap.NLOPT_LN_NELDERMEAD :
```

```
## Warning in checkConv(attr(opt, "derivs"), opt$par, ctrl = control$checkConv, :
## unable to evaluate scaled gradient
```

```
## Warning in checkConv(attr(opt, "derivs"), opt$par, ctrl = control$checkConv, :
## Model failed to converge: degenerate Hessian with 1 negative eigenvalues
```

```
## Warning: Model failed to converge with 1 negative eigenvalue: -1.6e-01
```

```
## [OK]
## nloptwrap.NLOPT_LN_BOBYQA :
```

```
## Warning in checkConv(attr(opt, "derivs"), opt$par, ctrl = control$checkConv, :
## Model failed to converge with max|grad| = 0.100655 (tol = 0.002, component 1)
```

```
## [OK]
```

```
## original model:
## ln_RMSSD_ms ~ (1 + slope12 + slope25 + I(slope25^2) + slope57 + I(slope57^2) ... 
## data:  HRV_TTU_Long_AllNeedTimeSubIDs 
## optimizers (7): bobyqa, Nelder_Mead, nlminbwrap, nmkbw, optimx.L-BFGS-B,nloptwrap.NLOPT_LN_N...
## differences in negative log-likelihoods:
## max= 10.9 ; std dev= 4.08
```

```
ln.Fix.78.910.25quad.Rand.12.25lin.57quad.89<-lmer(data=HRV_TTU_Long_AllNeedTimeSubIDs, REML=TRUE, 
                                                formula=ln_RMSSD_ms~(1+slope12+slope25+I(slope25^2)+slope57+I(slope57^2)+slope78+slope89+slope910)+
                                                (1+slope12+slope25+slope57+I(slope57^2)+slope89|New_ID), 
                                                control = lmerControl(optimizer = "bobyqa", optCtrl=list(maxfun=5e5))) #singular, DO NOT USE!
allFit(ln.Fix.78.910.25quad.Rand.12.25lin.57quad.89) #differences in negative log-likelihoods: max=6.07; std dev=2.41
```

```
## bobyqa : [OK]
## Nelder_Mead :
```

```
## Warning in checkConv(attr(opt, "derivs"), opt$par, ctrl = control$checkConv, :
## unable to evaluate scaled gradient
```

```
## Warning in checkConv(attr(opt, "derivs"), opt$par, ctrl = control$checkConv, :
## Model failed to converge: degenerate Hessian with 1 negative eigenvalues
```

```
## Warning: Model failed to converge with 1 negative eigenvalue: -1.6e+00
```

```
## [OK]
## nlminbwrap :
```

```
## Warning in optwrap(optimizer, devfun, getStart(start, rho$pp), lower =
## rho$lower, : convergence code 1 from nlminbwrap: iteration limit reached without
## convergence (10)
```

```
## Warning in checkConv(attr(opt, "derivs"), opt$par, ctrl = control$checkConv, :
## Model failed to converge with max|grad| = 0.00796476 (tol = 0.002, component 1)
```

```
## [OK]
## nmkbw :
```

```
## Warning in ctrl[namc] <- control: number of items to replace is not a multiple
## of replacement length
```

```
## Warning in checkConv(attr(opt, "derivs"), opt$par, ctrl = control$checkConv, :
## Model failed to converge with max|grad| = 0.122537 (tol = 0.002, component 1)
```

```
## [OK]
## optimx.L-BFGS-B :
```

```
## Warning in checkConv(attr(opt, "derivs"), opt$par, ctrl = control$checkConv, :
## Model failed to converge with max|grad| = 0.00484177 (tol = 0.002, component 1)
```

```
## [OK]
## nloptwrap.NLOPT_LN_NELDERMEAD :
```

```
## Warning in checkConv(attr(opt, "derivs"), opt$par, ctrl = control$checkConv, :
## Model failed to converge with max|grad| = 0.440334 (tol = 0.002, component 1)
```

```
## [OK]
## nloptwrap.NLOPT_LN_BOBYQA :
```

```
## Warning: Model failed to converge with 2 negative eigenvalues: -2.4e-04 -1.3e+00
```

```
## [OK]
```

```
## original model:
## ln_RMSSD_ms ~ (1 + slope12 + slope25 + I(slope25^2) + slope57 + I(slope57^2) ... 
## data:  HRV_TTU_Long_AllNeedTimeSubIDs 
## optimizers (7): bobyqa, Nelder_Mead, nlminbwrap, nmkbw, optimx.L-BFGS-B,nloptwrap.NLOPT_LN_N...
## differences in negative log-likelihoods:
## max= 6.07 ; std dev= 2.25
```

This is the first maximal model that does not have a convergence
issue and it is non-singular. So, run ranova to test what random effects
can be dropped without compromising model fit.

```
ln.Fix.78.910.25quad.57quad.Rand.12.25lin.57lin.89<-lmer(data=HRV_TTU_Long_AllNeedTimeSubIDs, REML=TRUE, 
                                                formula=ln_RMSSD_ms~(1+slope12+slope25+I(slope25^2)+slope57+I(slope57^2)+slope78+slope89+slope910)+
                                                (1+slope12+slope25+slope57+slope89|New_ID), 
                                                control = lmerControl(optimizer = "bobyqa", optCtrl=list(maxfun=5e5))) #NO ISSUES
ranova(ln.Fix.78.910.25quad.57quad.Rand.12.25lin.57lin.89)
```

```
## ANOVA-like table for random-effects: Single term deletions
## 
## Model:
## ln_RMSSD_ms ~ slope12 + slope25 + I(slope25^2) + slope57 + I(slope57^2) + slope78 + slope89 + slope910 + (1 + slope12 + slope25 + slope57 + slope89 | New_ID)
##                                                                 npar logLik
## <none>                                                            25 92.035
## slope12 in (1 + slope12 + slope25 + slope57 + slope89 | New_ID)   20 60.135
## slope25 in (1 + slope12 + slope25 + slope57 + slope89 | New_ID)   20 87.428
## slope57 in (1 + slope12 + slope25 + slope57 + slope89 | New_ID)   20 76.740
## slope89 in (1 + slope12 + slope25 + slope57 + slope89 | New_ID)   20 83.224
##                                                                     AIC    LRT
## <none>                                                          -134.07       
## slope12 in (1 + slope12 + slope25 + slope57 + slope89 | New_ID)  -80.27 63.800
## slope25 in (1 + slope12 + slope25 + slope57 + slope89 | New_ID) -134.86  9.214
## slope57 in (1 + slope12 + slope25 + slope57 + slope89 | New_ID) -113.48 30.589
## slope89 in (1 + slope12 + slope25 + slope57 + slope89 | New_ID) -126.45 17.622
##                                                                 Df Pr(>Chisq)
## <none>                                                                       
## slope12 in (1 + slope12 + slope25 + slope57 + slope89 | New_ID)  5  1.987e-12
## slope25 in (1 + slope12 + slope25 + slope57 + slope89 | New_ID)  5   0.100829
## slope57 in (1 + slope12 + slope25 + slope57 + slope89 | New_ID)  5  1.129e-05
## slope89 in (1 + slope12 + slope25 + slope57 + slope89 | New_ID)  5   0.003459
##                                                                    
## <none>                                                             
## slope12 in (1 + slope12 + slope25 + slope57 + slope89 | New_ID) ***
## slope25 in (1 + slope12 + slope25 + slope57 + slope89 | New_ID)    
## slope57 in (1 + slope12 + slope25 + slope57 + slope89 | New_ID) ***
## slope89 in (1 + slope12 + slope25 + slope57 + slope89 | New_ID) ** 
## ---
## Signif. codes:  0 '***' 0.001 '**' 0.01 '*' 0.05 '.' 0.1 ' ' 1
```

Dropping random slope25 does not lead to a significant worse model
fit, so drop and refit the unconditional growth curve model accordingly.
Dropping any of the other random slopes does lead to a significant worse
fit, so retain all of those. Then, run ranova again to make sure random
effects structure is not over specified.

```
ln.Fix.78.910.25quad.57quad.Rand.12.57lin.89<-lmer(data=HRV_TTU_Long_AllNeedTimeSubIDs, REML=TRUE, 
                                                formula=ln_RMSSD_ms~(1+slope12+slope25+I(slope25^2)+slope57+I(slope57^2)+slope78+slope89+slope910)+
                                                (1+slope12+slope57+slope89|New_ID), 
                                                control = lmerControl(optimizer = "bobyqa", optCtrl=list(maxfun=5e5))) #NO ISSUES
ranova(ln.Fix.78.910.25quad.57quad.Rand.12.57lin.89)
```

```
## ANOVA-like table for random-effects: Single term deletions
## 
## Model:
## ln_RMSSD_ms ~ slope12 + slope25 + I(slope25^2) + slope57 + I(slope57^2) + slope78 + slope89 + slope910 + (1 + slope12 + slope57 + slope89 | New_ID)
##                                                       npar logLik      AIC
## <none>                                                  20 87.428 -134.856
## slope12 in (1 + slope12 + slope57 + slope89 | New_ID)   16 58.867  -85.734
## slope57 in (1 + slope12 + slope57 + slope89 | New_ID)   16 66.094 -100.189
## slope89 in (1 + slope12 + slope57 + slope89 | New_ID)   16 79.392 -126.784
##                                                          LRT Df Pr(>Chisq)    
## <none>                                                                        
## slope12 in (1 + slope12 + slope57 + slope89 | New_ID) 57.122  4  1.166e-11 ***
## slope57 in (1 + slope12 + slope57 + slope89 | New_ID) 42.668  4  1.213e-08 ***
## slope89 in (1 + slope12 + slope57 + slope89 | New_ID) 16.072  4   0.002923 ** 
## ---
## Signif. codes:  0 '***' 0.001 '**' 0.01 '*' 0.05 '.' 0.1 ' ' 1
```

Dropping any of the other random slopes leads to a significant worse
fit, so retain all. Now see what quadratic time slopes are significant
fixed effects.

```
summary(ln.Fix.78.910.25quad.57quad.Rand.12.57lin.89)
```

```
## Linear mixed model fit by REML. t-tests use Satterthwaite's method [
## lmerModLmerTest]
## Formula: ln_RMSSD_ms ~ (1 + slope12 + slope25 + I(slope25^2) + slope57 +  
##     I(slope57^2) + slope78 + slope89 + slope910) + (1 + slope12 +  
##     slope57 + slope89 | New_ID)
##    Data: HRV_TTU_Long_AllNeedTimeSubIDs
## Control: lmerControl(optimizer = "bobyqa", optCtrl = list(maxfun = 5e+05))
## 
## REML criterion at convergence: -174.9
## 
## Scaled residuals: 
##     Min      1Q  Median      3Q     Max 
## -3.4151 -0.5866  0.0714  0.4670  2.5609 
## 
## Random effects:
##  Groups   Name        Variance Std.Dev. Corr             
##  New_ID   (Intercept) 0.194743 0.44130                   
##           slope12     0.071136 0.26671  -0.39            
##           slope57     0.007684 0.08766  -0.11 -0.49      
##           slope89     0.017679 0.13296   0.26  0.04 -0.62
##  Residual             0.012206 0.11048                   
## Number of obs: 310, groups:  New_ID, 31
## 
## Fixed effects:
##                Estimate Std. Error         df t value Pr(>|t|)    
## (Intercept)    3.300604   0.081705  29.999985  40.396   <2e-16 ***
## slope12        0.064184   0.055340  36.300898   1.160    0.254    
## slope25       -0.006948   0.031058 181.000007  -0.224    0.823    
## I(slope25^2)   0.004561   0.009921 181.000010   0.460    0.646    
## slope57        0.006676   0.052562 209.817954   0.127    0.899    
## I(slope57^2)   0.021766   0.024201 181.000006   0.899    0.370    
## slope78        0.006340   0.028062 181.000006   0.226    0.821    
## slope89        0.046729   0.036847  60.226283   1.268    0.210    
## slope910       0.030553   0.028062 181.000006   1.089    0.278    
## ---
## Signif. codes:  0 '***' 0.001 '**' 0.01 '*' 0.05 '.' 0.1 ' ' 1
## 
## Correlation of Fixed Effects:
##             (Intr) slop12 slop25 I(25^2 slop57 I(57^2 slop78 slop89
## slope12     -0.413                                                 
## slope25      0.000 -0.241                                          
## I(slop25^2)  0.000  0.179 -0.958                                   
## slope57     -0.031 -0.137  0.163 -0.283                            
## I(slop57^2)  0.000  0.007 -0.118  0.205 -0.917                     
## slope78      0.000  0.000  0.000  0.000  0.133 -0.290              
## slope89      0.161  0.022  0.000  0.000 -0.120  0.000 -0.381       
## slope910     0.000  0.000  0.000  0.000  0.000  0.000  0.000 -0.381
```

Neither quadratic time slope (slope25^2 and slope57^2) is
significant, drop each quadratic slope one by one, refit and
compare.

```
ln.Fix.78.910.25.Rand.12.57quad.89<-lmer(data=HRV_TTU_Long_AllNeedTimeSubIDs, REML=TRUE, 
                                                formula=ln_RMSSD_ms~(1+slope12+slope25+slope57+I(slope57^2)+slope78+slope89+slope910)+
                                                (1+slope12+slope57+I(slope57^2)+slope89|New_ID), 
                                                control = lmerControl(optimizer = "bobyqa",  optCtrl=list(maxfun=5e5))) #NO CONVERGENCE/SINGULARITY ISSUES
ranova(ln.Fix.78.910.25.Rand.12.57quad.89)
```

```
## Warning: Model failed to converge with 1 negative eigenvalue: -2.3e+00
```

```
## ANOVA-like table for random-effects: Single term deletions
## 
## Model:
## ln_RMSSD_ms ~ slope12 + slope25 + slope57 + I(slope57^2) + slope78 + slope89 + slope910 + (1 + slope12 + slope57 + I(slope57^2) + slope89 | New_ID)
##                                                                           npar
## <none>                                                                      24
## slope12 in (1 + slope12 + slope57 + I(slope57^2) + slope89 | New_ID)        19
## slope57 in (1 + slope12 + slope57 + I(slope57^2) + slope89 | New_ID)        19
## I(slope57^2) in (1 + slope12 + slope57 + I(slope57^2) + slope89 | New_ID)   19
## slope89 in (1 + slope12 + slope57 + I(slope57^2) + slope89 | New_ID)        19
##                                                                            logLik
## <none>                                                                    105.023
## slope12 in (1 + slope12 + slope57 + I(slope57^2) + slope89 | New_ID)       65.151
## slope57 in (1 + slope12 + slope57 + I(slope57^2) + slope89 | New_ID)       83.193
## I(slope57^2) in (1 + slope12 + slope57 + I(slope57^2) + slope89 | New_ID)  91.017
## slope89 in (1 + slope12 + slope57 + I(slope57^2) + slope89 | New_ID)       94.341
##                                                                                AIC
## <none>                                                                    -162.046
## slope12 in (1 + slope12 + slope57 + I(slope57^2) + slope89 | New_ID)       -92.302
## slope57 in (1 + slope12 + slope57 + I(slope57^2) + slope89 | New_ID)      -128.387
## I(slope57^2) in (1 + slope12 + slope57 + I(slope57^2) + slope89 | New_ID) -144.035
## slope89 in (1 + slope12 + slope57 + I(slope57^2) + slope89 | New_ID)      -150.681
##                                                                              LRT
## <none>                                                                          
## slope12 in (1 + slope12 + slope57 + I(slope57^2) + slope89 | New_ID)      79.745
## slope57 in (1 + slope12 + slope57 + I(slope57^2) + slope89 | New_ID)      43.660
## I(slope57^2) in (1 + slope12 + slope57 + I(slope57^2) + slope89 | New_ID) 28.012
## slope89 in (1 + slope12 + slope57 + I(slope57^2) + slope89 | New_ID)      21.365
##                                                                           Df
## <none>                                                                      
## slope12 in (1 + slope12 + slope57 + I(slope57^2) + slope89 | New_ID)       5
## slope57 in (1 + slope12 + slope57 + I(slope57^2) + slope89 | New_ID)       5
## I(slope57^2) in (1 + slope12 + slope57 + I(slope57^2) + slope89 | New_ID)  5
## slope89 in (1 + slope12 + slope57 + I(slope57^2) + slope89 | New_ID)       5
##                                                                           Pr(>Chisq)
## <none>                                                                              
## slope12 in (1 + slope12 + slope57 + I(slope57^2) + slope89 | New_ID)       9.490e-16
## slope57 in (1 + slope12 + slope57 + I(slope57^2) + slope89 | New_ID)       2.715e-08
## I(slope57^2) in (1 + slope12 + slope57 + I(slope57^2) + slope89 | New_ID)  3.621e-05
## slope89 in (1 + slope12 + slope57 + I(slope57^2) + slope89 | New_ID)       0.0006909
##                                                                              
## <none>                                                                       
## slope12 in (1 + slope12 + slope57 + I(slope57^2) + slope89 | New_ID)      ***
## slope57 in (1 + slope12 + slope57 + I(slope57^2) + slope89 | New_ID)      ***
## I(slope57^2) in (1 + slope12 + slope57 + I(slope57^2) + slope89 | New_ID) ***
## slope89 in (1 + slope12 + slope57 + I(slope57^2) + slope89 | New_ID)      ***
## ---
## Signif. codes:  0 '***' 0.001 '**' 0.01 '*' 0.05 '.' 0.1 ' ' 1
```

Dropping any of the other random slopes leads to a significant worse
fit, so retain all, even slope57^2.

```
ln.Fix.78.910.57.Rand.12.25quad.89<-lmer(data=HRV_TTU_Long_AllNeedTimeSubIDs, REML=TRUE, 
                                                formula=ln_RMSSD_ms~(1+slope12+slope25+I(slope25^2)+slope57+slope78+slope89+slope910)+
                                                (1+slope12+slope25+I(slope25^2)+slope89|New_ID), 
                                                control = lmerControl(optimizer = "bobyqa",  optCtrl=list(maxfun=5e5))) #NO CONVERGENCE/SINGULARITY ISSUES
ranova(ln.Fix.78.910.57.Rand.12.25quad.89)
```

```
## ANOVA-like table for random-effects: Single term deletions
## 
## Model:
## ln_RMSSD_ms ~ slope12 + slope25 + I(slope25^2) + slope57 + slope78 + slope89 + slope910 + (1 + slope12 + slope25 + I(slope25^2) + slope89 | New_ID)
##                                                                           npar
## <none>                                                                      24
## slope12 in (1 + slope12 + slope25 + I(slope25^2) + slope89 | New_ID)        19
## slope25 in (1 + slope12 + slope25 + I(slope25^2) + slope89 | New_ID)        19
## I(slope25^2) in (1 + slope12 + slope25 + I(slope25^2) + slope89 | New_ID)   19
## slope89 in (1 + slope12 + slope25 + I(slope25^2) + slope89 | New_ID)        19
##                                                                           logLik
## <none>                                                                    84.178
## slope12 in (1 + slope12 + slope25 + I(slope25^2) + slope89 | New_ID)      60.405
## slope25 in (1 + slope12 + slope25 + I(slope25^2) + slope89 | New_ID)      81.496
## I(slope25^2) in (1 + slope12 + slope25 + I(slope25^2) + slope89 | New_ID) 79.116
## slope89 in (1 + slope12 + slope25 + I(slope25^2) + slope89 | New_ID)      79.680
##                                                                               AIC
## <none>                                                                    -120.36
## slope12 in (1 + slope12 + slope25 + I(slope25^2) + slope89 | New_ID)       -82.81
## slope25 in (1 + slope12 + slope25 + I(slope25^2) + slope89 | New_ID)      -124.99
## I(slope25^2) in (1 + slope12 + slope25 + I(slope25^2) + slope89 | New_ID) -120.23
## slope89 in (1 + slope12 + slope25 + I(slope25^2) + slope89 | New_ID)      -121.36
##                                                                              LRT
## <none>                                                                          
## slope12 in (1 + slope12 + slope25 + I(slope25^2) + slope89 | New_ID)      47.546
## slope25 in (1 + slope12 + slope25 + I(slope25^2) + slope89 | New_ID)       5.363
## I(slope25^2) in (1 + slope12 + slope25 + I(slope25^2) + slope89 | New_ID) 10.124
## slope89 in (1 + slope12 + slope25 + I(slope25^2) + slope89 | New_ID)       8.996
##                                                                           Df
## <none>                                                                      
## slope12 in (1 + slope12 + slope25 + I(slope25^2) + slope89 | New_ID)       5
## slope25 in (1 + slope12 + slope25 + I(slope25^2) + slope89 | New_ID)       5
## I(slope25^2) in (1 + slope12 + slope25 + I(slope25^2) + slope89 | New_ID)  5
## slope89 in (1 + slope12 + slope25 + I(slope25^2) + slope89 | New_ID)       5
##                                                                           Pr(>Chisq)
## <none>                                                                              
## slope12 in (1 + slope12 + slope25 + I(slope25^2) + slope89 | New_ID)       4.397e-09
## slope25 in (1 + slope12 + slope25 + I(slope25^2) + slope89 | New_ID)          0.3732
## I(slope25^2) in (1 + slope12 + slope25 + I(slope25^2) + slope89 | New_ID)     0.0718
## slope89 in (1 + slope12 + slope25 + I(slope25^2) + slope89 | New_ID)          0.1092
##                                                                              
## <none>                                                                       
## slope12 in (1 + slope12 + slope25 + I(slope25^2) + slope89 | New_ID)      ***
## slope25 in (1 + slope12 + slope25 + I(slope25^2) + slope89 | New_ID)         
## I(slope25^2) in (1 + slope12 + slope25 + I(slope25^2) + slope89 | New_ID) .  
## slope89 in (1 + slope12 + slope25 + I(slope25^2) + slope89 | New_ID)         
## ---
## Signif. codes:  0 '***' 0.001 '**' 0.01 '*' 0.05 '.' 0.1 ' ' 1
```

Per ranova, slope25^2 is not a significant random effect…so it should
not be kept as a random effect in the model. Final model looking like
ln.Fix.78.910.25.Rand.12.57quad.89.

##

## The selected unconditional growth curve model - Third Iteration

### Summary of this unconditional growth curve model

```
summary(ln.Fix.78.910.25.Rand.12.57quad.89, ddf="Satterthwaite")
```

```
## Linear mixed model fit by REML. t-tests use Satterthwaite's method [
## lmerModLmerTest]
## Formula: ln_RMSSD_ms ~ (1 + slope12 + slope25 + slope57 + I(slope57^2) +  
##     slope78 + slope89 + slope910) + (1 + slope12 + slope57 +  
##     I(slope57^2) + slope89 | New_ID)
##    Data: HRV_TTU_Long_AllNeedTimeSubIDs
## Control: lmerControl(optimizer = "bobyqa", optCtrl = list(maxfun = 5e+05))
## 
## REML criterion at convergence: -210
## 
## Scaled residuals: 
##      Min       1Q   Median       3Q      Max 
## -2.32541 -0.52929  0.03727  0.49060  2.45852 
## 
## Random effects:
##  Groups   Name         Variance Std.Dev. Corr                   
##  New_ID   (Intercept)  0.19692  0.4438                          
##           slope12      0.07898  0.2810   -0.39                  
##           slope57      0.10142  0.3185    0.14 -0.69            
##           I(slope57^2) 0.01532  0.1238   -0.23  0.73 -0.99      
##           slope89      0.01966  0.1402    0.28 -0.09 -0.07 -0.08
##  Residual              0.01003  0.1001                          
## Number of obs: 310, groups:  New_ID, 31
## 
## Fixed effects:
##               Estimate Std. Error        df t value Pr(>|t|)    
## (Intercept)  3.301e+00  8.170e-02 3.000e+01  40.396   <2e-16 ***
## slope12      5.962e-02  5.566e-02 3.302e+01   1.071    0.292    
## slope25      6.734e-03  8.043e-03 1.520e+02   0.837    0.404    
## slope57      1.352e-02  7.180e-02 3.475e+01   0.188    0.852    
## I(slope57^2) 1.948e-02  3.090e-02 3.543e+01   0.631    0.532    
## slope78      6.340e-03  2.543e-02 1.520e+02   0.249    0.803    
## slope89      4.673e-02  3.579e-02 5.250e+01   1.306    0.197    
## slope910     3.055e-02  2.543e-02 1.520e+02   1.201    0.232    
## ---
## Signif. codes:  0 '***' 0.001 '**' 0.01 '*' 0.05 '.' 0.1 ' ' 1
## 
## Correlation of Fixed Effects:
##             (Intr) slop12 slop25 slop57 I(57^2 slop78 slop89
## slope12     -0.420                                          
## slope25      0.000 -0.217                                   
## slope57      0.111 -0.475 -0.252                            
## I(slop57^2) -0.160  0.455  0.195 -0.970                     
## slope78      0.000  0.000  0.000  0.089 -0.206              
## slope89      0.195 -0.060  0.000 -0.038 -0.042 -0.355       
## slope910     0.000  0.000  0.000  0.000  0.000  0.000 -0.355
```

### Fixed effects of this unconditional growth curve model

```
fixef(ln.Fix.78.910.25.Rand.12.57quad.89);length(fixef(ln.Fix.78.910.25.Rand.12.57quad.89))
```

```
##  (Intercept)      slope12      slope25      slope57 I(slope57^2)      slope78 
##  3.300604483  0.059623786  0.006734447  0.013517228  0.019485180  0.006340490 
##      slope89     slope910 
##  0.046729394  0.030553171
```

```
## [1] 8
```

### Get conditional mean per occasion from values of time predictors

```
CondMeans25slope<-contest1D(ln.Fix.78.910.25.Rand.12.57quad.89, ddf="Satterthwaite",                        L=c(1,0,0,0,0,0,0,0))
CondMeans25slope<-rbind(CondMeans25slope,contest1D(ln.Fix.78.910.25.Rand.12.57quad.89, ddf="Satterthwaite", L=c(1,1,0,0,0,0,0,0)))
CondMeans25slope<-rbind(CondMeans25slope,contest1D(ln.Fix.78.910.25.Rand.12.57quad.89, ddf="Satterthwaite", L=c(1,1,1,0,0,0,0,0)))
CondMeans25slope<-rbind(CondMeans25slope,contest1D(ln.Fix.78.910.25.Rand.12.57quad.89, ddf="Satterthwaite", L=c(1,1,2,0,0,0,0,0)))
CondMeans25slope<-rbind(CondMeans25slope,contest1D(ln.Fix.78.910.25.Rand.12.57quad.89, ddf="Satterthwaite", L=c(1,1,3,0,0,0,0,0)))
CondMeans25slope<-rbind(CondMeans25slope,contest1D(ln.Fix.78.910.25.Rand.12.57quad.89, ddf="Satterthwaite", L=c(1,1,3,1,1,0,0,0)))
CondMeans25slope<-rbind(CondMeans25slope,contest1D(ln.Fix.78.910.25.Rand.12.57quad.89, ddf="Satterthwaite", L=c(1,1,3,2,4,0,0,0)))
CondMeans25slope<-rbind(CondMeans25slope,contest1D(ln.Fix.78.910.25.Rand.12.57quad.89, ddf="Satterthwaite", L=c(1,1,3,2,4,1,0,0)))
CondMeans25slope<-rbind(CondMeans25slope,contest1D(ln.Fix.78.910.25.Rand.12.57quad.89, ddf="Satterthwaite", L=c(1,1,3,2,4,1,1,0)))
CondMeans25slope<-rbind(CondMeans25slope,contest1D(ln.Fix.78.910.25.Rand.12.57quad.89, ddf="Satterthwaite", L=c(1,1,3,2,4,1,1,1)))
CondMeans25slope["Timepoint"]<-c("T1","T2","T3","T4","T5","T6","T7","T8","T9","T10")
CondMeans25slope["Model"]<-c("Random slope12, slope57, slope57^2, slope89; Fix slope25, slope78, slope910 Unconditional Growth Curve Model")
CondMeans25slope_df<-subset.data.frame(CondMeans25slope, select=c("Model","Timepoint","Estimate","Std. Error","df"))
colnames(CondMeans25slope_df)<-c("Model","Timepoint","Estimate","SE","df")
CondMeans<-rbind(CondMeans,CondMeans25slope_df)
CondMeans$Model<-factor(CondMeans$Model, levels = c("Saturated Means and Unstructured Variance Model",
                                                    "Random slope12, slope27, slope89; Fixed slope27^2, slope78, slope910 Unconditional Growth Curve Model (Original model based on experimental setup/theoretical rationale)","Random slope12, slope26, slope26^2, slope89; Fix slope67, slope78, slope910 Unconditional Growth Curve Model","Random slope12, slope57, slope57^2, slope89; Fix slope25, slope78, slope910 Unconditional Growth Curve Model"))
```

##

## Model Comparison and Assessment - Third Iteration

### Need to compare the third iteration unconditional growth curve model against the first two iterations’ and the Saturated Means, Unstructured Variance Model aka the “Total Answer Key”

First, visually compare the estimated marginal means of each
model.

```
graph_4ModComps_lnRMSSD<-ggplot(CondMeans, aes(x=Timepoint, y=Estimate, group=Model)) + 
                                  geom_line(aes(colour=Model, linetype=Model, linewidth=Model)) + 
                                    scale_colour_manual(labels = ~ stringr::str_wrap(.x, width = 35),values=c("black","gray75","gray55","gray35")) + 
                                    scale_linetype_manual(labels = ~ stringr::str_wrap(.x, width = 35),values=c("dashed","dotdash","longdash","dotted")) + 
                                    scale_discrete_manual("linewidth",labels = ~ stringr::str_wrap(.x, width = 35),values=c(5,3,3,3)) + 
                                  geom_point(aes(colour=Model,shape=Model,size=Model)) + 
                                    scale_colour_manual(labels = ~ stringr::str_wrap(.x, width = 35),values=c("black","gray75","gray55","gray35")) + 
                                    scale_shape_manual(labels = ~ stringr::str_wrap(.x, width = 35),values=c(19,15,17,18)) + 
                                    scale_size_manual(labels = ~ stringr::str_wrap(.x, width = 35),values=c(10,6,6,6)) + 
                                  ggtitle("Comparing Models of the Means") +
                                  labs(y="Estimated ln(RMSSD)", x="Time period") + 
                                  theme(axis.ticks.x=element_blank(),
                                    legend.position = "right", 
                                    legend.key.width = unit(2, 'cm'),
                                    legend.key = element_blank(),
                                    legend.text=element_text(margin = margin(t = 20),size=50),
                                    #plot.title = element_text(hjust = 0.5),
                                    panel.grid.major = element_blank(),
                                    panel.grid.minor = element_blank(),
                                    panel.background = element_blank(),
                                    axis.line.x = element_line(colour = "black"),
                                    axis.line.y = element_line(colour = "black"),
                                    text=element_text(color="black", size=50),
                                    axis.text=element_text(color="black", size=40),
                                    strip.background = element_rect(color = "black",fill = "white"))
graph_4ModComps_lnRMSSD
```

Summary: Just as the Multivariate Wald test indicated, the unconditional
growth curve models selected in the 2nd and 3rd iteration are comparable
to the Saturated Means, Unstructured Variance Model.

Now test the Absolute Fit of the Model for the Means of the
unconditional growth curve model selected in the third Iteration.

Note: This is done by saturating the model and then comparing it
against the ‘Total Answer Key.’ To saturate, add the time point
predictors (as dummy codes) that are not explicitly enumerated in any of
the piecewise slopes (i.e., they are not an intercept in any of the
piecewise slopes). Once this model is built, compare it with the ‘Total
Answer Key.’ We have to do this because it is a piecewise model; it is
not the necessary process for the other types of models.

Show results using Satterthwaite DDF including -2LL as deviance.

```
Saturated.ln.Fix.78.910.25.Rand.12.57quad.89<-lmer(data=HRV_TTU_Long_AllNeedTimeSubIDs, REML=TRUE, 
                                                     formula=ln_RMSSD_ms~(1+slope12+slope25+T3+T4+slope57+I(slope57^2)+slope78+slope89+slope910)+
                                                             (1+slope12+slope57+I(slope57^2)+slope89|New_ID), 
                                                              control = lmerControl(optimizer ="optimx",  optCtrl=list(method="L-BFGS-B"))) #using this optimizer to avoid a convergence issue
```

```
## Warning in optwrap(optimizer, devfun, getStart(start, rho$pp), lower =
## rho$lower, : convergence code 1 from optimx: none
```

```
## Warning in checkConv(attr(opt, "derivs"), opt$par, ctrl = control$checkConv, :
## Model failed to converge with max|grad| = 0.00355624 (tol = 0.002, component 1)
```

```
summary(Saturated.ln.Fix.78.910.25.Rand.12.57quad.89, ddf="Satterthwaite"); llikAIC(Saturated.ln.Fix.78.910.25.Rand.12.57quad.89, chkREML=FALSE)
```

```
## Linear mixed model fit by REML. t-tests use Satterthwaite's method [
## lmerModLmerTest]
## Formula: 
## ln_RMSSD_ms ~ (1 + slope12 + slope25 + T3 + T4 + slope57 + I(slope57^2) +  
##     slope78 + slope89 + slope910) + (1 + slope12 + slope57 +  
##     I(slope57^2) + slope89 | New_ID)
##    Data: HRV_TTU_Long_AllNeedTimeSubIDs
## Control: lmerControl(optimizer = "optimx", optCtrl = list(method = "L-BFGS-B"))
## 
## REML criterion at convergence: -198.9
## 
## Scaled residuals: 
##      Min       1Q   Median       3Q      Max 
## -2.22564 -0.52248  0.04549  0.47001  2.45558 
## 
## Random effects:
##  Groups   Name         Variance Std.Dev. Corr                   
##  New_ID   (Intercept)  0.19683  0.4437                          
##           slope12      0.07879  0.2807   -0.39                  
##           slope57      0.10095  0.3177    0.14 -0.69            
##           I(slope57^2) 0.01520  0.1233   -0.23  0.73 -0.99      
##           slope89      0.01955  0.1398    0.29 -0.09 -0.07 -0.08
##  Residual              0.01013  0.1007                          
## Number of obs: 310, groups:  New_ID, 31
## 
## Fixed effects:
##                Estimate Std. Error         df t value Pr(>|t|)    
## (Intercept)    3.300604   0.081707  29.997416  40.395   <2e-16 ***
## slope12        0.062632   0.056526  35.176122   1.108    0.275    
## slope25        0.007769   0.008522 150.502293   0.912    0.363    
## T3            -0.003948   0.022548 150.502281  -0.175    0.861    
## T4            -0.014295   0.022548 150.502286  -0.634    0.527    
## slope57        0.004348   0.073354  37.804342   0.059    0.953    
## I(slope57^2)   0.022542   0.031313  37.372730   0.720    0.476    
## slope78        0.006340   0.025567 150.502281   0.248    0.804    
## slope89        0.046729   0.035836  52.772723   1.304    0.198    
## slope910       0.030553   0.025567 150.502280   1.195    0.234    
## ---
## Signif. codes:  0 '***' 0.001 '**' 0.01 '*' 0.05 '.' 0.1 ' ' 1
## 
## Correlation of Fixed Effects:
##             (Intr) slop12 slop25 T3     T4     slop57 I(57^2 slop78 slop89
## slope12     -0.413                                                        
## slope25      0.000 -0.226                                                 
## T3           0.000 -0.171  0.189                                          
## T4           0.000 -0.085 -0.189  0.286                                   
## slope57      0.108 -0.481 -0.261  0.099  0.198                            
## I(slop57^2) -0.157  0.460  0.204 -0.077 -0.154 -0.970                     
## slope78      0.000  0.000  0.000  0.000  0.000  0.087 -0.204              
## slope89      0.195 -0.059  0.000  0.000  0.000 -0.038 -0.040 -0.357       
## slope910     0.000  0.000  0.000  0.000  0.000  0.000  0.000  0.000 -0.357
## optimizer (optimx) convergence code: 1 (none)
## Model failed to converge with max|grad| = 0.00355624 (tol = 0.002, component 1)
```

```
## $logLik
## 'log Lik.' 99.43114 (df=26)
## 
## $AICtab
##        AIC        BIC     logLik   deviance   df.resid 
## -146.86229  -49.71141   99.43114 -198.86229  284.00000
```

```
fixef(Saturated.ln.Fix.78.910.25.Rand.12.57quad.89);length(fixef(Saturated.ln.Fix.78.910.25.Rand.12.57quad.89))
```

```
##  (Intercept)      slope12      slope25           T3           T4      slope57 
##  3.300604483  0.062632323  0.007769231 -0.003947505 -0.014295345  0.004347895 
## I(slope57^2)      slope78      slope89     slope910 
##  0.022541624  0.006340490  0.046729394  0.030553171
```

```
## [1] 10
```

Does Saturated.ln.Fix.78.910.25.Rand.12.57quad.89 reproduce the Model
for the Means of the Saturated Means, Unstructured Variance model?

```
contestMD(Saturated.ln.Fix.78.910.25.Rand.12.57quad.89, ddf="Satterthwaite", L=rbind(c(0,0,0,1,0,0,0,0,0,0),c(0,0,0,0,1,0,0,0,0,0)))
```

```
##        Sum Sq     Mean Sq NumDF    DenDF   F value    Pr(>F)
## 1 0.004072947 0.002036474     2 150.5023 0.2009883 0.8181412
```

F(2,150.5023)=0.2009883, p=0.8181412, suggesting there is *no*
evidence to suggest the Model for the Means of the Saturated Means,
Unstructured Variance model is significantly different than this
Unconditional Growth Curve Model’s Model for the Means. In other words,
this unconditional growth curve model is capturing the mean value over
time just as well as the saturated means, unstructured variance
model.

Now compare the model for the variance of this unconditional growth
curve model and the one produced by the saturated means, unstructured
variance model. We compare with a likelihood ratio test (LRT).
Procedural note: Have to refit both of these models with lme in order to
specify the random effects correctly.

```
lnSameFix4UnVarREML = gls(data=HRV_TTU_Long_AllNeedTimeSubIDs, method="REML", 
                           model=ln_RMSSD_ms~1+slope12+slope25+slope57+I(slope57^2)+slope78+slope89+slope910, 
                           correlation=corSymm(form=~as.numeric(Timepoint)|New_ID), # Unstructured correlations
                           weights=varIdent(form=~1|(Timepoint)))                   # Heterogeneous variances

#this is the same as ln.Fix.78.910.24.Rand.12.47.89, now just fit with LME
lmeREML.ln.Fix.78.910.25.Rand.12.57quad.89<-lme(data=HRV_TTU_Long_AllNeedTimeSubIDs, method="REML", 
                                              ln_RMSSD_ms~1+slope12+slope25+slope57+I(slope57^2)+slope78+slope89+slope910,
                                                random=~1+slope12+slope57+I(slope57^2)+slope89|New_ID,
                                                control=nlmeControl(msMaxIter=400))
anova(lnSameFix4UnVarREML, lmeREML.ln.Fix.78.910.25.Rand.12.57quad.89)
```

```
##                                            Model df       AIC       BIC
## lnSameFix4UnVarREML                            1 63 -130.4614 103.29548
## lmeREML.ln.Fix.78.910.25.Rand.12.57quad.89     2 24 -162.0464 -72.99619
##                                              logLik   Test  L.Ratio p-value
## lnSameFix4UnVarREML                        128.2307                        
## lmeREML.ln.Fix.78.910.25.Rand.12.57quad.89 105.0232 1 vs 2 46.41498  0.1932
```

-2LL=46.41498, p=0.1932, suggesting there is *no* evidence
that the Model for the Variance of this unconditional growth curve model
is significantly different than that of the Saturated Means,
Unstructured Variance model. Therefore, this could serve as the final
unconditional growth curve model of cardiac vagal tone.

```
TotalVar4Mods<-data.frame(Model=character(), Timepoint=character(), TotalVar=numeric())
TotalVar4Mods[1:10,"Model"]<-"Saturated Means and Unstructured Variance Model"
TotalVar4Mods[11:20,"Model"]<-"Random slope12, slope27, slope89; Fixed slope27^2, slope78, slope910 Unconditional Growth Curve Model (Original model based on experimental setup/theoretical rationale)"
TotalVar4Mods[21:30,"Model"]<-"Random slope12, slope26, slope26^2, slope89; Fix slope67, slope78, slope910 Unconditional Growth Curve Model"
TotalVar4Mods[31:40,"Model"]<-c("Random slope12, slope57, slope57^2, slope89; Fix slope25, slope78, slope910 Unconditional Growth Curve Model")
TotalVar4Mods$Model<-factor(TotalVar4Mods$Model, levels = c("Saturated Means and Unstructured Variance Model",
"Random slope12, slope27, slope89; Fixed slope27^2, slope78, slope910 Unconditional Growth Curve Model (Original model based on experimental setup/theoretical rationale)", "Random slope12, slope26, slope26^2, slope89; Fix slope67, slope78, slope910 Unconditional Growth Curve Model","Random slope12, slope57, slope57^2, slope89; Fix slope25, slope78, slope910 Unconditional Growth Curve Model"))

TotalVar4Mods[1:10,"Timepoint"]<-c("T1","T2","T3","T4","T5","T6","T7","T8","T9","T10")
TotalVar4Mods[11:20,"Timepoint"]<-c("T1","T2","T3","T4","T5","T6","T7","T8","T9","T10")
TotalVar4Mods[21:30,"Timepoint"]<-c("T1","T2","T3","T4","T5","T6","T7","T8","T9","T10")
TotalVar4Mods[31:40,"Timepoint"]<-c("T1","T2","T3","T4","T5","T6","T7","T8","T9","T10")
TotalVar4Mods$Timepoint<-factor(TotalVar4Mods$Timepoint, levels = c("T1","T2","T3","T4","T5","T6","T7","T8","T9","T10"))  

TotalVar4Mods[1:10,"TotalVar"]<-(diag(getVarCov(lnSatUN, New_ID="6", type="marginal")))
TotalVar4Mods[11:20,"TotalVar"]<-(diag(getVarCov(lmeREML.ln.Fix910.27quad.78.Rand.12.27lin.89, New_ID="6", type="marginal")[[1]]))
TotalVar4Mods[21:30,"TotalVar"]<-(diag(getVarCov(lmeREML.ln.Fix.67.78.910.Rand.12.26quad.89, New_ID="6", type="marginal")[[1]]))
TotalVar4Mods[31:40,"TotalVar"]<-(diag(getVarCov(lmeREML.ln.Fix.78.910.25.Rand.12.57quad.89, New_ID="6", type="marginal")[[1]]))

row.names(TotalVar4Mods)<-1:nrow(TotalVar4Mods)
```

### Visually compare the Model for the Variance of all models

```
graph_CompTotVar4Mods_lnRMSSD<-ggplot(TotalVar4Mods, aes(x=Timepoint, y=TotalVar, group=Model)) + 
                                      geom_line(aes(colour=Model, linetype=Model, linewidth=Model)) + 
                                        scale_colour_manual(labels = ~ stringr::str_wrap(.x, width = 35),values=c("black","gray75","gray55","gray35")) + 
                                        scale_linetype_manual(labels = ~ stringr::str_wrap(.x, width = 35),values=c("dashed","dotdash","longdash","dotted")) + 
                                        scale_discrete_manual("linewidth",labels = ~ stringr::str_wrap(.x, width = 35),values=c(5,3,3,3)) + 
                                    geom_point(aes(colour=Model,shape=Model,size=Model)) + 
                                        scale_colour_manual(labels = ~ stringr::str_wrap(.x, width = 35),values=c("black","gray75","gray55","gray35")) + 
                                        scale_shape_manual(labels = ~ stringr::str_wrap(.x, width = 35),values=c(19,15,17,18)) + 
                                        scale_size_manual(labels = ~ stringr::str_wrap(.x, width = 35),values=c(10,6,6,6)) + 
                                    ggtitle("Comparing models of the variance of ln(RMSSD) across all time periods (T1-T10) (includes both low and high taskload)") +
                                      labs(y="Estimated ln(RMSSD)", x="Time period") + 
                                      theme(axis.ticks.x=element_blank(),
                                        legend.position = "right", 
                                        legend.key.width = unit(2, 'cm'),
                                        legend.key = element_blank(),
                                        legend.text=element_text(margin = margin(t = 20),size=20),
                                       # plot.title = element_text(hjust = 0.5),
                                        panel.grid.major = element_blank(),
                                        panel.grid.minor = element_blank(),
                                        panel.background = element_blank(),
                                        axis.line.x = element_line(colour = "black"),
                                        axis.line.y = element_line(colour = "black"),
                                        text=element_text(color="black", size=30),
                                        axis.text=element_text(color="black", size=30),
                                        strip.background = element_rect(color = "black",fill = "white"))
  graph_CompTotVar4Mods_lnRMSSD
```

Summary: Per visual inspection, it seems like the Model for the
Variance of the model selected during the third iteration had the most
deviation from the Model for the Variance of the Saturated Means,
Unstructured Variance model. However, starting at time period 6, it
aligns quite nicely.

## Review all models built and select a final unconditional growth curve model - Rely on BIC

Given ln.Fix.78.910.25.Rand.12.57quad.89 and
ln.Fix.67.78.910.Rand.12.26quad.89 do not significantly differ from the
Saturated Means, Unstructured Variance model’s Model for the Means and
Model for the Variance, compare these two models’ BIC values to decide
on a *“best fitting”* unconditional growth curve model to use for
interpretation and conditional growth curve modeling.

Note: Have to fit model with ML to get an accurate and comparable BIC
value.

```
lmeML.ln.Fix.67.78.910.Rand.12.26quad.89<-lme(data=HRV_TTU_Long_AllNeedTimeSubIDs, method="ML", 
                                            ln_RMSSD_ms~1+slope12+slope26+I(slope26^2)+slope67+slope78+slope89+slope910,
                                              random=~1+slope12+slope26+I(slope26^2)+slope89|New_ID,
                                              control=lmeControl(opt = "optim", optimMethod = "BFGS", msMaxIter=2000, msMaxEval=2000))

lmeML.ln.Fix.78.910.25.Rand.12.57quad.89<-lme(data=HRV_TTU_Long_AllNeedTimeSubIDs, method="ML", 
                                            ln_RMSSD_ms~1+slope12+slope25+slope57+I(slope57^2)+slope78+slope89+slope910,
                                              random=~1+slope12+slope57+I(slope57^2)+slope89|New_ID,
                                              control=lmeControl(opt = "optim", optimMethod = "BFGS", msMaxIter=2000, msMaxEval=2000))

BIC(lmeML.ln.Fix.67.78.910.Rand.12.26quad.89)
```

```
## [1] -112.2214
```

```
BIC(lmeML.ln.Fix.78.910.25.Rand.12.57quad.89)
```

```
## [1] -116.7254
```

The BIC for the model that has slope78 and slope910 as a fixed effect
and all else as a random effect is smaller than the BIC for the model
that has slope67, slope78, and slope910 as a fixed effect and all else
as random effects (i.e., BIC\_ln.Fix.78.910.25.Rand.12.57quad.89=116.7254
< BIC\_ln.Fix.67.78.910.Rand.12.26quad.89=-112.2214). Therefore, the
model ln.Fix.78.910.25.Rand.12.57quad.89 is the “best fitting”
unconditional growth curve model for this data as it (1) is better than
all competing models, (2) meets the 3 modeling assumptions of linearity,
homoscedacity, and normality of residuals (shown below), and (3) does
not significantly differ from the Saturated Means, Unstructured Variance
Model.

## Evaluate model assumptions of the final unconditional growth curve model

Evaluate whether the final model, ln.Fix.78.910.25.Rand.12.57quad.89,
violates any assumptions. {.tabset} Note: The assumptions are discussed
here
and the below code is from here:

### 1. Assumption #1: Linearity. Assessed by plotting Residuals vs Fitted and seeing if there is any pattern aka any indication the linear predictors are not accounting for the data

```
plot(ln.Fix.78.910.25.Rand.12.57quad.89, type=c("p","smooth"), col.line=1, main="Fitted vs Residuals Plot for ln.Fix.78.910.25.Rand.12.57quad.89")
```

### 2. Assumption #2 (part 1 of 2): Homogeneity of Variance. Assess this with a Scale-Location plot, which show if residuals are spread equally along the ranges of predictors. Homogeneity of Variance is present if you see a horizontal line with equally (randomly) spread points

```
plot(ln.Fix.78.910.25.Rand.12.57quad.89,
     sqrt(abs(resid(.)))~fitted(.),
     type=c("p","smooth"), col.line=1, main="Scale-Location Plot for ln.Fix.78.910.25.Rand.12.57quad.89")
```

### 2. Assumption #2 (part 2 of 2): Levene’s test of homogeneity (note:
not often assessed in growth curve modeling)

```
HRV_TTU_Long_AllNeedTimeSubIDs$ln.Fix.78.910.25.Rand.12.57quad.89.Res<-residuals(ln.Fix.78.910.25.Rand.12.57quad.89) #extracts the residuals and places them in a new column in original data table
HRV_TTU_Long_AllNeedTimeSubIDs$Abs.ln.Fix.78.910.25.Rand.12.57quad.89.Res<-abs(HRV_TTU_Long_AllNeedTimeSubIDs$ln.Fix.78.910.25.Rand.12.57quad.89.Res) #creates a new column with the absolute value of the residuals
HRV_TTU_Long_AllNeedTimeSubIDs$ln.Fix.78.910.25.Rand.12.57quad.89.Res.sqrd<-HRV_TTU_Long_AllNeedTimeSubIDs$Abs.ln.Fix.78.910.25.Rand.12.57quad.89.Res^2 #squares the absolute values of the residuals to provide the more robust estimate
Levene.Abs.ln.Fix.78.910.25.Rand.12.57quad.89<-lm(ln.Fix.78.910.25.Rand.12.57quad.89.Res.sqrd ~ New_ID, data=HRV_TTU_Long_AllNeedTimeSubIDs) #ANOVA of the squared residuals
anova(Levene.Abs.ln.Fix.78.910.25.Rand.12.57quad.89)
```

```
## Analysis of Variance Table
## 
## Response: ln.Fix.78.910.25.Rand.12.57quad.89.Res.sqrd
##            Df   Sum Sq    Mean Sq F value  Pr(>F)  
## New_ID     30 0.003571 1.1903e-04  1.4004 0.08598 .
## Residuals 279 0.023715 8.5001e-05                  
## ---
## Signif. codes:  0 '***' 0.001 '**' 0.01 '*' 0.05 '.' 0.1 ' ' 1
```

p=0.089, suggesting the assumption of Homogeneity of Variance is
holding.

### 3. Assumption #3: The residuals of the model are normally distributed

```
lattice::qqmath(ln.Fix.78.910.25.Rand.12.57quad.89, main="QQplot for ln.Fix.78.910.25.Rand.12.57quad.89") #not perfect, but not massive deviations from the qqline, which plots out what perfectly normally distributed residuals would look like
```

### 95% Confidence Intervals (CIs) for Random Effects

These CIs describe the estimated range of individual differences in
the random time slopes (in units of ln(RMSSD)).

```
RandEffVars_slope25<-as.data.frame(VarCorr(ln.Fix.78.910.25.Rand.12.57quad.89)$New_ID)
CI95_df_slope25<-data.frame(RandEff=as.character(),LowerLim=as.numeric(),UpperLim=as.numeric())
CI95_df_slope25[1,"RandEff"]="Intercept"
CI95_df_slope25[1,"LowerLim"]=fixef(ln.Fix.78.910.25.Rand.12.57quad.89)[1]-(1.96*(sqrt(as.numeric(RandEffVars_slope25["(Intercept)","(Intercept)"]))))
CI95_df_slope25[1,"UpperLim"]=fixef(ln.Fix.78.910.25.Rand.12.57quad.89)[1]+(1.96*(sqrt(as.numeric(RandEffVars_slope25["(Intercept)","(Intercept)"]))))
CI95_df_slope25[2,"RandEff"]="slope12"
CI95_df_slope25[2,"LowerLim"]=fixef(ln.Fix.78.910.25.Rand.12.57quad.89)[2]-(1.96*(sqrt(as.numeric(RandEffVars_slope25["slope12","slope12"]))))
CI95_df_slope25[2,"UpperLim"]=fixef(ln.Fix.78.910.25.Rand.12.57quad.89)[2]+(1.96*(sqrt(as.numeric(RandEffVars_slope25["slope12","slope12"]))))
CI95_df_slope25[3,"RandEff"]="slope57"
CI95_df_slope25[3,"LowerLim"]=fixef(ln.Fix.78.910.25.Rand.12.57quad.89)[4]-(1.96*(sqrt(as.numeric(RandEffVars_slope25["slope57","slope57"]))))
CI95_df_slope25[3,"UpperLim"]=fixef(ln.Fix.78.910.25.Rand.12.57quad.89)[4]+(1.96*(sqrt(as.numeric(RandEffVars_slope25["slope57","slope57"]))))
CI95_df_slope25[4,"RandEff"]="I(slope57^2)"
CI95_df_slope25[4,"LowerLim"]=fixef(ln.Fix.78.910.25.Rand.12.57quad.89)[5]-(1.96*(sqrt(as.numeric(RandEffVars_slope25["I(slope57^2)","I(slope57^2)"]))))
CI95_df_slope25[4,"UpperLim"]=fixef(ln.Fix.78.910.25.Rand.12.57quad.89)[5]+(1.96*(sqrt(as.numeric(RandEffVars_slope25["I(slope57^2)","I(slope57^2)"]))))
CI95_df_slope25[5,"RandEff"]="slope89"
CI95_df_slope25[5,"LowerLim"]=fixef(ln.Fix.78.910.25.Rand.12.57quad.89)[7]-(1.96*(sqrt(as.numeric(RandEffVars_slope25["slope89","slope89"]))))
CI95_df_slope25[5,"UpperLim"]=fixef(ln.Fix.78.910.25.Rand.12.57quad.89)[7]+(1.96*(sqrt(as.numeric(RandEffVars_slope25["slope89","slope89"]))));CI95_df_slope25
```

```
##        RandEff   LowerLim  UpperLim
## 1    Intercept  2.4308371 4.1703719
## 2      slope12 -0.4911942 0.6104418
## 3      slope57 -0.6106857 0.6377201
## 4 I(slope57^2) -0.2230819 0.2620522
## 5      slope89 -0.2281167 0.3215755
```

## Unconditional Growth Curve Model Plots

Plot different variations of the “best fitting” unconditional growth
curve model (i.e., ln.Fix.78.910.25.Rand.12.57quad.89) in order to
illustrate what its estimating for cardiac vagal tone across all the
participants.

Plot estimated trajectories of all participants.

```
df_HRV_TTU_Long_AllSubIDs_EstimatedGCM<-subset.data.frame(HRV_TTU_Long_AllNeedTimeSubIDs, select = c(New_ID,Timepoint,Time,slope12,slope25,slope57,slope78,slope89,slope910))
df_HRV_TTU_Long_AllSubIDs_EstimatedGCM["Pred_ln_RMSSD"]<-predict(ln.Fix.78.910.25.Rand.12.57quad.89,df_HRV_TTU_Long_AllSubIDs_EstimatedGCM)

graph_EstGCMs<-ggplot(df_HRV_TTU_Long_AllSubIDs_EstimatedGCM, aes(x=Timepoint, y=Pred_ln_RMSSD, group=New_ID)) + 
                  geom_line(aes(color=New_ID), linetype="dashed", linewidth=5) + geom_point(aes(color=New_ID), shape=19, size=7) +  
                  ggtitle(paste0("Estimated unconditional growth curve model of ln(RMSSD) for ",length(unique(df_HRV_TTU_Long_AllSubIDs_EstimatedGCM$New_ID))," participants (not controlling for taskload)")) +  
                  labs(y="Estimated ln(RMSSD)", x="Time period",color="Participant") + theme(axis.ticks.x=element_blank(),
                      legend.position = "right", 
                      plot.title = element_text(hjust = 0.5),
                      panel.grid.major = element_blank(),
                      panel.grid.minor = element_blank(),
                      panel.background = element_blank(),
                      axis.line.x = element_line(colour = "black"),
                      axis.line.y = element_line(colour = "black"),
                      text=element_text(color="black", size=50),
                      axis.text=element_text(color="black", size=40),
                      strip.background = element_rect(color = "black",fill = "white")) 
graph_EstGCMs
```

Plot estimated trajectories for 3 participants’ whose estimated
greatly differ from one another.

```
df_HRV_TTU_Long_AllSubIDs_EstimatedGCM_SpecNewIDs<-subset.data.frame(HRV_TTU_Long_AllNeedTimeSubIDs, as.numeric(HRV_TTU_Long_AllNeedTimeSubIDs$New_ID)==31 | as.numeric(HRV_TTU_Long_AllNeedTimeSubIDs$New_ID)==6| as.numeric(HRV_TTU_Long_AllNeedTimeSubIDs$New_ID)==21, select = c(New_ID,Timepoint,Time,slope12,slope25,slope57,slope78,slope89,slope910))
  df_HRV_TTU_Long_AllSubIDs_EstimatedGCM_SpecNewIDs["Pred_ln_RMSSD"]<-predict(ln.Fix.78.910.25.Rand.12.57quad.89,df_HRV_TTU_Long_AllSubIDs_EstimatedGCM_SpecNewIDs)
  color_NewID_3<-c("#CD9609","#00A9FF","#F8768A")
  graph_preds_EstGCMs_SpecNewIDs<-ggplot(df_HRV_TTU_Long_AllSubIDs_EstimatedGCM_SpecNewIDs, aes(x=Timepoint, y=Pred_ln_RMSSD, group=New_ID)) + 
                    geom_line(aes(color=New_ID), linetype="dashed", linewidth=3) + geom_point(aes(color=New_ID), shape=19, size=5) +  
                    ggtitle(paste0("Estimated unconditional growth curve model of ln(RMSSD) for",length(unique(df_HRV_TTU_Long_AllSubIDs_EstimatedGCM_SpecNewIDs$New_ID))," participants (not controlling for taskload)")) +
                    labs(y="Estimated ln(RMSSD)", x="Time period", color="Participant") + 
                    theme(axis.ticks.x=element_blank(),
                        legend.position = "right", 
                        #plot.title = element_text(hjust = 0.5),
                        panel.grid.major = element_blank(),
                        panel.grid.minor = element_blank(),
                        panel.background = element_blank(),
                        axis.line.x = element_line(colour = "black"),
                        axis.line.y = element_line(colour = "black"),
                        text=element_text(color="black", size=50),
                        axis.text=element_text(color="black", size=40),
                        strip.background = element_rect(color = "black",fill = "white")) 
graph_preds_EstGCMs_SpecNewIDs + scale_color_manual(values = color_NewID_3)
```

# Model Building - Conditional Models

In this phase of model building, we are adding predictors to multiple
parameters in the model. We are interested in their overall effect on
the model, not just their effect on the parameter estimate. This means
we do not look to the results of the Wald test to assess the moderation
effect, but rather we need to conduct a multivariate Wald test, which
can be done with the R function contestMD. A multivariate Wald test is
analagous to studying if change in R^2 in multiple linear regression
models is significant or not, based on the predictors in the more
complex model. It is also analogous to a likelihood ratio test comparing
nested models, and we would use this test if we were fitting models with
ML. In summary, we will see if this conditional model accounts for
significantly more variance by assessing the significance of a
multivariate Wald test.

Additional info: Lesa Hoffman in video PSQF6271\_Example7b\_Part1 (go
to minute 32 and minute 41 in this video)
says we are not interested in the results of the Wald test that are
produced in with summary(). Rather, we need to look at the custom F
tests and the change in Total and Psuedo R^2 (R-squared), as well as
significant differences of simple slopes. This is because adding a
predictor (of any kind, time-invariant or time-variant, etc.) will
always increase R^2, but we want to make sure we are adding a predictor
that *significantly* increases it, aka the model fit improves
when it is added. The Wald test does not assess this as it is not
assessing significance based on all the predictors that are added to the
model at once. Therefore, we need an omnibus test as this is precisely
what omnibus tests do. They specifically assess how much parameter
estimate variance is accounted for by the the predictor and if that
amount accounted for is significant. If it is, then we can interpret the
results of the Wald test.

Before we can build the conditional growth curve models, we need to
import the performance data and match it with the HRV data.

```
DownselPerfMeas_TTU_RawData<-subset.data.frame(AllMeas_TTU_RawData, AllMeas_TTU_RawData$ParticipantID!=46, #removing this participant as it is the one with the inexplicably/unrealistic low HRV
  select = c(ParticipantID,TaskLoadCondition,Population,BreakCondition, NotMeetPerformanceCriteria,MissingData,RandomAssignCondition,Age,Handedness,Sex,
             Pre_Block1_A_ExtremeCorrect,Pre_Block2_A_ExtremeCorrect,Pre_Block3_A_ExtremeCorrect,Post_Block5_A_ExtremeCorrect,HitRTmdB1,HitRTmdB2,HitRTmdB3,HitRTmdB5))

#Match DownselPerfMeas data and HRV data 
DownselPerfMeas_TTU_Data<-DownselPerfMeas_TTU_RawData
colnames(DownselPerfMeas_TTU_Data)[1]<-"Original_ID"
colnames(DownselPerfMeas_TTU_Data)[4]<-"BreakType"
colnames(HRV_TTU_Long_AllNeedTimeSubIDs)[3]<-"TaskLoadCondition"

DownselPerfMeas_TTU_Data$Population[which(DownselPerfMeas_TTU_Data$Population==1)]="TTU"
#figure out what TaskLoadCondition==1 vs 2 in DownselPerfMeas_TTU_Data means in terms of H vs L Task Load
#unique(paste0(DownselPerfMeas_TTU_Data$Original_ID,"_",DownselPerfMeas_TTU_Data$TaskLoadCondition))
#unique(paste0(HRV_TTU_Long_AllNeedTimeSubIDs$Original_ID,"_",HRV_TTU_Long_AllNeedTimeSubIDs$TaskLoadCondition))
DownselPerfMeas_TTU_Data$TaskLoadCondition[which(DownselPerfMeas_TTU_Data$Original_ID==14)]<-2 #need to manually change the task load for Original_ID 14 (it was inputted in to the master data file incorrectly - you'll notice we also changed it in the HRV data file)
DownselPerfMeas_TTU_Data$TaskLoadCondition[which(DownselPerfMeas_TTU_Data$TaskLoadCondition==1)]="H"
DownselPerfMeas_TTU_Data$TaskLoadCondition[which(DownselPerfMeas_TTU_Data$TaskLoadCondition==2)]="L"
DownselPerfMeas_TTU_Data$TaskLoadCondition<-as.factor(DownselPerfMeas_TTU_Data$TaskLoadCondition)
#Map BreakType 1 vs 2 in DownselPerfMeas_TTU_Data to U vs N BreakType
#unique(paste0(DownselPerfMeas_TTU_Data$Original_ID,"_",DownselPerfMeas_TTU_Data$BreakType))
#unique(paste0(HRV_TTU_Long_AllNeedTimeSubIDs$Original_ID,"_",HRV_TTU_Long_AllNeedTimeSubIDs$BreakType))
DownselPerfMeas_TTU_Data$BreakType[which(DownselPerfMeas_TTU_Data$BreakType==1)]="U"
DownselPerfMeas_TTU_Data$BreakType[which(DownselPerfMeas_TTU_Data$BreakType==2)]="N"

#merge together the HRV and (the reduced) AllMeas data and reorder/restructure/rename the data frame accordingly
HRV_Perf_TTU_Long_AllNeedTimeSubIDs<-merge.data.frame(HRV_TTU_Long_AllNeedTimeSubIDs,DownselPerfMeas_TTU_Data, by=c("Original_ID","TaskLoadCondition","Population","BreakType"))
#colnames(HRV_Perf_TTU_Long_AllNeedTimeSubIDs)
Downsel_HRV_Perf_TTU_Long_AllNeedTimeSubIDs<-subset.data.frame(HRV_Perf_TTU_Long_AllNeedTimeSubIDs, select = -c(slope27,T1,T2,T3,T4,T5,T6,T7,T8,T9,T10,slope26,slope67))
colnames(Downsel_HRV_Perf_TTU_Long_AllNeedTimeSubIDs)
```

```
##  [1] "Original_ID"                                
##  [2] "TaskLoadCondition"                          
##  [3] "Population"                                 
##  [4] "BreakType"                                  
##  [5] "New_ID"                                     
##  [6] "FileName"                                   
##  [7] "Timepoint"                                  
##  [8] "Timeperiod"                                 
##  [9] "BeatsCorrected_Percent"                     
## [10] "RMSSD_ms"                                   
## [11] "Block"                                      
## [12] "Time"                                       
## [13] "slope12"                                    
## [14] "slope78"                                    
## [15] "slope89"                                    
## [16] "slope910"                                   
## [17] "ln_RMSSD_ms"                                
## [18] "slope25"                                    
## [19] "slope57"                                    
## [20] "ln.Fix.78.910.25.Rand.12.57quad.89.Res"     
## [21] "Abs.ln.Fix.78.910.25.Rand.12.57quad.89.Res" 
## [22] "ln.Fix.78.910.25.Rand.12.57quad.89.Res.sqrd"
## [23] "NotMeetPerformanceCriteria"                 
## [24] "MissingData"                                
## [25] "RandomAssignCondition"                      
## [26] "Age"                                        
## [27] "Handedness"                                 
## [28] "Sex"                                        
## [29] "Pre_Block1_A_ExtremeCorrect"                
## [30] "Pre_Block2_A_ExtremeCorrect"                
## [31] "Pre_Block3_A_ExtremeCorrect"                
## [32] "Post_Block5_A_ExtremeCorrect"               
## [33] "HitRTmdB1"                                  
## [34] "HitRTmdB2"                                  
## [35] "HitRTmdB3"                                  
## [36] "HitRTmdB5"
```

```
#Downsel_HRV_Perf_TTU_Long_AllNeedTimeSubIDs<-Downsel_HRV_Perf_TTU_Long_AllNeedTimeSubIDs[c(5,1,3,2,4,6,7,8,12,11,9,10,18,13,14,15,16,17,19,20,21,22,23,24,25,26,27,28,29,30,31,32,33,34,35)]

colnames(Downsel_HRV_Perf_TTU_Long_AllNeedTimeSubIDs)[32]<-"Post_Block4_A_ExtremeCorrect"
colnames(Downsel_HRV_Perf_TTU_Long_AllNeedTimeSubIDs)[36]<-"HitRTmdB4"
Downsel_HRV_Perf_TTU_Long_AllNeedTimeSubIDs<-Downsel_HRV_Perf_TTU_Long_AllNeedTimeSubIDs[order(Downsel_HRV_Perf_TTU_Long_AllNeedTimeSubIDs$New_ID,Downsel_HRV_Perf_TTU_Long_AllNeedTimeSubIDs$Timepoint,Downsel_HRV_Perf_TTU_Long_AllNeedTimeSubIDs$Timeperiod),]
row.names(Downsel_HRV_Perf_TTU_Long_AllNeedTimeSubIDs)<-1:nrow(Downsel_HRV_Perf_TTU_Long_AllNeedTimeSubIDs)
```

Calculate delta A as this is the measure of performance used when
testing the effect vigilance task performance has on cardiac vagal tone
over time. Calculate its summary stats and center it for conditional
growth curve model building.

```
Downsel_HRV_Perf_TTU_Long_AllNeedTimeSubIDs["A_ExtremeCorrect_DeltaB1vsB3"]<-(Downsel_HRV_Perf_TTU_Long_AllNeedTimeSubIDs$Pre_Block1_A_ExtremeCorrect)-(Downsel_HRV_Perf_TTU_Long_AllNeedTimeSubIDs$Pre_Block3_A_ExtremeCorrect)

length(unique(Downsel_HRV_Perf_TTU_Long_AllNeedTimeSubIDs$A_ExtremeCorrect_DeltaB1vsB3)) == 31 #need this to unique for the correct sd calculation...however the length of unique(<WHATEVER>) == 31 bc we need everyone's value represented, even if it is a repeat of another participant's value
```

```
## [1] TRUE
```

```
#if the statement above is TRUE, run the following lines of code for summary stats of the delta A to report in the paper. These summary stats are presented in the paper because it is a time-invariant predictor in the second iteration of the conditional growth curve model
hist(unique(Downsel_HRV_Perf_TTU_Long_AllNeedTimeSubIDs$A_ExtremeCorrect_DeltaB1vsB3))
```

```
boxplot(unique(Downsel_HRV_Perf_TTU_Long_AllNeedTimeSubIDs$A_ExtremeCorrect_DeltaB1vsB3))
```

```
boxplot.stats(unique(Downsel_HRV_Perf_TTU_Long_AllNeedTimeSubIDs$A_ExtremeCorrect_DeltaB1vsB3))
```

```
## $stats
## [1] -0.0458 -0.0051  0.0201  0.0452  0.0808
## 
## $n
## [1] 31
## 
## $conf
## [1] 0.005826048 0.034373952
## 
## $out
## [1] 0.1738
```

```
mean(unique(Downsel_HRV_Perf_TTU_Long_AllNeedTimeSubIDs$A_ExtremeCorrect_DeltaB1vsB3))
```

```
## [1] 0.02372581
```

```
sd(unique(Downsel_HRV_Perf_TTU_Long_AllNeedTimeSubIDs$A_ExtremeCorrect_DeltaB1vsB3))
```

```
## [1] 0.04260933
```

```
#center the performance metrics on their mean in order to make the moderating effects (if they are present) more interpretable
Downsel_HRV_Perf_TTU_Long_AllNeedTimeSubIDs["A_ExtremeCorrect_DeltaB1vsB3_MeanCenter"]<-Downsel_HRV_Perf_TTU_Long_AllNeedTimeSubIDs$A_ExtremeCorrect_DeltaB1vsB3-mean(unique(Downsel_HRV_Perf_TTU_Long_AllNeedTimeSubIDs$A_ExtremeCorrect_DeltaB1vsB3)) #unique is fine here because there are 31 unique delta A (between Block 1 and Block 3 values)
```

## Build conditional growth curve models to assess the moderation effect of vigilance task performance on cardiac vagal tone

### Test how a change in A from Block 1 to Block 3 moderates the time slopes of the unconditional growth curve model

The first conditional growth curve model answers the following
question: Do cardiac vagal tone trends over time inform the vigilance
decrement observed in the 30 minute vigilance task?

We add delta A (and all the other performance measures in other
iterations of conditional growth curve modeling) as a time-invariant
predictor to all time slopes as our research goal was to explore the
relation vigilance performance had with cardiac vagal tone, as it is a
proxy measure for Pre-frontal cortex (PFC) activity, which is related to
the several theories surrounding the vigilance decrement.

Procedural note: We first need to build the unconditional growth
curve model with the same dataset being used to then build the
conditional growth curve models.

```
SameData.ln.Fix.78.910.25.Rand.12.57quad.89<-lmer(data=Downsel_HRV_Perf_TTU_Long_AllNeedTimeSubIDs, REML=TRUE, 
                                                    formula=ln_RMSSD_ms~(1+slope12+slope25+slope57+I(slope57^2)+slope78+slope89+slope910)+
                                                    (1+slope12+slope57+I(slope57^2)+slope89|New_ID), control = lmerControl(optimizer = "bobyqa"))
 
#build the theoretically motivated conditional growth curve model and review the fixed effects of the model
AprimeDeltaB1vsB3.ln.Fix.78.910.25.Rand.12.57quad.89<-lmer(data=Downsel_HRV_Perf_TTU_Long_AllNeedTimeSubIDs, REML=TRUE, 
                                                              formula=ln_RMSSD_ms~1+slope12+slope25+slope57+I(slope57^2)+slope78+slope89+slope910+
                                                                        A_ExtremeCorrect_DeltaB1vsB3_MeanCenter+slope12:A_ExtremeCorrect_DeltaB1vsB3_MeanCenter+
                                                                        slope25:A_ExtremeCorrect_DeltaB1vsB3_MeanCenter+slope57:A_ExtremeCorrect_DeltaB1vsB3_MeanCenter+
                                                                        I(slope57^2):A_ExtremeCorrect_DeltaB1vsB3_MeanCenter+slope78:A_ExtremeCorrect_DeltaB1vsB3_MeanCenter+
                                                                        slope89:A_ExtremeCorrect_DeltaB1vsB3_MeanCenter+slope910:A_ExtremeCorrect_DeltaB1vsB3_MeanCenter+
                                                                      (1+slope12+slope57+I(slope57^2)+slope89|New_ID), control=lmerControl(optimizer = "bobyqa"))

fixef(AprimeDeltaB1vsB3.ln.Fix.78.910.25.Rand.12.57quad.89);length(fixef(AprimeDeltaB1vsB3.ln.Fix.78.910.25.Rand.12.57quad.89))
```

```
##                                          (Intercept) 
##                                          3.300604483 
##                                              slope12 
##                                          0.059623786 
##                                              slope25 
##                                          0.006734447 
##                                              slope57 
##                                          0.013517228 
##                                         I(slope57^2) 
##                                          0.019485180 
##                                              slope78 
##                                          0.006340490 
##                                              slope89 
##                                          0.046729394 
##                                             slope910 
##                                          0.030553171 
##              A_ExtremeCorrect_DeltaB1vsB3_MeanCenter 
##                                          4.011593322 
##      slope12:A_ExtremeCorrect_DeltaB1vsB3_MeanCenter 
##                                         -2.971014341 
##      slope25:A_ExtremeCorrect_DeltaB1vsB3_MeanCenter 
##                                          0.079133936 
##      slope57:A_ExtremeCorrect_DeltaB1vsB3_MeanCenter 
##                                          1.871041501 
## I(slope57^2):A_ExtremeCorrect_DeltaB1vsB3_MeanCenter 
##                                         -0.538488923 
##      slope78:A_ExtremeCorrect_DeltaB1vsB3_MeanCenter 
##                                         -1.522397489 
##      slope89:A_ExtremeCorrect_DeltaB1vsB3_MeanCenter 
##                                          0.317319237 
##     slope910:A_ExtremeCorrect_DeltaB1vsB3_MeanCenter 
##                                         -0.378379138
```

```
## [1] 16
```

DF=8 Wald test for all slopes moderated by delta A of Block 1 and
Block 3.

```
contestMD(AprimeDeltaB1vsB3.ln.Fix.78.910.25.Rand.12.57quad.89, ddf="Satterthwaite", L=rbind(c(0,0,0,0,0,0,0,0,1,0,0,0,0,0,0,0),
                                                                                             c(0,0,0,0,0,0,0,0,0,1,0,0,0,0,0,0),
                                                                                             c(0,0,0,0,0,0,0,0,0,0,1,0,0,0,0,0),
                                                                                             c(0,0,0,0,0,0,0,0,0,0,0,1,0,0,0,0),
                                                                                             c(0,0,0,0,0,0,0,0,0,0,0,0,1,0,0,0),
                                                                                             c(0,0,0,0,0,0,0,0,0,0,0,0,0,1,0,0),
                                                                                             c(0,0,0,0,0,0,0,0,0,0,0,0,0,0,1,0),
                                                                                             c(0,0,0,0,0,0,0,0,0,0,0,0,0,0,0,1)))
```

```
##      Sum Sq    Mean Sq NumDF    DenDF  F value     Pr(>F)
## 1 0.1811667 0.02264583     8 51.19504 2.318549 0.03318424
```

F(8,51.19504)=2.318549, p=0.03318424, suggesting delta A is a
significant moderator of cardiac vagal tone trends during a vigilance
task, break, and post-break vigilance task.

```
summary(AprimeDeltaB1vsB3.ln.Fix.78.910.25.Rand.12.57quad.89, ddf="Satterthwaite"); llikAIC(AprimeDeltaB1vsB3.ln.Fix.78.910.25.Rand.12.57quad.89, chkREML=FALSE)
```

```
## Linear mixed model fit by REML. t-tests use Satterthwaite's method [
## lmerModLmerTest]
## Formula: ln_RMSSD_ms ~ 1 + slope12 + slope25 + slope57 + I(slope57^2) +  
##     slope78 + slope89 + slope910 + A_ExtremeCorrect_DeltaB1vsB3_MeanCenter +  
##     slope12:A_ExtremeCorrect_DeltaB1vsB3_MeanCenter + slope25:A_ExtremeCorrect_DeltaB1vsB3_MeanCenter +  
##     slope57:A_ExtremeCorrect_DeltaB1vsB3_MeanCenter + I(slope57^2):A_ExtremeCorrect_DeltaB1vsB3_MeanCenter +  
##     slope78:A_ExtremeCorrect_DeltaB1vsB3_MeanCenter + slope89:A_ExtremeCorrect_DeltaB1vsB3_MeanCenter +  
##     slope910:A_ExtremeCorrect_DeltaB1vsB3_MeanCenter + (1 + slope12 +  
##     slope57 + I(slope57^2) + slope89 | New_ID)
##    Data: Downsel_HRV_Perf_TTU_Long_AllNeedTimeSubIDs
## Control: lmerControl(optimizer = "bobyqa")
## 
## REML criterion at convergence: -233.2
## 
## Scaled residuals: 
##     Min      1Q  Median      3Q     Max 
## -2.3581 -0.5324  0.0288  0.4910  2.3677 
## 
## Random effects:
##  Groups   Name         Variance Std.Dev. Corr                   
##  New_ID   (Intercept)  0.174092 0.41724                         
##           slope12      0.067177 0.25919  -0.28                  
##           slope57      0.096670 0.31092   0.02 -0.64            
##           I(slope57^2) 0.014765 0.12151  -0.11  0.67 -0.98      
##           slope89      0.020194 0.14211   0.39 -0.20  0.00 -0.16
##  Residual              0.009767 0.09883                         
## Number of obs: 310, groups:  New_ID, 31
## 
## Fixed effects:
##                                                        Estimate Std. Error
## (Intercept)                                            3.300604   0.077013
## slope12                                                0.059624   0.051987
## slope25                                                0.006734   0.007938
## slope57                                                0.013517   0.070382
## I(slope57^2)                                           0.019485   0.030418
## slope78                                                0.006340   0.025103
## slope89                                                0.046729   0.035799
## slope910                                               0.030553   0.025103
## A_ExtremeCorrect_DeltaB1vsB3_MeanCenter                4.011593   1.837291
## slope12:A_ExtremeCorrect_DeltaB1vsB3_MeanCenter       -2.971014   1.240249
## slope25:A_ExtremeCorrect_DeltaB1vsB3_MeanCenter        0.079134   0.189381
## slope57:A_ExtremeCorrect_DeltaB1vsB3_MeanCenter        1.871042   1.679113
## I(slope57^2):A_ExtremeCorrect_DeltaB1vsB3_MeanCenter  -0.538489   0.725686
## slope78:A_ExtremeCorrect_DeltaB1vsB3_MeanCenter       -1.522397   0.598874
## slope89:A_ExtremeCorrect_DeltaB1vsB3_MeanCenter        0.317319   0.854057
## slope910:A_ExtremeCorrect_DeltaB1vsB3_MeanCenter      -0.378379   0.598874
##                                                              df t value
## (Intercept)                                           28.999952  42.858
## slope12                                               32.280774   1.147
## slope25                                              149.007817   0.848
## slope57                                               33.663823   0.192
## I(slope57^2)                                          34.280543   0.641
## slope78                                              149.007816   0.253
## slope89                                               49.956281   1.305
## slope910                                             149.007817   1.217
## A_ExtremeCorrect_DeltaB1vsB3_MeanCenter               28.999951   2.183
## slope12:A_ExtremeCorrect_DeltaB1vsB3_MeanCenter       32.280813  -2.395
## slope25:A_ExtremeCorrect_DeltaB1vsB3_MeanCenter      149.007817   0.418
## slope57:A_ExtremeCorrect_DeltaB1vsB3_MeanCenter       33.663827   1.114
## I(slope57^2):A_ExtremeCorrect_DeltaB1vsB3_MeanCenter  34.280584  -0.742
## slope78:A_ExtremeCorrect_DeltaB1vsB3_MeanCenter      149.007816  -2.542
## slope89:A_ExtremeCorrect_DeltaB1vsB3_MeanCenter       49.956282   0.372
## slope910:A_ExtremeCorrect_DeltaB1vsB3_MeanCenter     149.007817  -0.632
##                                                      Pr(>|t|)    
## (Intercept)                                            <2e-16 ***
## slope12                                                0.2599    
## slope25                                                0.3976    
## slope57                                                0.8489    
## I(slope57^2)                                           0.5261    
## slope78                                                0.8009    
## slope89                                                0.1978    
## slope910                                               0.2255    
## A_ExtremeCorrect_DeltaB1vsB3_MeanCenter                0.0372 *  
## slope12:A_ExtremeCorrect_DeltaB1vsB3_MeanCenter        0.0226 *  
## slope25:A_ExtremeCorrect_DeltaB1vsB3_MeanCenter        0.6767    
## slope57:A_ExtremeCorrect_DeltaB1vsB3_MeanCenter        0.2730    
## I(slope57^2):A_ExtremeCorrect_DeltaB1vsB3_MeanCenter   0.4631    
## slope78:A_ExtremeCorrect_DeltaB1vsB3_MeanCenter        0.0120 *  
## slope89:A_ExtremeCorrect_DeltaB1vsB3_MeanCenter        0.7118    
## slope910:A_ExtremeCorrect_DeltaB1vsB3_MeanCenter       0.5285    
## ---
## Signif. codes:  0 '***' 0.001 '**' 0.01 '*' 0.05 '.' 0.1 ' ' 1
```

```
## $logLik
## 'log Lik.' 116.6067 (df=32)
## 
## $AICtab
##        AIC        BIC     logLik   deviance   df.resid 
## -169.21338  -49.64307  116.60669 -233.21338  278.00000
```

Simple slope for intercept based on -1SD, Mean, and +1SD of *delta
A* from Block 1 to Block 3 (mean=0.0237 and sd=0.0426)“).

Change in Intercept when *delta A* from Block 1 to Block 3 is
1SD *above* mean.

```
contest1D(AprimeDeltaB1vsB3.ln.Fix.78.910.25.Rand.12.57quad.89, ddf="Satterthwaite", L=c(0,0,0,0,0,0,0,0,0.0426,0,0,0,0,0,0,0))
```

```
##    Estimate Std. Error       df  t value   Pr(>|t|)
## 1 0.1708939 0.07826858 28.99995 2.183429 0.03724227
```

Change in slope12 when *delta A* from Block 1 to Block 3 is
1SD *above* mean.

```
contest1D(AprimeDeltaB1vsB3.ln.Fix.78.910.25.Rand.12.57quad.89, ddf="Satterthwaite", L=c(0,0,0,0,0,0,0,0,0,0.426,0,0,0,0,0,0))
```

```
##    Estimate Std. Error       df   t value   Pr(>|t|)
## 1 -1.265652  0.5283461 32.28081 -2.395498 0.02256481
```

Change in slope78 when *delta A* from Block 1 to Block 3 is
1SD *above* mean.

```
contest1D(AprimeDeltaB1vsB3.ln.Fix.78.910.25.Rand.12.57quad.89, ddf="Satterthwaite", L=c(0,0,0,0,0,0,0,0,0,0,0,0,0,0.426,0,0))
```

```
##     Estimate Std. Error       df   t value   Pr(>|t|)
## 1 -0.6485413  0.2551204 149.0078 -2.542099 0.01204018
```

Save yhat and correlation of yhat with y - Need to do this in order
to compare how much more variance in accounted for in comparison to the
unconditional growth curve model this conditional model is based on.

```
Downsel_HRV_Perf_TTU_Long_AllNeedTimeSubIDs$PredAprimeDeltaB1B3Mod = predict(AprimeDeltaB1vsB3.ln.Fix.78.910.25.Rand.12.57quad.89, re.form=NA) #we do not include random effects in this estimate because random effects are still error variances. We do not know why these fixed effects have significant variability (i.e., why they are random effects) so it does not behoove us to include them in predictions. re.form=NA tells the predict function not to use the random effects in the prediction
rPredAprimeDeltaB1B3Mod = cor.test(Downsel_HRV_Perf_TTU_Long_AllNeedTimeSubIDs$PredAprimeDeltaB1B3Mod, Downsel_HRV_Perf_TTU_Long_AllNeedTimeSubIDs$ln_RMSSD_ms, method="pearson")
```

Total R^2 of this conditional growth curve model, Total R^2 of the
unconditional growth curve model it is based on, and the difference
between the two Total R^2.

```
rPredAprimeDeltaB1B3Mod$estimate^2
```

```
##        cor 
## 0.08053645
```

```
Downsel_HRV_Perf_TTU_Long_AllNeedTimeSubIDs$PredUncondMod = predict(SameData.ln.Fix.78.910.25.Rand.12.57quad.89, re.form=NA)
rPredUncondMod = cor.test(Downsel_HRV_Perf_TTU_Long_AllNeedTimeSubIDs$PredUncondMod, Downsel_HRV_Perf_TTU_Long_AllNeedTimeSubIDs$ln_RMSSD_ms, method="pearson"); rPredUncondMod$estimate^2
```

```
##        cor 
## 0.03868931
```

```
rPredAprimeDeltaB1B3Mod$estimate^2-rPredUncondMod$estimate^2
```

```
##        cor 
## 0.04184714
```

### Plot individual trajectories to show the effects of adding delta A from Block 1 to Block 3 as a time-invariant predictor of the model

We use the ‘predict’ function here because we need full trajectories
for participants with incomplete data.

Create data in order to plot the estimated values of the best final
unconditional model.

```
CondPred_df<-with(Downsel_HRV_Perf_TTU_Long_AllNeedTimeSubIDs, expand.grid(New_ID=unique(New_ID), Timepoint=unique(Timepoint),
                                                                                                    Avg_A_ExtremeCorrect_DeltaB1vsB3_Centered=mean(unique(Downsel_HRV_Perf_TTU_Long_AllNeedTimeSubIDs$A_ExtremeCorrect_DeltaB1vsB3_MeanCenter)),
                                                                                                    SDAboveAvg_A_ExtremeCorrect_DeltaB1vsB3_Centered=sd(unique(Downsel_HRV_Perf_TTU_Long_AllNeedTimeSubIDs$A_ExtremeCorrect_DeltaB1vsB3_MeanCenter)),
                                                                                                    SDBelowAvg_A_ExtremeCorrect_DeltaB1vsB3_Centered=-1*(sd(unique(Downsel_HRV_Perf_TTU_Long_AllNeedTimeSubIDs$A_ExtremeCorrect_DeltaB1vsB3_MeanCenter)))))
#Build separate long format data frames in order to easily plot with ggplot
CondPred_long_df <- gather(CondPred_df, Type, A_ExtremeCorrect_DeltaB1vsB3_MeanCenter, Avg_A_ExtremeCorrect_DeltaB1vsB3_Centered, SDAboveAvg_A_ExtremeCorrect_DeltaB1vsB3_Centered, SDBelowAvg_A_ExtremeCorrect_DeltaB1vsB3_Centered)
#Rename factor names from "Avg_A_ExtremeCorrect_DeltaB1vsB3_Centered" etc to "Mean" etc
CondPred_long_df$Type<-factor(CondPred_long_df$Type)
levels(CondPred_long_df$Type) <- c("Mean decrement in 30 min vigilance task","Larger decrement, i.e., worse performance (1 SD above mean decrement)","Smaller decrement, i.e., better performance (1 SD below mean decrement)")
CondPred_long_df$Type<-factor(CondPred_long_df$Type, levels = c("Smaller decrement, i.e., better performance (1 SD below mean decrement)","Mean decrement in 30 min vigilance task","Larger decrement, i.e., worse performance (1 SD above mean decrement)"))
CondPred_long_df<-CondPred_long_df[order(CondPred_long_df$New_ID,CondPred_long_df$Timepoint,CondPred_long_df$Type),]

#Create a numeric time column that is centered on the baseline so modeling results are more interpretable
CondPred_long_df["Time"]<-as.numeric(CondPred_long_df$Timepoint)
CondPred_long_df$Time<-CondPred_long_df$Time-1
```

Create data frame and plot the predicted conditional growth curve
model.

Note: This will only work with balanced data.

```
#slope12, slope27, slope78, slope89, and slope910 leads to having intercepts at T1, T2, T7, T8, and T9, respectively
CondPred_long_df$slope12=CondPred_long_df$Time
CondPred_long_df$slope12[which(CondPred_long_df$Timepoint=="T1")]=0
CondPred_long_df$slope12[which(CondPred_long_df$Timepoint=="T2")]=1
CondPred_long_df$slope12[which(CondPred_long_df$Timepoint=="T3")]=1
CondPred_long_df$slope12[which(CondPred_long_df$Timepoint=="T4")]=1
CondPred_long_df$slope12[which(CondPred_long_df$Timepoint=="T5")]=1
CondPred_long_df$slope12[which(CondPred_long_df$Timepoint=="T6")]=1
CondPred_long_df$slope12[which(CondPred_long_df$Timepoint=="T7")]=1
CondPred_long_df$slope12[which(CondPred_long_df$Timepoint=="T8")]=1
CondPred_long_df$slope12[which(CondPred_long_df$Timepoint=="T9")]=1
CondPred_long_df$slope12[which(CondPred_long_df$Timepoint=="T10")]=1
CondPred_long_df$slope25=CondPred_long_df$Time
CondPred_long_df$slope25[which(CondPred_long_df$Timepoint=="T1")]=0
CondPred_long_df$slope25[which(CondPred_long_df$Timepoint=="T2")]=0
CondPred_long_df$slope25[which(CondPred_long_df$Timepoint=="T3")]=1
CondPred_long_df$slope25[which(CondPred_long_df$Timepoint=="T4")]=2
CondPred_long_df$slope25[which(CondPred_long_df$Timepoint=="T5")]=3
CondPred_long_df$slope25[which(CondPred_long_df$Timepoint=="T6")]=3
CondPred_long_df$slope25[which(CondPred_long_df$Timepoint=="T7")]=3
CondPred_long_df$slope25[which(CondPred_long_df$Timepoint=="T8")]=3
CondPred_long_df$slope25[which(CondPred_long_df$Timepoint=="T9")]=3
CondPred_long_df$slope25[which(CondPred_long_df$Timepoint=="T10")]=3
CondPred_long_df$slope57=CondPred_long_df$Time
CondPred_long_df$slope57[which(CondPred_long_df$Timepoint=="T1")]=0
CondPred_long_df$slope57[which(CondPred_long_df$Timepoint=="T2")]=0
CondPred_long_df$slope57[which(CondPred_long_df$Timepoint=="T3")]=0
CondPred_long_df$slope57[which(CondPred_long_df$Timepoint=="T4")]=0
CondPred_long_df$slope57[which(CondPred_long_df$Timepoint=="T5")]=0
CondPred_long_df$slope57[which(CondPred_long_df$Timepoint=="T6")]=1
CondPred_long_df$slope57[which(CondPred_long_df$Timepoint=="T7")]=2
CondPred_long_df$slope57[which(CondPred_long_df$Timepoint=="T8")]=2
CondPred_long_df$slope57[which(CondPred_long_df$Timepoint=="T9")]=2
CondPred_long_df$slope57[which(CondPred_long_df$Timepoint=="T10")]=2
CondPred_long_df$slope78=CondPred_long_df$Time
CondPred_long_df$slope78[which(CondPred_long_df$Timepoint=="T1")]=0
CondPred_long_df$slope78[which(CondPred_long_df$Timepoint=="T2")]=0
CondPred_long_df$slope78[which(CondPred_long_df$Timepoint=="T3")]=0
CondPred_long_df$slope78[which(CondPred_long_df$Timepoint=="T4")]=0
CondPred_long_df$slope78[which(CondPred_long_df$Timepoint=="T5")]=0
CondPred_long_df$slope78[which(CondPred_long_df$Timepoint=="T6")]=0
CondPred_long_df$slope78[which(CondPred_long_df$Timepoint=="T7")]=0
CondPred_long_df$slope78[which(CondPred_long_df$Timepoint=="T8")]=1
CondPred_long_df$slope78[which(CondPred_long_df$Timepoint=="T9")]=1
CondPred_long_df$slope78[which(CondPred_long_df$Timepoint=="T10")]=1
CondPred_long_df$slope89=CondPred_long_df$Time
CondPred_long_df$slope89[which(CondPred_long_df$Timepoint=="T1")]=0
CondPred_long_df$slope89[which(CondPred_long_df$Timepoint=="T2")]=0
CondPred_long_df$slope89[which(CondPred_long_df$Timepoint=="T3")]=0
CondPred_long_df$slope89[which(CondPred_long_df$Timepoint=="T4")]=0
CondPred_long_df$slope89[which(CondPred_long_df$Timepoint=="T5")]=0
CondPred_long_df$slope89[which(CondPred_long_df$Timepoint=="T6")]=0
CondPred_long_df$slope89[which(CondPred_long_df$Timepoint=="T7")]=0
CondPred_long_df$slope89[which(CondPred_long_df$Timepoint=="T8")]=0
CondPred_long_df$slope89[which(CondPred_long_df$Timepoint=="T9")]=1
CondPred_long_df$slope89[which(CondPred_long_df$Timepoint=="T10")]=1
CondPred_long_df$slope910=CondPred_long_df$Time
CondPred_long_df$slope910[which(CondPred_long_df$Timepoint=="T1")]=0
CondPred_long_df$slope910[which(CondPred_long_df$Timepoint=="T2")]=0
CondPred_long_df$slope910[which(CondPred_long_df$Timepoint=="T3")]=0
CondPred_long_df$slope910[which(CondPred_long_df$Timepoint=="T4")]=0
CondPred_long_df$slope910[which(CondPred_long_df$Timepoint=="T5")]=0
CondPred_long_df$slope910[which(CondPred_long_df$Timepoint=="T6")]=0
CondPred_long_df$slope910[which(CondPred_long_df$Timepoint=="T7")]=0
CondPred_long_df$slope910[which(CondPred_long_df$Timepoint=="T8")]=0
CondPred_long_df$slope910[which(CondPred_long_df$Timepoint=="T9")]=0
CondPred_long_df$slope910[which(CondPred_long_df$Timepoint=="T10")]=1

#Use the predictions from this conditional growth curve model to build the graphs
CondPred_long_df["Pred"]<-predict(AprimeDeltaB1vsB3.ln.Fix.78.910.25.Rand.12.57quad.89, CondPred_long_df, re.form=NA)
CondPred_long_df$Timepoint<-factor(CondPred_long_df$Timepoint, levels = c("T1","T2","T3","T4","T5","T6","T7","T8","T9","T10")) 
CondPred_long_df$Type<-factor(CondPred_long_df$Type, levels = c("Smaller decrement, i.e., better performance (1 SD below mean decrement)",
                                                                "Mean decrement in 30 min vigilance task",
                                                                "Larger decrement, i.e., worse performance (1 SD above mean decrement)")) 

graph_PerfCondGrowCur_AprimeDeltaB1vsB3<-ggplot(CondPred_long_df,aes(x=Timepoint,y=Pred,group=Type)) + 
                                              geom_line(aes(linetype=Type,colour=Type,linewidth=Type)) + 
                                                scale_linetype_manual(labels = ~ stringr::str_wrap(.x, width = 35), values=c("twodash", "solid", "dotted")) + 
                                                scale_colour_manual(labels = ~ stringr::str_wrap(.x, width = 35),values=c("black","black","black")) + 
                                                scale_discrete_manual("linewidth",labels = ~ stringr::str_wrap(.x, width = 35),values=c(5,5,5)) + 
                                              geom_point(aes(colour=Type,shape=Type,size=Type)) + 
                                                scale_colour_manual(labels = ~ stringr::str_wrap(.x, width = 35),values=c("black","black","black")) + 
                                                scale_shape_manual(labels = ~ stringr::str_wrap(.x, width = 35),values=c(19,15,17)) + 
                                                scale_size_manual(labels = ~ stringr::str_wrap(.x, width = 35),values=c(10,10,10)) + 
                                              labs(y="Predicted ln(RMSSD)", x="Time period") +  
                                              ggtitle("Predicted ln(RMSSD) across all time periods for various vigilance decrements (i.e., values of delta A)") +
                                              theme(axis.ticks.x=element_blank(),
                                                legend.position = "right", 
                                                legend.key.width = unit(3, 'cm'),
                                                legend.key = element_blank(),
                                                legend.text=element_text(margin = margin(t=10),size=30),
                                               # plot.title = element_text(hjust = 0.5),
                                                panel.grid.major = element_blank(),
                                                panel.grid.minor = element_blank(),
                                                panel.background = element_blank(),
                                                axis.line.x = element_line(colour = "black"),
                                                axis.line.y = element_line(colour = "black"),
                                                text=element_text(color="black", size=50),
                                                axis.text=element_text(color="black", size=40),
                                                strip.background = element_rect(color = "black",fill = "white"))
graph_PerfCondGrowCur_AprimeDeltaB1vsB3
```

Summary: This plot shows that those with a smaller vigilance task
decrement (i.e., performance does not decline as much during the
vigilance task) see an initial decrease in their cardiac vagal tone upon
starting the vigilance task, suggesting an initial decrease in
cognitive/emotional control. However, cardiac vagal tone is then
predicted to increase as the vigilance task progresses, i.e.,
cognitive/emotional control increases. It then decreases again during
the break and then increases a final time during the post-break
vigilance task. The opposite trend is predicted for participant’s who
have a vigilance decrement that is 1 standard deviation above the mean
vigilance decrement. Overall, it seems that those who do not decline in
performance as steeply (or at all) are predicted to have more
cognitive/emotional control at baseline and during the vigilance task.
They also seem to decrease their control when not completing the
task.

### Test how a change in median response time of hits (HitRTmd) from Block 1 to Block 3 moderates the time slopes of the unconditional growth curve model

Calculate delta of HitRTmd between Block 1 and Block 3 to test if it
significantly moderates the time slopes of the unconditional growth
curve model.

```
Downsel_HRV_Perf_TTU_Long_AllNeedTimeSubIDs["HitRTmd_DeltaB1vsB3"]<-(Downsel_HRV_Perf_TTU_Long_AllNeedTimeSubIDs$HitRTmdB1)-(Downsel_HRV_Perf_TTU_Long_AllNeedTimeSubIDs$HitRTmdB3)

length(unique(Downsel_HRV_Perf_TTU_Long_AllNeedTimeSubIDs$HitRTmd_DeltaB1vsB3)) == 31 #need the unique # of participants so that the standard deviation calculation is correct. the length == 31 because we need everyone's value represented, even if it is a repeat of another's value
```

```
## [1] FALSE
```

```
#if the statement above is TRUE, run the following
hist(unique(Downsel_HRV_Perf_TTU_Long_AllNeedTimeSubIDs$HitRTmd_DeltaB1vsB3))
```

```
boxplot(unique(Downsel_HRV_Perf_TTU_Long_AllNeedTimeSubIDs$HitRTmd_DeltaB1vsB3))
```

```
boxplot.stats(unique(Downsel_HRV_Perf_TTU_Long_AllNeedTimeSubIDs$HitRTmd_DeltaB1vsB3))
```

```
## $stats
## [1] -216.00  -85.00  -36.75    7.50   86.00
## 
## $n
## [1] 30
## 
## $conf
## [1] -63.43322 -10.06678
## 
## $out
## [1] -441  193
```

```
mean(unique(Downsel_HRV_Perf_TTU_Long_AllNeedTimeSubIDs$HitRTmd_DeltaB1vsB3))
```

```
## [1] -49.6
```

```
sd(unique(Downsel_HRV_Perf_TTU_Long_AllNeedTimeSubIDs$HitRTmd_DeltaB1vsB3))
```

```
## [1] 113.3657
```

```
min(unique(Downsel_HRV_Perf_TTU_Long_AllNeedTimeSubIDs$HitRTmd_DeltaB1vsB3))
```

```
## [1] -441
```

```
max(unique(Downsel_HRV_Perf_TTU_Long_AllNeedTimeSubIDs$HitRTmd_DeltaB1vsB3))
```

```
## [1] 193
```

```
sort(unique(Downsel_HRV_Perf_TTU_Long_AllNeedTimeSubIDs$HitRTmd_DeltaB1vsB3))
```

```
##  [1] -441.0 -216.0 -182.5 -181.0 -175.0 -129.0  -91.0  -85.0  -70.0  -57.0
## [11]  -54.0  -49.0  -46.5  -46.0  -37.5  -36.0  -34.5  -25.5   -7.0   -2.0
## [21]    0.0    3.0    7.5   17.0   23.0   27.0   43.0   78.0   86.0  193.0
```

```
#center the performance metrics on their mean in order to make the moderating effects (if they are present) more interpretable
Downsel_HRV_Perf_TTU_Long_AllNeedTimeSubIDs["HitRTmd_DeltaB1vsB3_MeanCenter"]<-Downsel_HRV_Perf_TTU_Long_AllNeedTimeSubIDs$HitRTmd_DeltaB1vsB3-mean(unique(Downsel_HRV_Perf_TTU_Long_AllNeedTimeSubIDs$HitRTmd_DeltaB1vsB3))
```

```
#add in the median response time of hits (HitRTmd) as predictors to all time slopes
HitRTmd_DeltaB1vsB3.ln.Fix.78.910.25.Rand.12.57quad.89<-lmer(data=Downsel_HRV_Perf_TTU_Long_AllNeedTimeSubIDs, REML=TRUE, 
                                                              formula=ln_RMSSD_ms~1+slope12+slope25+slope57+I(slope57^2)+slope78+slope89+slope910+
                                                                        HitRTmd_DeltaB1vsB3_MeanCenter+slope12:HitRTmd_DeltaB1vsB3_MeanCenter+
                                                                        slope25:HitRTmd_DeltaB1vsB3_MeanCenter+slope57:HitRTmd_DeltaB1vsB3_MeanCenter+
                                                                        I(slope57^2):HitRTmd_DeltaB1vsB3_MeanCenter+slope78:HitRTmd_DeltaB1vsB3_MeanCenter+
                                                                        slope89:HitRTmd_DeltaB1vsB3_MeanCenter+slope910:HitRTmd_DeltaB1vsB3_MeanCenter+
                                                                      (1+slope12+slope57+I(slope57^2)+slope89|New_ID), control=lmerControl(optimizer = "optimx", optCtrl=list(method='L-BFGS-B'))) #convergence error not a major concern
```

```
## Warning in optwrap(optimizer, devfun, getStart(start, rho$pp), lower =
## rho$lower, : convergence code 1 from optimx: none
```

```
## Warning in checkConv(attr(opt, "derivs"), opt$par, ctrl = control$checkConv, :
## Model failed to converge with max|grad| = 0.00782917 (tol = 0.002, component 1)
```

```
fixef(HitRTmd_DeltaB1vsB3.ln.Fix.78.910.25.Rand.12.57quad.89);length(fixef(HitRTmd_DeltaB1vsB3.ln.Fix.78.910.25.Rand.12.57quad.89))
```

```
##                                 (Intercept) 
##                                3.300749e+00 
##                                     slope12 
##                                5.959636e-02 
##                                     slope25 
##                                6.724262e-03 
##                                     slope57 
##                                1.362179e-02 
##                                I(slope57^2) 
##                                1.943695e-02 
##                                     slope78 
##                                6.338628e-03 
##                                     slope89 
##                                4.677572e-02 
##                                    slope910 
##                                3.053555e-02 
##              HitRTmd_DeltaB1vsB3_MeanCenter 
##                               -1.244337e-03 
##      slope12:HitRTmd_DeltaB1vsB3_MeanCenter 
##                                2.361382e-04 
##      slope25:HitRTmd_DeltaB1vsB3_MeanCenter 
##                                8.769820e-05 
##      slope57:HitRTmd_DeltaB1vsB3_MeanCenter 
##                               -9.004185e-04 
## I(slope57^2):HitRTmd_DeltaB1vsB3_MeanCenter 
##                                4.152933e-04 
##      slope78:HitRTmd_DeltaB1vsB3_MeanCenter 
##                                1.603526e-05 
##      slope89:HitRTmd_DeltaB1vsB3_MeanCenter 
##                               -3.988958e-04 
##     slope910:HitRTmd_DeltaB1vsB3_MeanCenter 
##                                1.517338e-04
```

```
## [1] 16
```

DF=8 Wald test for all slopes moderated by the median response time
of hits (HitRTmd).

```
contestMD(HitRTmd_DeltaB1vsB3.ln.Fix.78.910.25.Rand.12.57quad.89, ddf="Satterthwaite", L=rbind(c(0,0,0,0,0,0,0,0,1,0,0,0,0,0,0,0),
                                                                                                c(0,0,0,0,0,0,0,0,0,1,0,0,0,0,0,0),
                                                                                                c(0,0,0,0,0,0,0,0,0,0,1,0,0,0,0,0),
                                                                                                c(0,0,0,0,0,0,0,0,0,0,0,1,0,0,0,0),
                                                                                                c(0,0,0,0,0,0,0,0,0,0,0,0,1,0,0,0),
                                                                                                c(0,0,0,0,0,0,0,0,0,0,0,0,0,1,0,0),
                                                                                                c(0,0,0,0,0,0,0,0,0,0,0,0,0,0,1,0),
                                                                                                c(0,0,0,0,0,0,0,0,0,0,0,0,0,0,0,1)))
```

```
##       Sum Sq     Mean Sq NumDF    DenDF   F value    Pr(>F)
## 1 0.07261688 0.009077111     8 51.45125 0.8985654 0.5247306
```

F(8,51.19504)=0.8985654, p=0.5247306, suggesting the change in median
response time of hits (HitRTmd) between Block 1 and Block 3 is not a
significant moderator of cardiac vagal tone trends during a vigilance
task, break, or post-break vigilance task.

### Test how the change in A between Block 3 and Block 4 moderates the time slopes associated with Block 3, the break, and Block 4

Calculate delta A between Block 3 and Block 4 just as we did
above.

```
Downsel_HRV_Perf_TTU_Long_AllNeedTimeSubIDs["A_ExtremeCorrect_DeltaB3vsB4"]<-(Downsel_HRV_Perf_TTU_Long_AllNeedTimeSubIDs$Pre_Block3_A_ExtremeCorrect)-(Downsel_HRV_Perf_TTU_Long_AllNeedTimeSubIDs$Post_Block4_A_ExtremeCorrect)

#summary stats of the performance data that will be a time-invariant predictor in the performance conditional growth curve model
hist(unique(Downsel_HRV_Perf_TTU_Long_AllNeedTimeSubIDs$A_ExtremeCorrect_DeltaB3vsB4))
```

```
boxplot(unique(Downsel_HRV_Perf_TTU_Long_AllNeedTimeSubIDs$A_ExtremeCorrect_DeltaB3vsB4))
```

```
boxplot.stats(unique(Downsel_HRV_Perf_TTU_Long_AllNeedTimeSubIDs$A_ExtremeCorrect_DeltaB3vsB4))
```

```
## $stats
## [1] -0.07050 -0.02325 -0.00130  0.01980  0.05020
## 
## $n
## [1] 31
## 
## $conf
## [1] -0.01351657  0.01091657
## 
## $out
## [1] 0.0916
```

```
mean(unique(Downsel_HRV_Perf_TTU_Long_AllNeedTimeSubIDs$A_ExtremeCorrect_DeltaB3vsB4))
```

```
## [1] -0.001283871
```

```
sd(unique(Downsel_HRV_Perf_TTU_Long_AllNeedTimeSubIDs$A_ExtremeCorrect_DeltaB3vsB4))
```

```
## [1] 0.03457476
```

```
min(unique(Downsel_HRV_Perf_TTU_Long_AllNeedTimeSubIDs$A_ExtremeCorrect_DeltaB3vsB4))
```

```
## [1] -0.0705
```

```
max(unique(Downsel_HRV_Perf_TTU_Long_AllNeedTimeSubIDs$A_ExtremeCorrect_DeltaB3vsB4))
```

```
## [1] 0.0916
```

```
sort(unique(Downsel_HRV_Perf_TTU_Long_AllNeedTimeSubIDs$A_ExtremeCorrect_DeltaB3vsB4))
```

```
##  [1] -0.0705 -0.0634 -0.0451 -0.0402 -0.0377 -0.0356 -0.0294 -0.0245 -0.0220
## [10] -0.0182 -0.0148 -0.0108 -0.0060 -0.0052 -0.0051 -0.0013  0.0029  0.0036
## [19]  0.0056  0.0057  0.0071  0.0142  0.0148  0.0248  0.0300  0.0326  0.0335
## [28]  0.0337  0.0397  0.0502  0.0916
```

```
#center the performance metrics on their mean in order to make the moderating effects (if they are present) more interpretable
Downsel_HRV_Perf_TTU_Long_AllNeedTimeSubIDs["A_ExtremeCorrect_DeltaB3vsB4_MeanCenter"]<-Downsel_HRV_Perf_TTU_Long_AllNeedTimeSubIDs$A_ExtremeCorrect_DeltaB3vsB4-mean(unique(Downsel_HRV_Perf_TTU_Long_AllNeedTimeSubIDs$A_ExtremeCorrect_DeltaB3vsB4))

#build the theoretically motivated conditional growth curve model for performance differences between Block 3 and Block 4 (only interact with piecewise slopes that are touching the break)
AprimeDeltaB3vsB4CertainSlopes.ln.Fix.78.910.25.Rand.12.57quad.89<-lmer(data=Downsel_HRV_Perf_TTU_Long_AllNeedTimeSubIDs, REML=TRUE, 
                                                              formula=ln_RMSSD_ms~1+slope12+slope25+slope57+I(slope57^2)+slope78+slope89+slope910+
                                                                        A_ExtremeCorrect_DeltaB3vsB4+slope57:A_ExtremeCorrect_DeltaB3vsB4+
                                                                        I(slope57^2):A_ExtremeCorrect_DeltaB3vsB4+slope78:A_ExtremeCorrect_DeltaB3vsB4+
                                                                        slope89:A_ExtremeCorrect_DeltaB3vsB4+slope910:A_ExtremeCorrect_DeltaB3vsB4+
                                                                      (1+slope12+slope57+I(slope57^2)+slope89|New_ID), control=lmerControl(optimizer="bobyqa")) #, optCtrl=list(method='L-BFGS-B'))) #"Nelder_Mead", optCtrl=list(maxfun=5e5)))
```

```
## Warning: Model failed to converge with 1 negative eigenvalue: -1.5e-01
```

```
#allFit(AprimeDeltaB3vsB4.ln.Fix.78.910.25.Rand.12.57quad.89)

fixef(AprimeDeltaB3vsB4CertainSlopes.ln.Fix.78.910.25.Rand.12.57quad.89);length(fixef(AprimeDeltaB3vsB4CertainSlopes.ln.Fix.78.910.25.Rand.12.57quad.89))
```

```
##                               (Intercept) 
##                               3.303075160 
##                                   slope12 
##                               0.059623786 
##                                   slope25 
##                               0.006734447 
##                                   slope57 
##                               0.011686311 
##                              I(slope57^2) 
##                               0.020244908 
##                                   slope78 
##                               0.008319241 
##                                   slope89 
##                               0.045471354 
##                                  slope910 
##                               0.030704617 
##              A_ExtremeCorrect_DeltaB3vsB4 
##                               1.924396312 
##      slope57:A_ExtremeCorrect_DeltaB3vsB4 
##                              -1.426090816 
## I(slope57^2):A_ExtremeCorrect_DeltaB3vsB4 
##                               0.591747515 
##      slope78:A_ExtremeCorrect_DeltaB3vsB4 
##                               1.541238159 
##      slope89:A_ExtremeCorrect_DeltaB3vsB4 
##                              -0.979880238 
##     slope910:A_ExtremeCorrect_DeltaB3vsB4 
##                               0.117959792
```

```
## [1] 14
```

DF=6 Wald test for slopes moderated by delta A between Block 3 and
Block 4 slopes.

```
contestMD(AprimeDeltaB3vsB4CertainSlopes.ln.Fix.78.910.25.Rand.12.57quad.89, ddf="Satterthwaite", L=rbind(c(0,0,0,0,0,0,0,0,1,0,0,0,0,0),
                                                                                                          c(0,0,0,0,0,0,0,0,0,1,0,0,0,0),
                                                                                                          c(0,0,0,0,0,0,0,0,0,0,1,0,0,0),
                                                                                                          c(0,0,0,0,0,0,0,0,0,0,0,1,0,0),
                                                                                                          c(0,0,0,0,0,0,0,0,0,0,0,0,1,0),
                                                                                                          c(0,0,0,0,0,0,0,0,0,0,0,0,0,1)))
```

```
##       Sum Sq    Mean Sq NumDF    DenDF  F value    Pr(>F)
## 1 0.08976112 0.01496019     6 52.51455 1.522221 0.1891529
```

F(6,52.51456)=1.522223, p=0.1891522, suggesting delta A between Block
3 and Block 4 is not a significant moderator of cardiac vagal tone
trends during a vigilance task, break, or post-break vigilance task.

### Test how the change in median response time of hits (HitRTmd) between Block 3 and Block 4 moderates the time slopes associated with Block 3, the break, and Block 4

```
Downsel_HRV_Perf_TTU_Long_AllNeedTimeSubIDs["HitRTmd_DeltaB3vsB4"]<-(Downsel_HRV_Perf_TTU_Long_AllNeedTimeSubIDs$HitRTmdB3)-(Downsel_HRV_Perf_TTU_Long_AllNeedTimeSubIDs$HitRTmdB4)
  
#summary stats of the performance data that will be a time-invariant predictor in the performance conditional growth curve model
hist(unique(Downsel_HRV_Perf_TTU_Long_AllNeedTimeSubIDs$HitRTmd_DeltaB3vsB4))
```

```
boxplot(unique(Downsel_HRV_Perf_TTU_Long_AllNeedTimeSubIDs$HitRTmd_DeltaB3vsB4))
```

```
boxplot.stats(unique(Downsel_HRV_Perf_TTU_Long_AllNeedTimeSubIDs$HitRTmd_DeltaB3vsB4))
```

```
## $stats
## [1] -173.50  -39.00    8.75   67.00  199.50
## 
## $n
## [1] 30
## 
## $conf
## [1] -21.82752  39.32752
## 
## $out
## [1] 302 245
```

```
mean(unique(Downsel_HRV_Perf_TTU_Long_AllNeedTimeSubIDs$HitRTmd_DeltaB3vsB4))
```

```
## [1] 25.1
```

```
sd(unique(Downsel_HRV_Perf_TTU_Long_AllNeedTimeSubIDs$HitRTmd_DeltaB3vsB4))
```

```
## [1] 107.1304
```

```
min(unique(Downsel_HRV_Perf_TTU_Long_AllNeedTimeSubIDs$HitRTmd_DeltaB3vsB4))
```

```
## [1] -173.5
```

```
max(unique(Downsel_HRV_Perf_TTU_Long_AllNeedTimeSubIDs$HitRTmd_DeltaB3vsB4))
```

```
## [1] 302
```

```
sort(unique(Downsel_HRV_Perf_TTU_Long_AllNeedTimeSubIDs$HitRTmd_DeltaB3vsB4))
```

```
##  [1] -173.5 -134.0 -132.0 -102.5  -77.0  -72.5  -41.0  -39.0   -9.0   -8.0
## [11]   -5.0   -0.5    2.0    8.0    8.5    9.0   19.0   28.0   37.0   46.5
## [21]   56.0   58.0   67.0   94.0  112.5  116.0  139.0  199.5  245.0  302.0
```

```
#center the performance metrics on their mean in order to make the moderating effects (if they are present) more interpretable
Downsel_HRV_Perf_TTU_Long_AllNeedTimeSubIDs["HitRTmd_DeltaB3vsB4_MeanCenter"]<-Downsel_HRV_Perf_TTU_Long_AllNeedTimeSubIDs$HitRTmd_DeltaB3vsB4-mean(unique(Downsel_HRV_Perf_TTU_Long_AllNeedTimeSubIDs$HitRTmd_DeltaB3vsB4))


#build the theoretically motivated conditional growth curve model for performance differences between Block 3 and Block 4 (only interact with piecewise slopes that are touching the break)
HitMedRTDeltaB3vsB4CertainSlopes.ln.Fix.78.910.25.Rand.12.57quad.89<-lmer(data=Downsel_HRV_Perf_TTU_Long_AllNeedTimeSubIDs, REML=TRUE, 
                                                              formula=ln_RMSSD_ms~1+slope12+slope25+slope57+I(slope57^2)+slope78+slope89+slope910+
                                                                        HitRTmd_DeltaB3vsB4+slope57:HitRTmd_DeltaB3vsB4+
                                                                        I(slope57^2):HitRTmd_DeltaB3vsB4+slope78:HitRTmd_DeltaB3vsB4+
                                                                        slope89:HitRTmd_DeltaB3vsB4+slope910:HitRTmd_DeltaB3vsB4+
                                                                      (1+slope12+slope57+I(slope57^2)+slope89|New_ID), control=lmerControl(optimizer="nlminbwrap")) #,optCtrl=list(method='L-BFGS-B'))) #"Nelder_Mead", optCtrl=list(maxfun=5e5)))
#allFit(HitMedRTDeltaB3vsB4CertainSlopes.ln.Fix.78.910.25.Rand.12.57quad.89)

fixef(HitMedRTDeltaB3vsB4CertainSlopes.ln.Fix.78.910.25.Rand.12.57quad.89);length(fixef(HitMedRTDeltaB3vsB4CertainSlopes.ln.Fix.78.910.25.Rand.12.57quad.89))
```

```
##                      (Intercept)                          slope12 
##                     3.274305e+00                     5.962379e-02 
##                          slope25                          slope57 
##                     6.734447e-03                     2.221998e-03 
##                     I(slope57^2)                          slope78 
##                     2.611142e-02                     7.746554e-03 
##                          slope89                         slope910 
##                     3.125160e-02                     3.183059e-02 
##              HitRTmd_DeltaB3vsB4      slope57:HitRTmd_DeltaB3vsB4 
##                     1.032006e-03                     4.432305e-04 
## I(slope57^2):HitRTmd_DeltaB3vsB4      slope78:HitRTmd_DeltaB3vsB4 
##                    -2.600172e-04                    -5.517464e-05 
##      slope89:HitRTmd_DeltaB3vsB4     slope910:HitRTmd_DeltaB3vsB4 
##                     6.073564e-04                    -5.012661e-05
```

```
## [1] 14
```

DF=6 Wald Test for all slopes moderated by delta A of Block 3 and
Block 4.

```
contestMD(HitMedRTDeltaB3vsB4CertainSlopes.ln.Fix.78.910.25.Rand.12.57quad.89, ddf="Satterthwaite", L=rbind(c(0,0,0,0,0,0,0,0,1,0,0,0,0,0),
                                                                                                            c(0,0,0,0,0,0,0,0,0,1,0,0,0,0),
                                                                                                            c(0,0,0,0,0,0,0,0,0,0,1,0,0,0),
                                                                                                            c(0,0,0,0,0,0,0,0,0,0,0,1,0,0),
                                                                                                            c(0,0,0,0,0,0,0,0,0,0,0,0,1,0),
                                                                                                            c(0,0,0,0,0,0,0,0,0,0,0,0,0,1)))
```

```
##      Sum Sq    Mean Sq NumDF   DenDF  F value    Pr(>F)
## 1 0.0748651 0.01247752     6 45.8959 1.228813 0.3092235
```

F(6,45.8959)=1.228813, p=0.3092235, suggesting the change in median
response time of hits (HitRTmd) from Block 3 to Block 4 is not a
significant moderator of cardiac vagal tone trends during a vigilance
task, break, or post-break vigilance task.

### Test how A in Block 4 moderates the time slopes associated with the break and Block 4

Summary stats of A in Block 4 (as reported in the journal
article).

```
hist(subset.data.frame(Downsel_HRV_Perf_TTU_Long_AllNeedTimeSubIDs,Downsel_HRV_Perf_TTU_Long_AllNeedTimeSubIDs$Timepoint=="T1")$Post_Block4_A_ExtremeCorrect)
```

```
boxplot(subset.data.frame(Downsel_HRV_Perf_TTU_Long_AllNeedTimeSubIDs,Downsel_HRV_Perf_TTU_Long_AllNeedTimeSubIDs$Timepoint=="T1")$Post_Block4_A_ExtremeCorrect)
```

```
boxplot.stats(subset.data.frame(Downsel_HRV_Perf_TTU_Long_AllNeedTimeSubIDs,Downsel_HRV_Perf_TTU_Long_AllNeedTimeSubIDs$Timepoint=="T1")$Post_Block4_A_ExtremeCorrect)
```

```
## $stats
## [1] 0.7819 0.8336 0.8683 0.9058 0.9936
## 
## $n
## [1] 31
## 
## $conf
## [1] 0.8478113 0.8887887
## 
## $out
## numeric(0)
```

```
mean(subset.data.frame(Downsel_HRV_Perf_TTU_Long_AllNeedTimeSubIDs,Downsel_HRV_Perf_TTU_Long_AllNeedTimeSubIDs$Timepoint=="T1")$Post_Block4_A_ExtremeCorrect)
```

```
## [1] 0.8777226
```

```
sd(subset.data.frame(Downsel_HRV_Perf_TTU_Long_AllNeedTimeSubIDs,Downsel_HRV_Perf_TTU_Long_AllNeedTimeSubIDs$Timepoint=="T1")$Post_Block4_A_ExtremeCorrect)
```

```
## [1] 0.05947071
```

```
min(subset.data.frame(Downsel_HRV_Perf_TTU_Long_AllNeedTimeSubIDs,Downsel_HRV_Perf_TTU_Long_AllNeedTimeSubIDs$Timepoint=="T1")$Post_Block4_A_ExtremeCorrect)
```

```
## [1] 0.7819
```

```
max(subset.data.frame(Downsel_HRV_Perf_TTU_Long_AllNeedTimeSubIDs,Downsel_HRV_Perf_TTU_Long_AllNeedTimeSubIDs$Timepoint=="T1")$Post_Block4_A_ExtremeCorrect)
```

```
## [1] 0.9936
```

```
#center the performance metrics on their mean in order to make the moderating effects (if they are present) more interpretable
Downsel_HRV_Perf_TTU_Long_AllNeedTimeSubIDs["Post_Block4_A_ExtremeCorrect_MeanCenter"]<-Downsel_HRV_Perf_TTU_Long_AllNeedTimeSubIDs$Post_Block4_A_ExtremeCorrect-mean(subset.data.frame(Downsel_HRV_Perf_TTU_Long_AllNeedTimeSubIDs,Downsel_HRV_Perf_TTU_Long_AllNeedTimeSubIDs$Timepoint=="T1")$Post_Block4_A_ExtremeCorrect)
```

```
#build the theoretically motivated conditional growth curve model for performance differences between Block 3 and Block 4 (only interact with piecewise slopes that are touching the break)
AprimeB4CertainSlopes.ln.Fix.78.910.25.Rand.12.57quad.89<-lmer(data=Downsel_HRV_Perf_TTU_Long_AllNeedTimeSubIDs, REML=TRUE, 
                                                                    formula=ln_RMSSD_ms~1+slope12+slope25+slope57+I(slope57^2)+slope78+slope89+slope910+
                                                                      Post_Block4_A_ExtremeCorrect+slope78:Post_Block4_A_ExtremeCorrect_MeanCenter+
                                                                      slope89:Post_Block4_A_ExtremeCorrect_MeanCenter+
                                                                      slope910:Post_Block4_A_ExtremeCorrect_MeanCenter+
                                                                      (1+slope12+slope57+I(slope57^2)+slope89|New_ID), 
                                                                      control=lmerControl(optimizer="nloptwrap", optCtrl=list(method='NLOPT_LN_BOBYQA')))
```

```
## Warning in checkConv(attr(opt, "derivs"), opt$par, ctrl = control$checkConv, :
## Model failed to converge with max|grad| = 0.00491392 (tol = 0.002, component 1)
```

```
                                                                        # optCtrl=list(method='L-BFGS-B'))) "Nelder_Mead", optCtrl=list(maxfun=5e5))
#allFit(AprimeB4CertainSlopes.ln.Fix.78.910.25.Rand.12.57quad.89) #allFit finds teh convergence issue is minor

fixef(AprimeB4CertainSlopes.ln.Fix.78.910.25.Rand.12.57quad.89);length(fixef(AprimeB4CertainSlopes.ln.Fix.78.910.25.Rand.12.57quad.89))
```

```
##                                      (Intercept) 
##                                      3.309408347 
##                                          slope12 
##                                      0.059623786 
##                                          slope25 
##                                      0.006734447 
##                                          slope57 
##                                      0.013517228 
##                                     I(slope57^2) 
##                                      0.019485180 
##                                          slope78 
##                                      0.006340490 
##                                          slope89 
##                                      0.046729394 
##                                         slope910 
##                                      0.030553171 
##                     Post_Block4_A_ExtremeCorrect 
##                                     -0.010030349 
##  slope78:Post_Block4_A_ExtremeCorrect_MeanCenter 
##                                     -0.258207801 
##  slope89:Post_Block4_A_ExtremeCorrect_MeanCenter 
##                                     -0.410467275 
## slope910:Post_Block4_A_ExtremeCorrect_MeanCenter 
##                                     -0.389103581
```

```
## [1] 12
```

DF=4 Wald Test for slopes moderated by A of Block 4.

```
contestMD(AprimeB4CertainSlopes.ln.Fix.78.910.25.Rand.12.57quad.89, ddf="Satterthwaite", L=rbind(c(0,0,0,0,0,0,0,0,1,0,0,0),
                                                                                                 c(0,0,0,0,0,0,0,0,0,1,0,0),
                                                                                                 c(0,0,0,0,0,0,0,0,0,0,1,0),
                                                                                                 c(0,0,0,0,0,0,0,0,0,0,0,1)))
```

```
##       Sum Sq    Mean Sq NumDF    DenDF  F value    Pr(>F)
## 1 0.07227587 0.01806897     4 62.28798 1.811726 0.1378242
```

F(4,62.28798)=1.811726, p=0.1378242, suggesting A in Block 4 is not a
significant moderator of cardiac vagal tone trends during a vigilance
task, break, or post-break vigilance task.

### Test how the median response time of hits (HitRTmd) in Block 4 moderates the time slopes associated with break and Block 4

Summary stats of HitRTmd in Block 4 (as reported in the journal
article).

```
hist(subset.data.frame(Downsel_HRV_Perf_TTU_Long_AllNeedTimeSubIDs,Downsel_HRV_Perf_TTU_Long_AllNeedTimeSubIDs$Timepoint=="T1")$HitRTmdB4)
```

```
boxplot(subset.data.frame(Downsel_HRV_Perf_TTU_Long_AllNeedTimeSubIDs,Downsel_HRV_Perf_TTU_Long_AllNeedTimeSubIDs$Timepoint=="T1")$HitRTmdB4)
```

```
boxplot.stats(subset.data.frame(Downsel_HRV_Perf_TTU_Long_AllNeedTimeSubIDs,Downsel_HRV_Perf_TTU_Long_AllNeedTimeSubIDs$Timepoint=="T1")$HitRTmdB4)
```

```
## $stats
## [1] 505.50 622.25 673.00 780.50 956.00
## 
## $n
## [1] 31
## 
## $conf
## [1] 628.0924 717.9076
## 
## $out
## numeric(0)
```

```
mean(subset.data.frame(Downsel_HRV_Perf_TTU_Long_AllNeedTimeSubIDs,Downsel_HRV_Perf_TTU_Long_AllNeedTimeSubIDs$Timepoint=="T1")$HitRTmdB4)
```

```
## [1] 695.6613
```

```
sd(subset.data.frame(Downsel_HRV_Perf_TTU_Long_AllNeedTimeSubIDs,Downsel_HRV_Perf_TTU_Long_AllNeedTimeSubIDs$Timepoint=="T1")$HitRTmdB4)
```

```
## [1] 109.3848
```

```
min(subset.data.frame(Downsel_HRV_Perf_TTU_Long_AllNeedTimeSubIDs,Downsel_HRV_Perf_TTU_Long_AllNeedTimeSubIDs$Timepoint=="T1")$HitRTmdB4)
```

```
## [1] 505.5
```

```
max(subset.data.frame(Downsel_HRV_Perf_TTU_Long_AllNeedTimeSubIDs,Downsel_HRV_Perf_TTU_Long_AllNeedTimeSubIDs$Timepoint=="T1")$HitRTmdB4)
```

```
## [1] 956
```

```
#center the performance metrics on their mean in order to make the moderating effects (if they are present) more interpretable
Downsel_HRV_Perf_TTU_Long_AllNeedTimeSubIDs["HitRTmdB4_MeanCenter"]<-Downsel_HRV_Perf_TTU_Long_AllNeedTimeSubIDs$HitRTmdB4-mean(subset.data.frame(Downsel_HRV_Perf_TTU_Long_AllNeedTimeSubIDs,Downsel_HRV_Perf_TTU_Long_AllNeedTimeSubIDs$Timepoint=="T1")$HitRTmdB4)

#build the theoretically motivated conditional growth curve model for performance differences between Block 3 and Block 4 (only interact with piecewise slopes that are touching the break)
HitRTmdB4CertainSlopes.ln.Fix.78.910.25.Rand.12.57quad.89<-lmer(data=Downsel_HRV_Perf_TTU_Long_AllNeedTimeSubIDs, REML=TRUE, 
                                                                formula=ln_RMSSD_ms~1+slope12+slope25+slope57+I(slope57^2)+slope78+slope89+slope910+
                                                                        HitRTmdB4+slope78:HitRTmdB4_MeanCenter+
                                                                        slope89:HitRTmdB4_MeanCenter+slope910:HitRTmdB4_MeanCenter+
                                                                      (1+slope12+slope57+I(slope57^2)+slope89|New_ID), control=lmerControl(optimizer="bobyqa")) #, optCtrl=list(method='NLOPT_LN_BOBYQA'))) #convergence issue minor, optCtrl=list(method='L-BFGS-B'))) #"Nelder_Mead", optCtrl=list(maxfun=5e5)))
#allFit(HitRTmdB4CertainSlopes.ln.Fix.78.910.25.Rand.12.57quad.89)

fixef(HitRTmdB4CertainSlopes.ln.Fix.78.910.25.Rand.12.57quad.89);length(fixef(HitRTmdB4CertainSlopes.ln.Fix.78.910.25.Rand.12.57quad.89))
```

```
##                   (Intercept)                       slope12 
##                  3.409312e+00                  5.962379e-02 
##                       slope25                       slope57 
##                  6.734447e-03                  1.351723e-02 
##                  I(slope57^2)                       slope78 
##                  1.948518e-02                  6.340490e-03 
##                       slope89                      slope910 
##                  4.672939e-02                  3.055317e-02 
##                     HitRTmdB4  slope78:HitRTmdB4_MeanCenter 
##                 -1.562648e-04                  1.495329e-05 
##  slope89:HitRTmdB4_MeanCenter slope910:HitRTmdB4_MeanCenter 
##                 -2.005142e-04                  4.628337e-05
```

```
## [1] 12
```

DF=3 Wald test for slopes moderated by median response time of hits
(HitRTmd) during Block 4.

```
contestMD(HitRTmdB4CertainSlopes.ln.Fix.78.910.25.Rand.12.57quad.89, ddf="Satterthwaite", L=rbind(c(0,0,0,0,0,0,0,0,1,0,0,0),
                                                                                                  c(0,0,0,0,0,0,0,0,0,1,0,0),
                                                                                                  c(0,0,0,0,0,0,0,0,0,0,1,0),
                                                                                                  c(0,0,0,0,0,0,0,0,0,0,0,1)))
```

```
##        Sum Sq     Mean Sq NumDF    DenDF  F value    Pr(>F)
## 1 0.007366955 0.001841739     4 57.33676 0.181522 0.9470718
```

F(4,57.33676)=0.181522, p=0.9470718, suggesting the median response
time of hits (HitRTmd) in Block 4 is not a significant moderator of
cardiac vagal tone trends during a vigilance task, break, or post-break
vigilance task.

### Test how A in Block 3 moderates the time slopes associated with the break and Block 4

```
#summary stats of this performance measure to report in the investigation of it being a time-invariant predictor in the growth curve model
hist(subset.data.frame(Downsel_HRV_Perf_TTU_Long_AllNeedTimeSubIDs,Downsel_HRV_Perf_TTU_Long_AllNeedTimeSubIDs$Timepoint=="T1")$Pre_Block3_A_ExtremeCorrect)
```

```
boxplot(subset.data.frame(Downsel_HRV_Perf_TTU_Long_AllNeedTimeSubIDs,Downsel_HRV_Perf_TTU_Long_AllNeedTimeSubIDs$Timepoint=="T1")$Pre_Block3_A_ExtremeCorrect)
```

```
boxplot.stats(subset.data.frame(Downsel_HRV_Perf_TTU_Long_AllNeedTimeSubIDs,Downsel_HRV_Perf_TTU_Long_AllNeedTimeSubIDs$Timepoint=="T1")$Pre_Block3_A_ExtremeCorrect)
```

```
## $stats
## [1] 0.75190 0.83365 0.87190 0.91700 0.99720
## 
## $n
## [1] 31
## 
## $conf
## [1] 0.8482472 0.8955528
## 
## $out
## numeric(0)
```

```
mean(subset.data.frame(Downsel_HRV_Perf_TTU_Long_AllNeedTimeSubIDs,Downsel_HRV_Perf_TTU_Long_AllNeedTimeSubIDs$Timepoint=="T1")$Pre_Block3_A_ExtremeCorrect)
```

```
## [1] 0.8764387
```

```
sd(subset.data.frame(Downsel_HRV_Perf_TTU_Long_AllNeedTimeSubIDs,Downsel_HRV_Perf_TTU_Long_AllNeedTimeSubIDs$Timepoint=="T1")$Pre_Block3_A_ExtremeCorrect)
```

```
## [1] 0.06257962
```

```
min(subset.data.frame(Downsel_HRV_Perf_TTU_Long_AllNeedTimeSubIDs,Downsel_HRV_Perf_TTU_Long_AllNeedTimeSubIDs$Timepoint=="T1")$Pre_Block3_A_ExtremeCorrect)
```

```
## [1] 0.7519
```

```
max(subset.data.frame(Downsel_HRV_Perf_TTU_Long_AllNeedTimeSubIDs,Downsel_HRV_Perf_TTU_Long_AllNeedTimeSubIDs$Timepoint=="T1")$Pre_Block3_A_ExtremeCorrect)
```

```
## [1] 0.9972
```

```
#center the performance metrics on their mean in order to make the moderating effects (if they are present) more interpretable
Downsel_HRV_Perf_TTU_Long_AllNeedTimeSubIDs["Pre_Block3_A_ExtremeCorrect_MeanCenter"]<-Downsel_HRV_Perf_TTU_Long_AllNeedTimeSubIDs$Pre_Block3_A_ExtremeCorrect-mean(subset.data.frame(Downsel_HRV_Perf_TTU_Long_AllNeedTimeSubIDs,Downsel_HRV_Perf_TTU_Long_AllNeedTimeSubIDs$Timepoint=="T1")$Pre_Block3_A_ExtremeCorrect)

#build the theoretically motivated conditional growth curve model for performance differences between Block 3 and Block 4 (only interact with piecewise slopes that are touching the break)
AprimeB3CertainSlopes.ln.Fix.78.910.25.Rand.12.57quad.89<-lmer(data=Downsel_HRV_Perf_TTU_Long_AllNeedTimeSubIDs, REML=TRUE, 
                                                              formula=ln_RMSSD_ms~1+slope12+slope25+slope57+I(slope57^2)+slope78+slope89+slope910+
                                                                        Pre_Block3_A_ExtremeCorrect+slope57:Pre_Block3_A_ExtremeCorrect_MeanCenter+
                                                                        I(slope57^2):Pre_Block3_A_ExtremeCorrect_MeanCenter+
                                                                      (1+slope12+slope57+I(slope57^2)+slope89|New_ID), control=lmerControl(optimizer="bobyqa")) #, optCtrl=list(method='NLOPT_LN_BOBYQA'))) #convergence issue is minor in grand scheme of life; optCtrl=list(method='L-BFGS-B'))) #"Nelder_Mead", optCtrl=list(maxfun=5e5)))

fixef(AprimeB3CertainSlopes.ln.Fix.78.910.25.Rand.12.57quad.89);length(fixef(AprimeB3CertainSlopes.ln.Fix.78.910.25.Rand.12.57quad.89))
```

```
##                                         (Intercept) 
##                                         2.318522081 
##                                             slope12 
##                                         0.059623786 
##                                             slope25 
##                                         0.006734447 
##                                             slope57 
##                                         0.013517228 
##                                        I(slope57^2) 
##                                         0.019485180 
##                                             slope78 
##                                         0.006340490 
##                                             slope89 
##                                         0.046729394 
##                                            slope910 
##                                         0.030553171 
##                         Pre_Block3_A_ExtremeCorrect 
##                                         1.120537456 
##      slope57:Pre_Block3_A_ExtremeCorrect_MeanCenter 
##                                        -0.588440701 
## I(slope57^2):Pre_Block3_A_ExtremeCorrect_MeanCenter 
##                                         0.135542077
```

```
## [1] 11
```

DF=3 Wald Test for slopes moderated by A in Block 3.

```
contestMD(AprimeB3CertainSlopes.ln.Fix.78.910.25.Rand.12.57quad.89, ddf="Satterthwaite", L=rbind(c(0,0,0,0,0,0,0,0,1,0,0),
                                                                                                 c(0,0,0,0,0,0,0,0,0,1,0),
                                                                                                 c(0,0,0,0,0,0,0,0,0,0,1)))
```

```
##       Sum Sq    Mean Sq NumDF    DenDF  F value    Pr(>F)
## 1 0.03328953 0.01109651     3 29.00177 1.106716 0.3623233
```

F(3,29.00058)=1.106712, p=0.3623258, suggesting A in Block 3 is not a
significant moderator of cardiac vagal tone trends during a vigilance
task, break, or post-break vigilance task.

### Test how the median response time of hits (HitRTmd) in Block 3 moderates the time slopes associated with the break and Block 4

```
#summary stats of this performance measure to report in the investigation of it being a time-invariant predictor in the growth curve model
hist(subset.data.frame(Downsel_HRV_Perf_TTU_Long_AllNeedTimeSubIDs,Downsel_HRV_Perf_TTU_Long_AllNeedTimeSubIDs$Timepoint=="T1")$HitRTmdB3)
```

```
boxplot(subset.data.frame(Downsel_HRV_Perf_TTU_Long_AllNeedTimeSubIDs,Downsel_HRV_Perf_TTU_Long_AllNeedTimeSubIDs$Timepoint=="T1")$HitRTmdB3)
```

```
boxplot.stats(subset.data.frame(Downsel_HRV_Perf_TTU_Long_AllNeedTimeSubIDs,Downsel_HRV_Perf_TTU_Long_AllNeedTimeSubIDs$Timepoint=="T1")$HitRTmdB3)
```

```
## $stats
## [1] 524.5 644.0 692.0 772.5 950.0
## 
## $n
## [1] 31
## 
## $conf
## [1] 655.5347 728.4653
## 
## $out
## [1] 1085
```

```
mean(subset.data.frame(Downsel_HRV_Perf_TTU_Long_AllNeedTimeSubIDs,Downsel_HRV_Perf_TTU_Long_AllNeedTimeSubIDs$Timepoint=="T1")$HitRTmdB3)
```

```
## [1] 721.1452
```

```
sd(subset.data.frame(Downsel_HRV_Perf_TTU_Long_AllNeedTimeSubIDs,Downsel_HRV_Perf_TTU_Long_AllNeedTimeSubIDs$Timepoint=="T6")$HitRTmdB3)
```

```
## [1] 118.3855
```

```
min(subset.data.frame(Downsel_HRV_Perf_TTU_Long_AllNeedTimeSubIDs,Downsel_HRV_Perf_TTU_Long_AllNeedTimeSubIDs$Timepoint=="T1")$HitRTmdB3)
```

```
## [1] 524.5
```

```
max(subset.data.frame(Downsel_HRV_Perf_TTU_Long_AllNeedTimeSubIDs,Downsel_HRV_Perf_TTU_Long_AllNeedTimeSubIDs$Timepoint=="T1")$HitRTmdB3)
```

```
## [1] 1085
```

```
#center the performance metrics on their mean in order to make the moderating effects (if they are present) more interpretable
Downsel_HRV_Perf_TTU_Long_AllNeedTimeSubIDs["HitRTmdB3_MeanCenter"]<-Downsel_HRV_Perf_TTU_Long_AllNeedTimeSubIDs$HitRTmdB3-mean(subset.data.frame(Downsel_HRV_Perf_TTU_Long_AllNeedTimeSubIDs,Downsel_HRV_Perf_TTU_Long_AllNeedTimeSubIDs$Timepoint=="T1")$HitRTmdB3)

#build the theoretically motivated conditional growth curve model for performance differences between Block 3 and Block 4 (only interact with piecewise slopes that are touching the break)
HitRTmdB3CertainSlopes.ln.Fix.78.910.25.Rand.12.57quad.89<-lmer(data=Downsel_HRV_Perf_TTU_Long_AllNeedTimeSubIDs, REML=TRUE, 
                                                                formula=ln_RMSSD_ms~1+slope12+slope25+slope57+I(slope57^2)+slope78+slope89+slope910+
                                                                        HitRTmdB3+slope57:HitRTmdB3_MeanCenter+
                                                                        I(slope57^2):HitRTmdB3_MeanCenter+
                                                                      (1+slope12+slope57+I(slope57^2)+slope89|New_ID), control=lmerControl(optimizer="nlminbwrap")) #, optCtrl=list(method='NLOPT_LN_BOBYQA'))) #convergence issue minor, optCtrl=list(method='L-BFGS-B'))) #"Nelder_Mead", optCtrl=list(maxfun=5e5)))
#allFit(HitRTmdB3CertainSlopes.ln.Fix.78.910.25.Rand.12.57quad.89)

fixef(HitRTmdB3CertainSlopes.ln.Fix.78.910.25.Rand.12.57quad.89);length(fixef(HitRTmdB3CertainSlopes.ln.Fix.78.910.25.Rand.12.57quad.89))
```

```
##                       (Intercept)                           slope12 
##                      2.8471557508                      0.0596237864 
##                           slope25                           slope57 
##                      0.0067344467                      0.0135172277 
##                      I(slope57^2)                           slope78 
##                      0.0194851801                      0.0063404904 
##                           slope89                          slope910 
##                      0.0467293939                      0.0305531714 
##                         HitRTmdB3      slope57:HitRTmdB3_MeanCenter 
##                      0.0006287898                     -0.0004565921 
## I(slope57^2):HitRTmdB3_MeanCenter 
##                      0.0002040881
```

```
## [1] 11
```

DF=3 Wald test for time slopes moderated by median response time of
hits (HitRTmd) in Block 4.

```
contestMD(HitRTmdB3CertainSlopes.ln.Fix.78.910.25.Rand.12.57quad.89, ddf="Satterthwaite", L=rbind(c(0,0,0,0,0,0,0,0,1,0,0),
                                                                                                  c(0,0,0,0,0,0,0,0,0,1,0),
                                                                                                  c(0,0,0,0,0,0,0,0,0,0,1)))
```

```
##       Sum Sq     Mean Sq NumDF    DenDF   F value    Pr(>F)
## 1 0.01903301 0.006344335     3 29.00208 0.6327505 0.5998652
```

F(3,29.00221)=0.6327505, p=0.5998651, suggesting the median response
time of hits (HitRTmd) in Block 3 is not a significant moderator of
cardiac vagal tone trends during a vigilance task, break, or post-break
vigilance task.

## Conditional growth curve model - Task load

Finally, we need a conditional growth curve model to test how task
load moderates the height and trajectory of the growth curve model?
Theory would suggest it matters, so we build a conditional growth curve
model that is theoretically motivated, i.e., one that has an additive
and a cross-level predictor for all time slopes.

```
Taskload.ln.Fix.78.910.25.Rand.12.57quad.89<-lmer(data=HRV_TTU_Long_AllNeedTimeSubIDs, REML=TRUE,
                                            formula=ln_RMSSD_ms~(1+slope12+slope25+slope57+I(slope57^2)+slope78+slope89+slope910)+                            TaskLoadCondition+slope12:TaskLoadCondition+slope25:TaskLoadCondition+slope57:TaskLoadCondition+I(slope57^2):TaskLoadCondition+                                                 slope78:TaskLoadCondition+slope89:TaskLoadCondition+slope910:TaskLoadCondition+
                                                    (1+slope12+slope57+I(slope57^2)+slope89|New_ID), control = lmerControl(optimizer = "bobyqa"))
summary(Taskload.ln.Fix.78.910.25.Rand.12.57quad.89, ddf="Satterthwaite"); llikAIC(Taskload.ln.Fix.78.910.25.Rand.12.57quad.89, chkREML=FALSE)
```

```
## Linear mixed model fit by REML. t-tests use Satterthwaite's method [
## lmerModLmerTest]
## Formula: ln_RMSSD_ms ~ (1 + slope12 + slope25 + slope57 + I(slope57^2) +  
##     slope78 + slope89 + slope910) + TaskLoadCondition + slope12:TaskLoadCondition +  
##     slope25:TaskLoadCondition + slope57:TaskLoadCondition + I(slope57^2):TaskLoadCondition +  
##     slope78:TaskLoadCondition + slope89:TaskLoadCondition + slope910:TaskLoadCondition +  
##     (1 + slope12 + slope57 + I(slope57^2) + slope89 | New_ID)
##    Data: HRV_TTU_Long_AllNeedTimeSubIDs
## Control: lmerControl(optimizer = "bobyqa")
## 
## REML criterion at convergence: -187.8
## 
## Scaled residuals: 
##      Min       1Q   Median       3Q      Max 
## -2.37613 -0.53284  0.02196  0.51884  2.56325 
## 
## Random effects:
##  Groups   Name         Variance Std.Dev. Corr                   
##  New_ID   (Intercept)  0.20224  0.4497                          
##           slope12      0.08148  0.2854   -0.41                  
##           slope57      0.09651  0.3107    0.18 -0.70            
##           I(slope57^2) 0.01399  0.1183   -0.28  0.74 -0.99      
##           slope89      0.01596  0.1263    0.37 -0.06 -0.25  0.11
##  Residual              0.01015  0.1007                          
## Number of obs: 310, groups:  New_ID, 31
## 
## Fixed effects:
##                                   Estimate Std. Error         df t value
## (Intercept)                       3.336801   0.111773  29.000054  29.853
## slope12                           0.080818   0.076205  31.865734   1.061
## slope25                           0.005802   0.010925 149.007731   0.531
## slope57                          -0.076131   0.095674  33.792033  -0.796
## I(slope57^2)                      0.065598   0.040904  34.561695   1.604
## slope78                          -0.018961   0.034548 149.007742  -0.549
## slope89                           0.001477   0.046179  54.321276   0.032
## slope910                          0.027208   0.034548 149.007748   0.788
## TaskLoadConditionH               -0.080149   0.166323  29.000053  -0.482
## slope12:TaskLoadConditionH       -0.046929   0.113397  31.865730  -0.414
## slope25:TaskLoadConditionH        0.002065   0.016257 149.007740   0.127
## slope57:TaskLoadConditionH        0.198507   0.142368  33.792024   1.394
## I(slope57^2):TaskLoadConditionH  -0.102108   0.060867  34.561681  -1.678
## slope78:TaskLoadConditionH        0.056024   0.051409 149.007744   1.090
## slope89:TaskLoadConditionH        0.100202   0.068716  54.321277   1.458
## slope910:TaskLoadConditionH       0.007407   0.051409 149.007748   0.144
##                                 Pr(>|t|)    
## (Intercept)                       <2e-16 ***
## slope12                            0.297    
## slope25                            0.596    
## slope57                            0.432    
## I(slope57^2)                       0.118    
## slope78                            0.584    
## slope89                            0.975    
## slope910                           0.432    
## TaskLoadConditionH                 0.634    
## slope12:TaskLoadConditionH         0.682    
## slope25:TaskLoadConditionH         0.899    
## slope57:TaskLoadConditionH         0.172    
## I(slope57^2):TaskLoadConditionH    0.102    
## slope78:TaskLoadConditionH         0.278    
## slope89:TaskLoadConditionH         0.151    
## slope910:TaskLoadConditionH        0.886    
## ---
## Signif. codes:  0 '***' 0.001 '**' 0.01 '*' 0.05 '.' 0.1 ' ' 1
```

```
## $logLik
## 'log Lik.' 93.90202 (df=32)
## 
## $AICtab
##         AIC         BIC      logLik    deviance    df.resid 
## -123.804042   -4.233728   93.902021 -187.804042  278.000000
```

```
fixef(Taskload.ln.Fix.78.910.25.Rand.12.57quad.89); length(fixef(Taskload.ln.Fix.78.910.25.Rand.12.57quad.89))
```

```
##                     (Intercept)                         slope12 
##                     3.336800886                     0.080817555 
##                         slope25                         slope57 
##                     0.005801710                    -0.076131236 
##                    I(slope57^2)                         slope78 
##                     0.065598330                    -0.018960813 
##                         slope89                        slope910 
##                     0.001476846                     0.027208300 
##              TaskLoadConditionH      slope12:TaskLoadConditionH 
##                    -0.080149178                    -0.046929059 
##      slope25:TaskLoadConditionH      slope57:TaskLoadConditionH 
##                     0.002065346                     0.198507313 
## I(slope57^2):TaskLoadConditionH      slope78:TaskLoadConditionH 
##                    -0.102107690                     0.056024315 
##      slope89:TaskLoadConditionH     slope910:TaskLoadConditionH 
##                     0.100202070                     0.007406501
```

```
## [1] 16
```

DF=8 Wald test for all time slopes moderated by task load.

```
contestMD(Taskload.ln.Fix.78.910.25.Rand.12.57quad.89, ddf="Satterthwaite", L=rbind(c(0,0,0,0,0,0,0,0,1,0,0,0,0,0,0,0), 
                                                                                    c(0,0,0,0,0,0,0,0,0,1,0,0,0,0,0,0),
                                                                                    c(0,0,0,0,0,0,0,0,0,0,1,0,0,0,0,0),
                                                                                    c(0,0,0,0,0,0,0,0,0,0,0,1,0,0,0,0),
                                                                                    c(0,0,0,0,0,0,0,0,0,0,0,0,1,0,0,0),
                                                                                    c(0,0,0,0,0,0,0,0,0,0,0,0,0,1,0,0),
                                                                                    c(0,0,0,0,0,0,0,0,0,0,0,0,0,0,1,0),
                                                                                    c(0,0,0,0,0,0,0,0,0,0,0,0,0,0,0,1)))
```

```
##      Sum Sq    Mean Sq NumDF    DenDF  F value    Pr(>F)
## 1 0.1268668 0.01585835     8 51.11151 1.563155 0.1593645
```

F(8,51.11151)=1.563155, p=0.1593645, suggesting there is not enough
evidence to suggest that task load significantly moderates cardiac vagal
tone trends during a vigilance task, break, or post-break vigilance
task.
